# Major adverse limb events in patients with femoro-popliteal and below-the-knee peripheral arterial disease treated with either sirolimus-coated balloon or standard uncoated balloon angioplasty: a structured protocol summary of the “SirPAD” randomized controlled trial

**DOI:** 10.1186/s13063-022-06242-8

**Published:** 2022-04-21

**Authors:** Stefano Barco, Tim Sebastian, Davide Voci, Rolf Peter Engelberger, Alexandru Grigorean, Erik Holy, Claudia Leeger, Mario Münger, Daniel Périard, Eliane Probst, Rebecca Spescha, Ulrike Held, Nils Kucher

**Affiliations:** 1 Department of Angiology, University Hospital Zurich, Zurich, Switzerland; 2 HFR Fribourg Cantonal Hospital: HFR Fribourg Hopital cantonal, Fribourg, Switzerland; 3 Department of Biostatistics at Epidemiology, Biostatistics and Prevention Institute, University of Zurich, Zurich, Switzerland

**Keywords:** Peripheral arterial occlusive disease, Atherosclerotic disease, Common iliac artery, Intermittent claudication, Critical limb ischemia, Endovascular, Stenting, Covered stent, Sirolimus

## Abstract

**Background:**

Peripheral arterial disease is a progressive atherosclerotic disease with symptoms ranging from an intermittent claudication to acute critical limb ischemia and amputations. Drug-coated balloons and stents were developed to prevent neo-intimal proliferation and restenosis after percutaneous transluminal angioplasty. Randomized controlled trials showed that drug-coated, notably paclitaxel-coated, devices reduce restenosis, late lumen loss, and the need for target lesion re-vascularization compared with uncoated ones. However, the size of these trials was too small to prove superiority for “hard” clinical outcomes. Moreover, available studies were characterized by too restrictive eligibility criteria. Finally, it remains unclear whether paclitaxel-coated balloons may impair long-term survival. Alternative drug-coated balloons, the so-called limus-based analogs, have been approved for clinical use in patients with peripheral arterial disease. By encapsulating sirolimus in phospholipid drug nanocarriers, they optimize adhesion properties of sirolimus and provide better bioavailability.

**Methods:**

In this investigator-initiated all-comer open-label phase III randomized controlled trial, we will evaluate whether sirolimus-coated balloon angioplasty is non-inferior and eventually superior, according to a predefined hierarchical analysis, to uncoated balloon angioplasty in adults with infra-inguinal peripheral arterial disease requiring endovascular angioplasty. Key exclusion criteria are pregnancy or breastfeeding, known intolerance or allergy to sirolimus, and participation in a clinical trial during the previous 3 months. The primary efficacy outcome is the composite of two clinically relevant non-subjective “hard” outcomes: unplanned major amputation of the target limb and endovascular or surgical target lesion re-vascularization for critical limb ischemia occurring within 1 year of randomization. The primary safety outcome includes death from all causes.

**Discussion:**

By focusing on clinically relevant outcomes, this study will provide useful information on the efficacy and safety of sirolimus-coated balloon catheters for infra-inguinal peripheral arterial disease in a representative (“all-comer”) population of unselected patients. As regulatory agencies had raised safety concerns in patients exposed to paclitaxel-coated devices (versus uncoated ones), collect mortality data up to 5 years after randomization will be collected.

**Trial registration:**

ClinicalTrials.gov NCT04238546

**Supplementary Information:**

The online version contains supplementary material available at 10.1186/s13063-022-06242-8.

## Administrative information

Note: the numbers in curly brackets in this protocol refer to SPIRIT checklist item numbers. The order of the items has been modified to group similar items (see http://www.equator-network.org/reporting-guidelines/spirit-2013-statement-defining-standard-protocol-items-for-clinical-trials/).

**Table:** 

Title {1}	Major adverse limb events in patients with femoro-popliteal and below-the-knee peripheral arterial disease treated with either sirolimus-coated balloon or standard uncoated balloon angioplasty. A structured protocol summary of the ´SirPAD´ randomized controlled trial.
Trial registration {2a and 2b}	Clinicaltrial.gov: NCT04238546 SNCTP: 000003692
Protocol version {3}	Protocol v. 2.0 (1.Nov.2021, second amendement)
Funding {4}	This investigator-initiated study is funded by the University Hospital Zurich, Rämistrasse 100, CH 8091 Zürich - Switzerland This investigator-initiated study has received an unrestricted grant from Concept Medical. Concept Medical did not contribute to the preparation of the study protocol. Concept Medical Mariner ST, STE 200 Tampa, FL 33609 United States of America
Author details {5a}	Stefano Barco, Tim Sebastian, Davide Voci, Rolf Peter Engelberger, Alexandru Grigorean, Erik Holy, Claudia Leeger, Mario Münger, Daniel Périard, Eliane Probst, Rebecca Spescha, Ulrike Held, Nils Kucher
Name and contact information for the trial sponsor {5b}	Prof. Dr. med Nils Kucher University Zurich University Hospital Zurich Clinic of Angiology Rämistrasse 100 CH 8091 Zurich Switzerland Phone: + 41 44 255 4082 Fax: + 41 44 255 45 10 E-Mail: nils.kucher@usz.ch
Role of sponsor {5c}	The sponsor is entirely responsible for the design and conduct of the study, statistical analysis, interpretation of results, and drafting of the manuscript. The sponsor’s academic research organization was responsible for data collection and monitoring. The findings of this clinical trial including the interim analysis will be published in a scientific journal or presented at a scientific meeting under the ultimate responsibility of the sponsor. The funders did not contribute to the preparation of the present study protocol and have no role in the design, conduct, analysis, and presentation of the study.

## Introduction

### Background and rationale {6a}

Peripheral arterial disease (PAD) is a progressive atherosclerotic disease with symptoms ranging from intermittent claudication (lower limb pain while walking) to critical limb ischemia (resting pain and tissue damage). The prevalence of PAD is rapidly growing in aging societies and PAD continues to be a serious public health problem [1, 2]. The majority of symptomatic PAD patients present with atherosclerotic lesions located in the femoro-popliteal (thigh) arteries, and endovascular therapy is the primary choice if the stenosis or occlusion involves < 25 cm of the vessel [3]. A minority of symptomatic PAD patients would present with infra-popliteal (distal or below-the-knee) lesions: in these patients, the endovascular treatment remains challenging. Percutaneous transluminal angioplasty (PTA) is a minimal-invasive technique which requires a vascular access point; this is typically obtained by puncturing and introducing a sheath into the common femoral artery. It aims at resolving intraluminal obstruction of blood vessels by utilizing wires, balloon catheters, and stents.

Drug-coated balloons (DCB) and drug-eluting stents (DES) were developed to prevent neo-intimal proliferation and restenosis after PTA, an objective that may be achieved by the local application of either cytostatic (e.g., paclitaxel—a cytoskeletal disruptor) or immunosuppressive (e.g., sirolimus/everolimus—both mTOR inhibitors) substances on the vessel wall. Both mechanisms inhibit the proliferation of arterial smooth muscle cells. Indeed, data from randomized controlled trials from the past decade showed that drug-coated (mainly paclitaxel-coated) devices led to a substantial reduction in restenosis rates, late lumen loss, and incidence of target lesion re-vascularization compared with that of uncoated ones [3–9]. A few factors, however, limit the generalizability of these findings: among others, (i) the small size of these trials; (ii) the substantial heterogeneity of the study populations across studies and, at the same time, too restrictive eligibility criteria in individual studies; and (iii) the adoption of surrogate (and rather subjective) outcomes, which may be difficult to be objectively adjudicated in the setting of an open-label trial.

Although the short-term effects appeared promising based on imaging outcomes and patency rates, the results of a recent meta-analysis of 28 trials showed an increased 2-year mortality in the group of patients treated with paclitaxel-coated balloons [10] with a consequent warning and safety concern from the Food and Drug Administration (FDA) concerning paclitaxel-based devices [11]. Alternative drug candidates to paclitaxel-coated balloon catheters are the so-called limus-based analogs, which own cytostatic properties and are characterized by a wider therapeutic window. Recently, a novel balloon catheter has been CE-certified: it encapsulates sirolimus in phospholipid drug nanocarriers to improve adhesion properties of sirolimus and to provide better bioavailability [12].

The aim of the present investigator-initiated, phase III, open-label randomized controlled trial is to compare the efficacy, as defined by a composite of clinically relevant non-subjective “hard” outcomes (major amputation and target lesion re-vascularization for critical limb ischemia), of sirolimus-coated vs. uncoated balloon angioplasty for peripheral arterial disease in patients scheduled for infra-inguinal re-vascularization and selected based on a very limited number of inclusion criteria (all comers) aiming at maximizing its external validity.

### Investigational medical device

The registered name of the investigated medical device is “The Magic Touch PTA Sirolimus Coated Balloon Catheter”: this product is approved for PTA of infra-inguinal and infra-poplital lesions. The coating of the Magic Touch PTA utilizes Nanolute® technology to deliver polymer-free sirolimus encapsulated in a generally recognized as safe (GRAS) phospholipid excipient. The amphiphilic properties of the phospholipid allow the sirolimus to remain encapsulated and protected from degradation for a time sufficient to ensure treatment of the target lesion. The drug is delivered to the vessel wall upon contact of the expanded balloon with the arterial lumen.

**Fig. 1:**
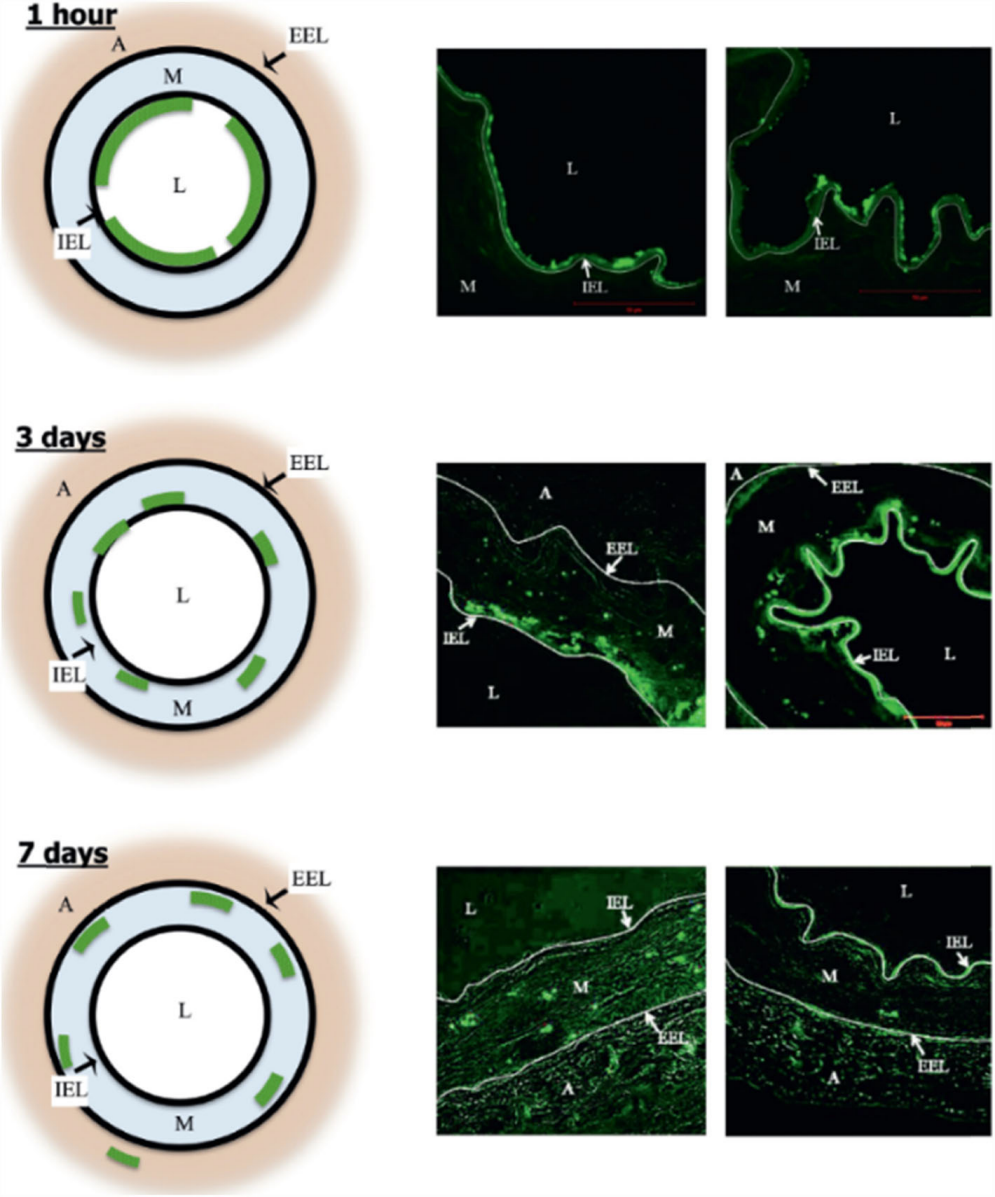
Temporal penetration of labeled sirolimus nanoparticles

Sirolimus is a macrolide antibiotic, produced by the bacterium *Streptomyces hygroscopicus*, well known for its antifungal, immunosuppressant, antitumor, and anti-inflammatory properties. It has been already used and approved for the treatment of the coronary arteries with DES and it is anticipated that its properties will also reduce the tendency for restenosis. The figure depicts the temporal penetration of the DFT-labeled sirolimus nanoparticles after balloon inflation, as assessed by confocal microscopy (Fig. 1). The panels on the left show a diagrammatic representation and the mid and right panels the actual cross-sectional images [12].

**Fig. 2:**
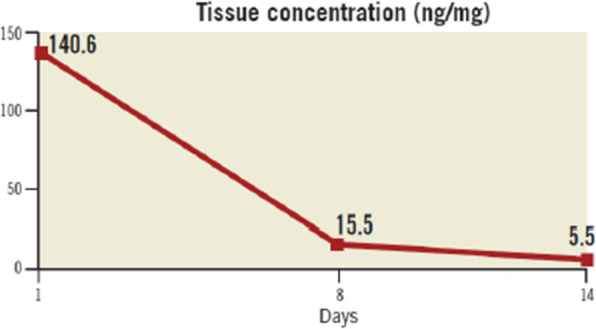
Tissue concentration of sirolimus after single 60-s inflation

In an animal study, evaluating blood and tissue levels in rabbits after treatment with Magic Touch found that blood concentration decreased rapidly after a single 60-s deployment, while tissue concentration was still detectable after 2 weeks (Fig. 2).

The XTOSI study was the first-in-human prospective interventional study to investigate the efficacy and safety of a sirolimus-coated balloon in patients with peripheral arterial disease (PAD) undergoing endovascular re-vascularization. The results of the interim analysis (*N* = 38) have been presented at the 2019 TCT Congress in San Francisco. The primary outcomes were freedom from clinically drive target lesion re-vascularization (TLR) at 12 months; freedom from major adverse events (MAE) at 30 days, a composite of freedom from device- and procedure-related mortality, and major target limb amputation through to 30 days; and freedom from clinically driven TLR within 6 months post-index procedure.

Freedom from MAE at 30 days was 95% [*n* = 36/38], where two patients underwent major limb (below knee) amputation due to severe sepsis of the foot post-angioplasty. Freedom from device- and procedure-related mortality was 100%. Freedom from clinically drive TRL within 6 months post-index procedure was available for 11 patients and was reported to be 91% [*n* = 10/11]. Based on the results of the study to date, Magic Touch PTA has a good safety profile at 30 days.

## Objectives {7}

### Primary objective

The primary objective of the SirPAD trial is to evaluate whether the use of sirolimus-coated balloon catheters is non-inferior to uncoated balloon catheters in infra-inguinal angioplasty to prevent 1-year major adverse limb events (MALE), including unplanned major amputation of the target limb and target lesion re-vascularization for critical limb ischemia in a representative population of patients with PAD (“all comers”). If the criterion for non-inferiority is confirmed, the study will test according to pre-specified criteria for hierarchical analysis whether sirolimus-coated catheters are superior to uncoated catheters for important secondary outcomes and for the primary efficacy outcome.

### Secondary objective

The secondary objective is to assess the safety of uncoated balloon catheters and test whether their efficacy is maintained across important subgroups of patients (heterogeneity of treatment effect analysis), also for surrogate outcomes of efficacy.

## Trial design {8}

SirPAD trial is a single-center, randomized controlled (1:1), open-label, non-inferiority trial with a hierarchical analysis allowing testing for superiority.

## Methods: participants, interventions, and outcomes

### Study setting {9}

The study will be conducted at the Clinic of Angiology of the University Hospital Zurich and at the Department of Internal Medicine and Division of Angiology of the Cantonal Hospital Fribourg (Switzerland). The project sites are highly specialized consultative health care providers for both in- and outpatients in their two regions. In Zurich, the Clinic for Angiology provides a 24-h emergency service including catheterization laboratory standby and performs approximately 800 peripheral intervention per year. The involvement of additional centers is considered very difficult in a “all-comer” setting, as several competing trials would have been running in parallel at these sites, therefore leading to a pre-selection of potential candidates for participation.

### Eligibility criteria {10}

#### Inclusion criteria

- Age > 18 years
- Patients requiring endovascular angioplasty for PAD (target lesion) located below the inguinal ligament (predefined clinical and angiographic criteria are listed below). A target lesion is defined as the main lesion considered responsible for the patient’s signs and symptoms, fulfilling the following angiographic criteria:
  - Stenosis (lumen compromise ≥50%) in at least a single plane of the femoro-popliteal arterial segment including the femoral, deep femoral, and/or popliteal artery or a femoro-popliteal bypass, or
  - Stenosis (lumen compromise ≥50%) of the below-the-knee arterial segment including the tibio-peroneal trunk and/or the anterior tibial, peroneal, or posterior tibial artery, or a below-the-knee bypass
- Written informed consent obtained from participant or legal guardian prior to randomization; in patients requiring emergency interventional treatment who are temporarily not capable of providing informed consent, consent will be subsequently obtained after the procedure if strict conditions apply. These include the assessment of the presumed will and patient decree and require the allocation of an independent physician.

#### Exclusion criteria

The presence of any one of the following exclusion criteria will lead to exclusion of the participant:

- Pregnancy, breastfeeding, or planned pregnancy within the trial period or women of childbearing potential not using an adequate method of contraception
- Patients with known intolerance or allergy to sirolimus
- Participation in this or other clinical trials during the previous 3 months

### Who will take informed consent? {26a}

We planned four scenarios to obtain patient’s informed consent.

The *first scenario* is foreseen as the most frequent one in clinical practice. The patient is scheduled for an elective PTA procedure and is able to give written consent. The study investigator explains to the potential participant the nature of the study, its purposes, the procedures involved, the expected duration, the potential risks and benefits, and any discomfort or deviation from routine management it may entail. The study participants will be provided with a patient information leaflet and an informed consent form describing this study and reporting enough information for participants in order to make an informed decision about their participation. The participant will read and have enough time to consider the statement before signing and dating the informed consent form, and he/she will be given a copy of the signed document. The written consent is collected before the procedure. The consent form will be signed and dated by the investigator, or his/her designee, and retained as part of the study records.

In the *second scenario*, a vulnerable or fragile patient is scheduled for elective procedures and a legal guardian has to evaluate and sign the dedicated informed consent form, acting in the best patient’s interest.

In the *third scenario*, a vulnerable patient with a legal guardian requires a rescue re-vascularization that cannot further be delayed. In the first instance, the legal guardian is being contacted and asked for the will of the patient. If the legal guardian is not contactable, the will of the patient can be assumed either by contacting relatives of the patient or eventually by consulting the patient’s decree. In any case, a dedicated informed consent for an independent physician must be signed and the patient is from that moment enrolled under reserve. A formal dedicated consent must be obtained as soon as the legal guardian is contactable. If the legal representative rejects the consent, it represents a major violation of the protocol, causing the exclusion of the patient from the study and stopping of any subsequent study procedure.

In the *fourth scenario*, a patient is temporarily not able to consent and requires a rescue re-vascularization. Also, in this case, the will of the patient can be consulted in the patient’s decree or by contacting relatives. In any case, a dedicated informed consent must be signed from an independent physician and the formal consent has to be obtained as soon as the patient is conscious and accountable.

### Additional consent provisions for collection and use of participant data and biological specimens {26b}

No biological specimens will be collected during the trial.

## Interventions

All interventions will be performed according to standard treatment of care. In both groups, provisional stent placement with commercially available (CE-certified) bare metal stents may be used after balloon dilatation with either a CE-certified uncoated balloon catheter (comparator) or Magic Touch PTA device (intervention) in case of severe arterial dissection and/or persisting (flow-limiting) lumen compromise following balloon angiography necessitating a stent for mechanical scaffold. Bare metal stents that will be used include, but are not limited to, EverFlex Self-expanding Peripheral Stent (Medtronic) 6–8 mm diameter, 20–200 mm length.

### Explanation for the choice of comparators {6b}

The comparator consists of any available CE-certified uncoated balloon catheter with application in PAD approved for patient use in Switzerland. These are used in daily clinical practice interchangeably, in the absence of a standard of care and often depending on the timing of supply. We have decided to allow the use of all uncoated products to minimize the extent of screening failures and avoid head-to-head comparisons between the interventional product and a specific medical device, since there is no standard of care in this field that may be used as the comparator. All study devices used have been approved for the current indication and are already in routine clinical use. In the control group, upon angiographic determination of the target lesion, plain old balloon angioplasty (POBA, uncoated) will be performed using a balloon diameter corresponding to the reference vessel diameter. All balloons will be inflated for a duration of 120 s at nominal pressure. Nominal pressure is defined as the inflation pressure required to reach the device-specific diameter.

### Intervention description {11a}

The Magic Touch PTA device is a sirolimus-coated balloon catheter indicated for the treatment of stenotic lesions of infra-inguinal or infra-popliteal arteries and consists of two components: (1) a PTA balloon catheter coated with (2) polymer-free formulation containing the sirolimus drug as an active ingredient in an encapsulated phospholipid excipient. The drug dose per mm^2^ balloon surface is 1.27 μg. POBA is performed for vessel preparation (pre-dilatation). In addition, in the experimental group, in a second step, patients will receive target lesion treatment with the Magic Touch PTA sirolimus-coated balloon (Concept Medical B.V., Hoevelaken, The Netherlands). All balloons will be inflated for a duration of 120 s at nominal pressure. Nominal pressure is defined as the inflation pressure required for reaching the device-specific diameter.

### Criteria for discontinuing or modifying allocated interventions {11b}

Modifying allocated intervention for a participant requesting a certain treatment regimen is not planned. Patients who request a certain strategy (uncoated or sirolimus-coated) for specific reasons will be classified as a screening failure if not randomized in the study.

### Strategies to improve adherence to interventions {11c}

Since the treatment period immediately follows randomization and ends with the termination of the re-vascularization procedure, and the investigational product does not remain in situ, there is no need to track participant compliance. The compliance of the interventionist (investigator) to the use of the respective study devices will be monitored. The erroneous use of a drug-coated balloon catheter in a patient allocated to the uncoated group and vice versa, or the use of any drug-eluting stents will be documented as a major protocol violation.

### Relevant concomitant care permitted or prohibited during the trial {11d}

Provisional stent placement is used in case of severe arterial dissection and/or persisting (flow-limiting) lumen compromise following balloon angiography necessitating a stent for mechanical scaffold. Concomitant in- and outflow disease may be treated upon discretion of the interventionist using uncoated devices. All concomitant treatments have to be recorded in the CRF.

### Provisions for post-trial care {30}

Enrolled patients are covered by indemnity for negligent harm through the standard indemnity arrangements of the University Hospital Zurich. The University Hospital of Zurich has insurance to cover for non-negligent harm associated with the protocol. Since all study interventions are regarded “standard of care,” the sponsor will not provide any intervention, benefits, or other care outside boundaries of standard medical care after the trial is completed.

### Outcomes {12}

#### Primary outcome

The primary efficacy outcome is a composite of two major adverse limb events (MALE), assessed within 1 year of randomization: an unplanned major amputation of the target limb and an endovascular or surgical target lesion re-vascularization for critical limb ischemia.

An unplanned major amputation is defined as any amputation above the ankle on the target limb, which was not planned or not expectable at the time of screening or randomization. Patients with scheduled amputation undergoing re-vascularization to improve wound healing are referred to as planned amputation and will not count for the primary outcome. Critical limb ischemia is primarily defined according to a Fontaine stage (stages III and IV).

#### Secondary outcomes

The secondary outcomes include the following:

- A composite of unplanned (major or minor) index-limb amputations or any target lesion re-vascularization within 365 days after enrolment (tested in the hierarchical analysis if criteria for non-inferiority are fulfilled). An unplanned minor amputation is defined as an amputation below or at the level of the ankle, which was not planned or not expected at the time of randomization
- Clinical improvement by > 1 Rutherford category through 30–180 days
- Any target lesion re-vascularization performed within 365 days after enrolment
- Target lesion re-vascularization for non-critical limb ischemia performed within 365 days, where non-critical limb ischemia is defined as patients with Fontaine stages I–II
- Target lesion re-vascularization for critical limb ischemia within 365 days after enrolment, where critical limb ischemia is defined as patients with Fontaine stages III–IV
- Target limb re-vascularization within 365 days after enrolment
- Unplanned minor amputation at target limb performed within 365 days after enrolment
- Unplanned major amputation at target limb performed within 365 days after enrolment
- Any unplanned amputation

Freedom from target lesion/limb re-vascularization is defined as the percentage of patients without the occurrence of re-intervention (surgical or interventional) at the target lesion/limb irrespective of any re-intervention out of the target lesion.

#### Safety outcomes

The safety outcomes include the following:

- Death from all causes within 30 days, 180 days, 1 year, 2 years, and 5 years
- Serious adverse events (SAEs) during initial hospitalization, within 30–180 days, and within 365 days
- Serious adverse device-related events (SADE) during initial hospitalization
- A composite of all-cause death and MALE within 30 days

The occurrence of safety outcomes, including vital status of patients, will be verified at each scheduled visit or time point, including hospital discharge, interim visit (day 30–180), 1-year visit (day 365 + 30), and study termination (year 5), or if it is being reported by patients at any time during follow-up.

The primary sources for verifying the patients’ vital status will be in-person contact with the relatives and treating physicians, internal and external medical reports (including discharge letters including the vital status and causes of death), and administrative vital registration data (“Zivilstand”).

A serious adverse event (SAE) is represented by an adverse event that led to any of the following:

(a) Death

(b) Serious deterioration in the health of the subject that resulted in any of the following:

(i) Life-threatening illness or injury

(ii) Permanent impairment of a body structure or a body function

(iii) Hospitalization or prolongation of patient hospitalization

(iv) Medical or surgical intervention to prevent life-threatening illness or injury or permanent impairment

to a body structure or a body function

(v) Chronic disease

(c) Fetal distress, fetal death, or a congenital physical or mental impairment or birth defect

The definitions of SADE, adverse device effect (ADE), and unanticipated serious adverse device effect (USADE) are reported in the study protocol (Supplementary Material).

### Participant timeline {13}

**Fig. 3:**
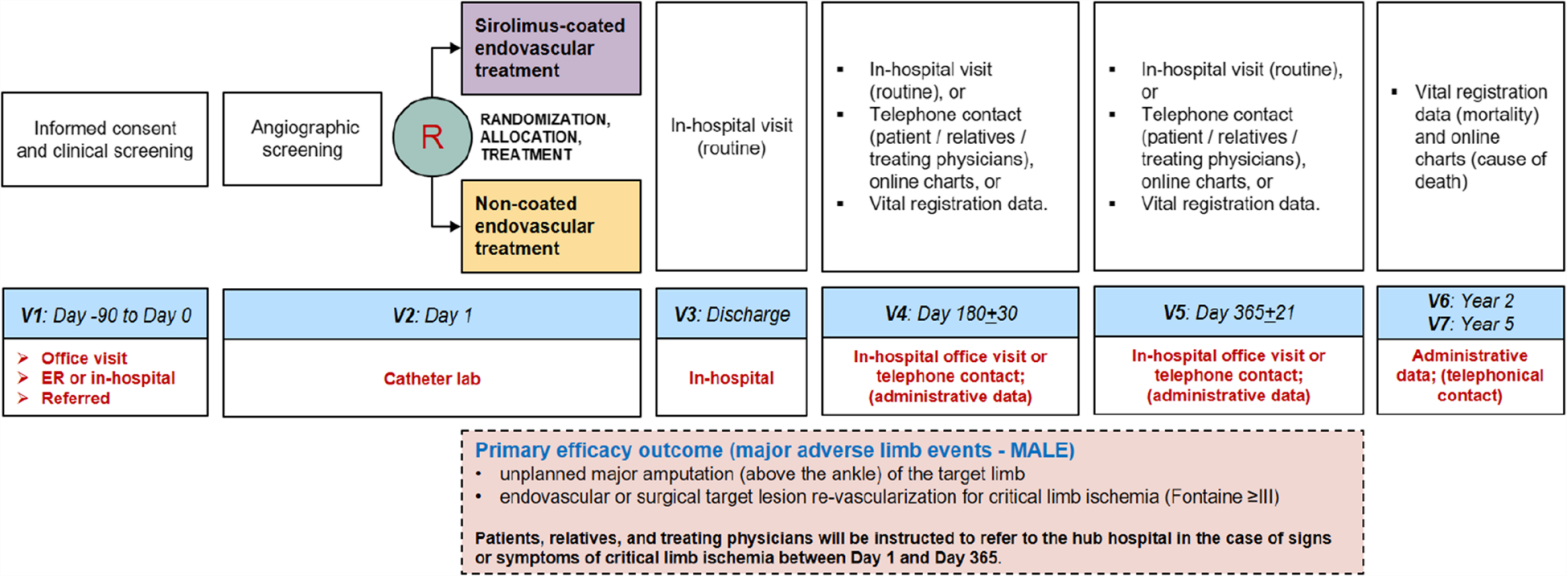
Participant timeline

The participant timeline is shown in Fig. 3.

### Sample size {14}

The sample size calculation is based on previous studies reporting MALE rates in PAD patients after re-vascularization procedures. These studies were heterogeneous in study design, patient selection, techniques (proportion of patients with endovascular approach), and findings [13–24]. Hess et al. reported a MALE incidence of 10% at 12 months among 381,415 re-vascularized patients that were included in the Premier Healthcare Database between April 2009 and September 2014 [19]. In addition, there were two larger interventional studies reporting 30-day MALE incidence after surgical or endovascular therapy of symptomatic PAD patients. Fashandi et al. reported a MALE incidence of 3.2% in patients with claudication at 1 month following therapy [21]. Mehaffey et al. reported a MALE incidence of 12.2% in patients with critical limb ischemia (CLI) [22]. The COMPASS trial estimated a MALE incidence of 2.0% among 6.341 patients with PAD at 21 months, of which 35% were asymptomatic. The MALE incidence was 3.6% among the subgroup of patients with previous re-vascularization procedures [24]. A subgroup analysis of the Fourier trial estimated a MALE incidence of 1.5% among 3.642 patients with PAD (31% asymptomatic) at 12 months [23]. In the XTOSI study, the 6-month amputation-free survival was 90% in patients receiving sirolimus-coated balloon catheters.

Based on these studies, assuming a 10% event rate (MALE) within 12 months in both the control and intervention group, and a non-inferiority margin of 5% expressed as absolute risk difference, a total of 1132 patients (566 patients per treatment group) allow to show non-inferiority of the intervention group with a power of 80% and a type I error rate of *α* = 2.5% one-sided. Assuming a drop-out rate of 5%, including randomization failures, a total of 1200 patients will be randomized in the study.

### Recruitment {15}

**Fig. 4:**
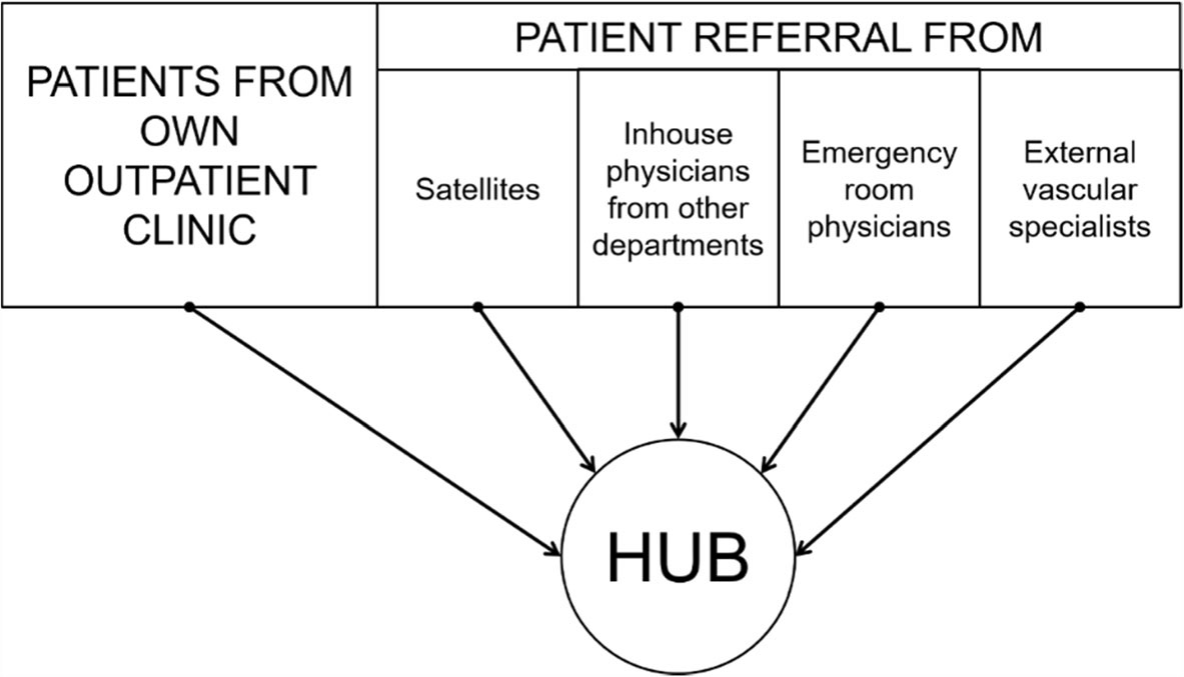
Study recruitment

The strategy for study recruitment is displayed in Fig. 4. The screening phase will be composed of two phases: (i) clinical screening and (ii) angiographic screening. Both phases will take place at the study centers and involve patients with peripheral arterial disease referred for evaluation of re-vascularization or direct referral for re-vascularization by study center physicians or other treating physicians, as per routine procedures.

The Clinic of Angiology of the University Hospital of Zurich and the Division of Angiology of the Cantonal Hospital Fribourg currently performs together approximately 1050 peripheral interventions per year. Therefore, assuming that more than 80% of the potentially eligible patients will be finally enrolled in the study, the patient recruitment is expected to last no longer than 30 months with the Last-Patient-Out visit for the 5-year cumulative mortality analysis planned in 2028.

## Assignment of interventions: allocation

### Sequence generation {16a}

After the angiographic screening and definition of the target lesion, but prior to any therapeutic intervention, each eligible patient will be assigned to one of the two open treatment arms of this trial by an online randomization tool. This generation of the randomization sequence was generated before the start of the study outside the platform, REDCap, serving for data collection and used other software (R). Randomization will be stratified according to the stage of disease, namely elective re-vascularization (Fontaine stages I–II) vs. critical rescue re-vascularization (Fontaine stages III–IV), and study center. A block randomization will be done using an online random tool (REDCap, Vanderbilt University, v9 1.0).

### Concealment mechanism {16b}

Allocation will be concealed and treatment will be done immediately thereafter during the same angiographic session.

### Implementation {16c}

The allocation sequence was generated using a statistical software by a data analyst from the Clinical Trial Center of the University of Zurich. Participants will be enrolled and assigned to the intervention by the study physicians and investigators from the two study sites.

## Assignment of interventions: blinding

### Who will be blinded {17a}

The trial is conducted as an open-label study.

### Procedure for unblinding if needed {17b}

The trial is conducted as an open-label study.

## Data collection and management

### Plans for assessment and collection of outcomes {18a}

#### Assessment of primary and secondary outcomes

For the primary analysis, the primary efficacy outcome will be assessed within 1 year from randomization. Formal visits are planned at hospital discharge, between days 30 and 180, and at 1 year (flowchart). The same applies for all the secondary and safety outcomes. This strategy will allow minimal burden for the patients, who will not undergo additional visits that are not part of routine clinical and sonographic follow-up at our center. In particular, given the “hard” nature of the primary efficacy outcome, the ease of assessment, and the fact that these patients are usually referred to the center where the intervention was performed, the risk of reporting bias would be minimal.

The relatives will be instructed to contact the study investigators in the presence of novel signs of symptoms or complications any time after enrolment. A 24/7 telephone number for emergencies is available.

#### Assessment of safety outcome

Survival status and the cause of death will be assessed during follow-up if a patient misses a scheduled visit or cannot be contacted telephonically, as well as at the end of clinical follow-up (year 1) and at the time of long-term follow-up (year 2 and year 5).

In the case a patient is not followed at the study centers, the treating physicians, the patient, and the relatives will be instructed to contact the study investigators if a severe adverse event is suspected. All patients will be instructed to return to the hospital as soon as new symptoms occur, at which time information about the time of onset, intensity, and duration of the event will be collected, and objective tests and clinical examination may be performed.

The Data Safety Monitoring committee is responsible for the oversight and safety monitoring of the study. Regular evaluations of safety data will be after inclusion of multiples of 150 patients. The committee advises the sponsor regarding the continuing safety of the trial subjects.

### Plans to promote participant retention and complete follow-up {18b}

In order to collect the data for the primary outcome, the patients will undergo a routine visit. If a patient will not undergo or will not be able to attend routine in-hospital visit, or if no information will be spontaneously obtained from the treating physicians between day 30 and day 180, the investigators will verify whether the patient has been recently admitted in another department of the study center, the investigator tries to contact the patient or the relatives by phone call. The phone interviews will be performed by specially trained staff of the University of Zürich and will be based on a standardized list of questions integrated in the eCRF.

This strategy will allow minimal burden for the patients, who will not undergo additional visits that are not part of routine follow-up. In particular, given the “hard” nature of the primary efficacy outcome, the ease of assessment, and the fact that these patients are usually referred to the center where the intervention was performed, the risk of reporting bias would be minimal. In order to limit the amount of missing data points, we have implemented a sequential system to optimize data collection: (i) Information will be collected at the time of the planned visit; (ii) telephone contacts of patients/relatives/treating physicians; (iii) education of patients to refer to the study center if any new symptoms occur; and (iv) assessment of online medical charts and “Zivilstand.”

### Data management {19}

For the data entry, the data management, as well as data storage and security, an Internet-based secure data base REDCap (Vanderbilt University, v9 1.0) developed in agreement to the Good Clinical Practice (GCP) guidelines provided by the Clinical Trials Center (CTC) Zurich will be used for this study. Data will be stored in an oracle data base, which will be installed on a local server at the University Hospital of Zurich and routine back-ups will be performed in order to prevent data loss.

The data entry via REDCap application occurs web-based and the data will be transferred encrypted. Each user will receive a personalized REDCap account. The possibly audit-trail information will be stored in the REDCap application for each data entry, data change, or data deletion including the time and name of the user. In addition, data entered into the eCRF will undergo automatic plausibility checks where possible. An overview for the implemented plausibility checks will be available for each version of the eCRF.

In order to promote data quality and detect possible errors at an early time, regular monitoring visits at the investigator’s site prior to the start and during the study will be performed. Also, the sponsor-investigator organizes additional professional independent monitoring for the study, collaborating also with the Clinical Trials Center of the University Hospital Zurich. Audits and inspections to guarantee and control quality data may be conducted by the Competent Authority or Competent Ethics Committee, respectively.

### Confidentiality {27}

Direct access to online source documents will be permitted for purposes of monitoring, audits, and inspections. The investigator-sponsor, all investigators, the members of the clinical event committee, the study coordinator, and the bio-statistician will have access to the investigation plan and data sets during and after the study. The bio-statistician will have access to the statistical code.

### Plans for collection, laboratory evaluation, and storage of biological specimens for genetic or molecular analysis in this trial/future use {33}

No biological specimens will be collected during the trial.

## Statistical methods

### Statistical methods for primary and secondary outcomes {20a}

In the primary analysis of this trial, the absolute risk difference for MALE at 12-month follow-up between treatment groups will be estimated, together with its 2-sided 95% confidence interval. The trial statistician will perform the analyses after termination of the trial. If the overall event rate estimated at interim analysis is > 10%, an unblinded estimation of the risk difference and confidence interval will be calculated. The confidence level *α* will then be spent such that the overall type I error is preserved for an interim analysis and a final analysis, using a Lan-DeMets spending function. At interim, the confidence level will therefore be 99.95% and for the final analysis, it will be 95.05%.

If the null hypothesis concerning the primary objective is rejected (and the primary objective, non-inferiority, is thus established), further confirmatory statistical tests on primary and secondary endpoints will be performed using a pre-specified hierarchical order based on the clinical importance of the considered outcomes:

1. Superiority for the composite of unplanned (major or minor) index-limb amputations or any target lesion re-vascularization within 365 days after enrolment (*α* = 2.5% one-sided)

2. Superiority for MALE within 365 days after enrolment (*α* = 2.5% one-sided)

No additional reduction or splitting of the single *α* levels is necessary for this reason since the predefined ordering avoids any choice in the assessment (Guideline on multiplicity issues in clinical trials, European Medicines Agency; EMA/CHMP/44762/2017).

### Interim analyses {21b}

A single interim analysis with information rate of 50%, i.e., 300 patients per arm, 600 in total, is planned. If at interim the overall event rate exceeds a pre-specified threshold of 10%, the event rate will be estimated in both treatment groups (unblinded).

Following D’Agostino et al. [25] “for efficacy reasons one can argue that there is no real ethical issue with seeing the [non-inferiority] trial to completion from an efficacy perspective”. However, “interim analyses are also important in a non-inferiority trial for safety reasons, either to ensure the experimental treatment is not doing more harm than good (R1), or that it is superior with regard to specific adverse events (R2).”

#### Monitoring for efficacy

If the overall event rate at interim analysis is higher than the threshold of 10%, the event rate in both treatment groups will be estimated and the between-group risk difference (RD) and its confidence interval will be estimated. To preserve the overall type I error for one interim analysis and the final analysis, the *α* of 0.05 (2-sided) is spent using the Lan-DeMets spending function. At the interim analysis, a 99.95% confidence interval is calculated; at the final analysis, a 95.05% confidence interval is calculated. It is unlikely that at interim analysis, non-inferiority can be declared unless the experimental treatment is superior to active control [26].

#### Monitoring for safety

Safety concerns in this study are twofold: (R1) the efficacy endpoint (MALE) may differ substantially between treatment groups and (R2) mortality may differ between treatment groups. On safety grounds, termination of the trial may be recommended after the interim analysis. However, such assessments may potentially have implications for falsely concluding that there is an adverse effect. No statistical reasons for stopping the trial at interim analysis are declared.

Notation:

*δ*_Int_: between-group risk difference *p*_exp_ − *p*_con_ at 12 months at interim analysis for MALE

*d*_Int_: between-group risk difference *π*_exp_ − *π*_con_ at 12 months at interim analysis for mortality

exp = experimental group, coated devices

con = active control group, uncoated devices

**Table:** 

Scenario	Reasoning	Interpretation	Action
Interim analysis after 600 patients followed up 12 months (50% of total sample size)			
*p*_total_ ≤ 0.10	Verify assumptions about nuisance parameters	Obtain an estimate of overall MALE event rate at interim. Assumptions about nuisance parameters seem to be realistic.	Continue trial without any changes.
*p*_total_ > 0.10	Safety	Differential MALE event rates between treatment groups could be causing higher event rate than anticipated.	Unblinded estimation of the MALE event rates in each treatment group.
(1) *δ*_Int_ = 0	Efficacy	*p*_exp_ = *p*_con_	Calculate Bayesian predictive probabilities for successful termination of trial. Continue the trial unless otherwise stated by DMB. Based on interim results, the DMB may suggest a sample size increase.
(2) *δ*_Int_ > 0	Efficacy: R1	MALE event rate in the experimental group is considerably higher than in the control group.	Quantification of “how much higher”. DMB discusses stopping for safety reasons.
(3) *δ*_Int_ < 0	Efficacy: R1	MALE event rate in the experimental group is considerably lower than in the control group.	Quantification of “how much lower”. DMB discusses stopping for safety reasons.

**Table:** 

Continuous safety evaluation, after multiples of 150 patients at 12-month follow-up			
*d*_Int_ ≠ 0	Safety: R2	Unblinded estimation of mortality rates. Assess if mortality rates are different between treatment groups.	How much higher mortality rate in one group compared to the other after 12-month follow-up?

### Methods for additional analyses (e.g., subgroup analyses) {20b}

Multivariable logistic regression for the outcome MALE event will be used to estimate an adjusted treatment effect, given the stratification variables. The following subgroups are pre-specified for analysis of heterogeneity of effects: age ≥ 75 vs. < 75, men vs. women, patients with elective re-vascularization (Fontaine I–II) vs. critical re-vascularization (Fontaine > II), patients with 1 vs. more than 1 level intervention, de novo lesion vs. restenosis, total occlusion vs. partial occlusion, distal vs. proximal lesions. If there is evidence for a differential treatment effect within subgroups (i.e., *p*-value of interaction test < 0.05), the treatment effect will be reported within subgroups as odds ratios or hazard ratios for time-to-event outcomes.

### Methods in analysis to handle protocol non-adherence and any statistical methods to handle missing data {20c}

Patients who discontinue the clinical trial participation prematurely after randomization will not be replaced, whereas patients who drop out before randomization will be replaced. For the analysis of outcomes with missing data, the missingness generating mechanism will be evaluated and multiple imputation techniques will be applied.

### Plans to give access to the full protocol, participant-level data, and statistical code {31c}

The full protocol is summarized here and will be published together with the final publication; participant-level dataset and statistical code are available upon reasonable request.

## Oversight and monitoring

### Composition of the coordinating center and trial steering committee {5d}

*Steering committee*: Stefano Barco (co-PI), Tim Sebastian, Davide Voci, Rolf Peter Engelberger, Alexandru Grigorean, Erik Holy, Mario Münger, Daniel Périard, Ulrike Held (study statistician), Nils Kucher (PI).

*Coordinating center*: Rebecca Specha, Claudia Leeger, Eliane Probst, Stephanie Roth, Yulia Butscheid.

### Composition of the data monitoring committee, its role, and reporting structure {21a}

*Data and Safety Monitoring Board (DSMB) members*: Marc Righini (chair), Luca Valerio, Nicolas Diehm.

*Monitoring institution*: Clinical Trials Center – Monitoring, Universitätsspital Zürich, Rämistrasse 100 / MOU2, 8091 Zürich.

The DSMB will review the results of the safety assessment conducted after the completion of the 12-month follow-up of multiples of 150 patients. The DSMB can recommend continuation or termination of the study based on the evaluation of the results. An internal Clinical Events Committee made up of two clinicians will categorize the components of the primary outcome. An external Clinical Events Committee was deemed not necessary given the “hard” nature of the primary outcome, a composite of endovascular or surgical target lesion re-vascularization for critical limb ischemia and unplanned major amputation of the target limb. The Clinical Events Committee will meet regularly to review and adjudicate all clinical events. The investigator’s site will collaborate with the Clinical Trials Center (CTC) of the University Hospital Zurich (Rämistrasse 100/MOU2, 8091 Zürich) to ensure regular monitoring. According to the CTC’s Monitoring SOP, the extent and nature of monitoring activities based on the objective and design of the study are defined in a separate study-specific monitoring plan.

### Adverse event reporting and harms {22}

All SAEs, device deficiencies, and health hazards that require measures are reported to the sponsor by the PI (or authorized designee) within 24 h after becoming aware of the event. Device deficiencies are assessed regarding their potential to lead to an SAE. DD are assessed regarding their potential to lead to an SAE. The other study site (Fribourg) will report on SAE via RedCAP, and the sponsor will receive automatic email notification with the possibility of logging in and access the original study documents and clinical information, as provided by the investigators.

The sponsor reports to the CEC promptly any serious adverse event which has a causal relation with the MD, comparator, or procedure/test method or where a causal relation appears to be possible (Art. 33 ClinOMD). In order to ensure prompt notification, the sponsor may initially submit an incomplete notification. If safety and health hazards that require measures must be taken immediately during the conduct of the investigation, the sponsor notifies the CEC within 2 days of these measures and the circumstances which made them necessary (Art. 34 ClinO-MD). An Annual Safety Report (ASR; Art. 35 ClinO-MD) is submitted by the sponsor to the CEC, yearly. The ASR contains a list of all SADEs and DDs and a report on their degree of seriousness, causal relationship with the MD and procedure and on subjects’ safety. Other reporting is done according to provisions of MD vigilance as per Art. 87-90 MDR (Art. 33 abs 4.b ClinO-MD) and Art. 67 MedDO.

### Frequency and plans for auditing trial conduct {23}

Regular monitoring visits at the investigator’s site prior to the start and during the study will help to follow up the progress of the clinical study, to assure utmost accuracy of the data, and to detect possible errors at an early time point. The sponsor-investigator organizes professional independent monitoring for the study. The investigator’s site will collaborate with the Clinical Trials Center (CTC) of the University Hospital Zurich (Rämistrasse 100 / MOU2, 8091 Zürich) to ensure regular monitoring. According to the CTC’s Monitoring SOP, the extent and nature of monitoring activities based on the objective and design of the study will be defined in a separate study-specific monitoring plan. All original data including all patient files, progress notes, and copies of laboratory and medical test results will be available for monitoring. Source data includes study eligibility, inclusion/exclusion criteria, baseline demographics, device accountability, primary efficacy endpoint, primary safety endpoints, and SAE/SADE. A quality assurance audit/inspection of this study may be conducted by the Competent Authority or Competent Ethics Committee, respectively.

### Plans for communicating important protocol amendments to relevant parties (e.g., trial participants, ethical committees) {25}

Any modifications to the study protocol potentially influencing the conduct of the study, may benefit the patient or may affect patient safety, including changes of study objectives, study design, patient population, sample sizes, study procedures, or significant administrative aspects, will require a formal amendment to the protocol. Such an amendment will be approved by the Ethics Committee/institutional review board prior to implementation and notified to the health authorities in accordance with local regulations. Approvements of protocol amendments will be communicated to researchers and participants in written.

## Dissemination plans {31a}

The investigators agree on the use of the results of this clinical trial for the national and international registration of the product for specific indications, publication, and information for medical and industrial professionals. If necessary, the authorities will be notified of the investigator’s name, address, qualifications, and extent of involvement. The findings of this clinical trial including the interim analysis will be published in a scientific journal or presented at a scientific meeting. Publication of clinical trial results requires mutual agreement between the investigators and the sponsor. Any publication of the clinical trial data by the sponsor or investigators will be wholly consistent with the integrated report in accordance with the ethical principles of the Declaration of Helsinki. All publications will follow the Uniform Requirements for Manuscripts Submitted to Biomedical Journals (www.icmje.org, October 2008).

## Discussion

PAD is a progressive atherosclerotic disease of the lower extremities. Every year more than 200 million people globally suffer from PAD, which negatively affects their quality of life and increases the risk of amputation and death. The best strategy today for the management of patients with symptomatic PAD is represented by a timely arterial re-vascularization. With the advent of percutaneous transluminal angioplasty (PTA), endovascular interventions have been becoming the first-line therapy for the majority of patients. Standard balloon angioplasty is associated with over 90% technical success rates, but on the other side is still characterized by a considerable rate of restenosis due to local inflammation, neo-intimal hyperplasia, and barotrauma. In order to mitigate these effects, drug-coated devices, either with cytostatic drugs, such as paclitaxel, or anti-proliferative drug, such as sirolimus, have been introduced in the market.

By focusing on clinically relevant outcomes, such as major amputation and target lesion re-vascularization for critical limb ischemia, the present investigator-initiated, phase III, open-label randomized controlled trial will provide useful information on the efficacy and safety of sirolimus-coated balloon catheters for infra-inguinal peripheral arterial disease. The population for the study will be selected on a very limited number of inclusion criteria, in order to have a representative population of unselected patients (“all-comer”), with the aim of maximizing external validity. As regulatory agencies had raised in the past safety concerns in patients exposed to specific drug-coated devices, long-term mortality data will be collected.

## Trial status

The first patient has been enrolled in the Sir-PAD study on 3 November 2020. As to 22 December 2021, a total of 396 patients have been enrolled at the Clinic of Angiology of the University Hospital Zurich. The site initiation visit of the second center (Department of Internal Medicine and Division of Angiology, Cantonal Hospital Fribourg) is planned in January 2022. The completion of patient recruitment (last patient in) is expected in Q4 2023. The completion of the 5-year follow-up (last patient last visit) is expected by the end of 2028.

## Supplementary Information

**Additional file 1:** The latest version of the study protocol.

## Data Availability

All the investigators will have full access to the final trial dataset. Essential documents, as well as any patient files and source data, must be retained for at least 10 years after the regular end or a premature termination of the respective study (KlinV Art. 45). For data entry, data management, data storage, and security, an Internet-based secure data base REDCap (Vanderbilt University, v9 1.0) developed in agreement to the Good Clinical Practice (GCP) guidelines provided by the Clinical Trials Center (CTC) Zurich will be used for this study.

## Abbreviations

PAD: Peripheral arterial disease
DCB: Drug-coated balloons
DES: Drug-eluting stents
PTA: Percutaneous transluminal angioplasty
FDA: Food and Drug Administration
GRAS: Generally recognized as safe
TLR: Target lesion re-vascularization
MAE: Major adverse events
MALE: Major adverse limb events
POBA: Plain old balloon angioplasty
SAEs: Serious adverse events
SADE: Serious adverse device-related events
CLI: Critical limb ischemia
CRF: Case report forms
ADE: Adverse device effect
SADE: Serious adverse device effect
GCP: Good Clinical Practice
CTC: Clinical Trials Center
