# Supplementary material for: Major adverse limb events in patients with femoro-popliteal and below-the-knee peripheral arterial disease treated with either sirolimus-coated balloon or standard uncoated balloon angioplasty: a structured protocol summary of the “SirPAD” randomized controlled trial
Source: Trials. 2022 Apr 21;23:334. doi: 10.1186/s13063-022-06242-8 (PMC9027348; doi:10.1186/s13063-022-06242-8)
Supplement: Supplementary file 1 — Additional file 1: The latest version of the study protocol. [file 13063_2022_6242_MOESM1_ESM.pdf]

Clinical Investigation Plan

Major adverse limb events in patients with femoro-popliteal and below-the-knee peripheral arterial disease treated with either sirolimus-coated balloon or standard uncoated balloon angioplasty.

The ´SirPAD´ randomized controlled trial

**Study Type:**	Randomized controlled trial with Investigational Medical Devices (IMD)
**Study Categorization:**	Clinical Trial with IMD Category A1
**Study Registration:**	Clinical Trials.gov: NCT04238546
**Study Identifier:**	SNCTP000003692 | BASEC2020-00080
**Sponsor, Sponsor-Investigator and Principal Investigator:**	Prof. Dr. med Nils Kucher<br>University Zurich<br>University Hospital Zurich<br>Clinic of Angiology<br>Rämistrasse 100<br>CH 8091 Zürich<br>Switzerland<br>Phone: +41 44 255 4082<br>Fax: +41 44 255 45 10<br>E-Mail: nils.kucher@usz.ch
**Investigational Product:**	MagicTouch PTA sirolimus drug-coated balloon catheter (Concept Medical B.V., Hoevelaken, The Netherlands)
**Investigation plan Version and Date:**	v. 2.0; 01.11.2021

CONFIDENTIAL

The information contained in this document is confidential and the property of Clinic of Angiology at the University Hospital Zurich. The information may not - in full or in part - be transmitted, reproduced, published, or disclosed to others than the applicable Independent Ethics Committee(s) and Competent Authority(ies) without prior written authorization from Clinic of Angiology at the University Hospital Zurich, except to the extent necessary to obtain informed consent from those participants who will participate in the study.

SIGNATURE PAGE

**Study Title**	Major adverse limb events in patients with femoro-popliteal and below-the-knee peripheral arterial disease treated with either sirolimus-coated balloon or standard uncoated balloon angioplasty. The ´SirPAD´ randomized controlled trial
**Short Title**	SirPAD Trial

Sponsor-Investigator (Principal Investigator):

The Sponsor-Investigator and trial statistician have approved the investigation plan version, and confirm hereby to conduct the study according to the investigation plan, current version of the World Medical Association Declaration of Helsinki, ISO 14155 norm, and the local legally applicable requirements.

Sponsor-Investigator

Prof. Dr. med Nils Kucher
University Zurich
University Hospital Zurich
Clinic of Angiology
Rämistrasse 100
CH 8091 Zürich
Switzerland

Phone: +41 44 255 4082
E-Mail: nils.kucher@usz.ch

ZH 4.11.2021

Place/Date **Signature**

Trial Statistican

Prof. Dr. Ulrike Held
University of Zurich
Dept. of Biostatistics
Epidemiology, Biostatistics and Prevention Institute
Hirschengraben 84
8001 Zurich
Switzerland

Phone +41 44 634 45 39
E-Mail: ulrike.held@uzh.ch

Zürich, 4.11.2021

U. Held

Place/Date **Signature**

Local Principal Investigator at study site:

I have read and understood this trial protocol and agree to conduct the trial as set out in this study protocol, the current version of the World Medical Association Declaration of Helsinki, ICH-GCP guidelines and the local legally applicable requirements.

**Site**	HFR Fribourg – Hôpital Cantonal / Kantonsspital<br>Service d'Angiologie / Angiologie<br>Chemin des Pensionnats<br>1708 Fribourg<br>Switzerland
**Principal Investigator**	PD Dr. med. Rolf Engelberger

Fribourg 4.11.2021
Place/Date

PD Dr Rolf Engelberger
Médecin adjoint
Service d'angiologie
HFR Fribourg
hôpital fribourgeois freiburger spital
T 026 306 20 20 F 026 306 20 21
Signature

TABLE OF CONTENTS

SIGNATURE PAGE 2
ABBREVIATIONS 16
SUMMARY OF THE REVISION HISTORY IN CASE OF AMENDMENTS 17
1 STUDY SCHEDULE....18
2 STUDY ADMINISTRATIVE STRUCTURE 19
2.1 Sponsor-Investigator 19
2.2 Investigators....19
2.3 Statistician ("Biostatistician") 20
2.4 Monitoring Institution 20
2.5 Data Safety Monitoring Officer 20
2.6 Study Coordinator 20
3 ETHICAL AND REGULATORY ASPECTS....21
3.1 Study Registration....21
3.2 Competent Ethics Committee....21
3.3 Competent authority (CA)....21
3.4 Ethical Conduct of the Study 21
3.4.1 Compliance with the EN ISO 14155 standard 22
3.5 Declaration of Interest 22
3.6 Patient Information and Informed Consent 22
3.7 Participant Privacy and Confidentiality 25
3.8 Early Termination of the Study 25
3.9 Investigation Plan Amendments....25
4 INTRODUCTION....27
4.1 Background and Rationale 27
4.2 Investigational Medical Device and Indication 28
4.3 Preclinical Evidence 31
4.4 Clinical Evidence to Date 32
4.5 Explanation for Choice of Comparator....33
4.6 Risk / Benefits 33
4.7 Justification of Choice of Study Population....34
5 STUDY OBJECTIVES....34
5.1 Primary Objective....34
5.2 Secondary Objective 34
6 STUDY OUTCOMES 35
6.1 Primary Outcome 35
6.2 Secondary Outcomes....35
6.3 Safety Outcomes....35
7 STUDY DESIGN AND COURSE OF STUDY 37
7.1 General Study Design and Justification of the Design 37
7.2 Study Duration and Study Schedule....41

7.3 Methods of Minimizing Bias 41
7.3.1 Randomization 41
7.3.2 Other Methods of Minimizing Bias 41
7.4 Unblinding Procedures (Code break) 41
8 STUDY POPULATION 42
8.1 Eligibility Criteria 42
8.1.1 Inclusion Criteria 42
8.1.2 Exclusion Criteria 42
8.2 Recruitment and Screening 42
8.2.1 Clinical screening 43
8.2.2 Angiographic screening 43
8.2.3 Assignment to Study Groups 44
8.3 Criteria for Withdrawal/ Discontinuation of Participants 44
9 STUDY INTERVENTION 46
9.1 Identity of Investigational Medical Device 46
9.1.1 Experimental Intervention 46
9.1.2 Control Intervention 47
9.1.3 Packaging, Labelling and Supply (Re-Supply) 48
9.1.4 Storage Conditions 48
9.2 Administration of Experimental and Control Interventions 48
9.2.1 Experimental Intervention 48
9.2.2 Control Intervention 48
9.3 Modifications of Device Application 49
9.4 Compliance with Study Intervention 49
9.5 Data Collection and Follow-up for Withdrawn Participants 49
9.6 Trial Specific Preventive Measures 49
9.7 Concomitant Interventions 49
9.8 Medical Device Accountability 49
9.9 Return or Destruction of Medical Device 49
10 STUDY ASSESSMENTS 50
10.1 Study Flow Chart(s)/Table of Study Procedures and Assessments 50
10.2 Assessments of Outcomes 52
10.2.1 Assessment of Primary Outcome 52
10.2.2 Assessment of the Components of the Secondary Outcomes 53
10.2.3 Assessment of Safety Outcomes 53
10.2.4 Assessments in Participants Who Prematurely Stop the Study 54
10.3 Procedures at Each Visit 54
10.3.1 Visit 1 or Clinical Screening Visit 54
10.3.2 Visit 2 or Angiography Screening and Baseline Visit 54
10.3.3 Visit 3 or Discharge 54
10.3.4 Visit 4 or Day 180 (+ 30) Visit 55
10.3.1 Visit 5 or Day 365 (+ 21) Visit 55
10.3.2 Year-2 and Year-5 Assessment (Vital Status) 55
11 SAFETY 56

11.1 Definition of (Serious) Adverse Events and Other Safety Related Events 56
11.2 Categorization 57
11.3 Recording of Serious Adverse Events and Other Safety Related Events 57
11.4 Reporting of Serious Adverse Events and Other Safety Related Events 57
11.5 Follow up of Serious Adverse Events 58
12 STATISTICAL METHODS 58
12.1 Hypothesis 58
12.2 Determination of Sample Size 59
12.3 Statistical Criteria of Termination of Trial 59
12.4 Planned Analyses 59
12.4.1 Datasets to be Analyzed, Analysis Populations 60
12.4.2 Primary Analysis 60
12.4.3 Secondary Analyses 61
12.4.4 Interim efficacy and safety Analyses 61
12.4.5 Deviation(s) from the Original Statistical Plan 62
12.5 Handling of Missing Data and Drop-Outs 62
13 ELIGIBILITY OF THE PROJECT SITE 63
14 DATA QUALITY ASSURANCE AND CONTROL 64
14.1 Data Handling and Record Keeping / Archiving 64
14.1.1 Case Report Forms 64
14.1.2 Specification of Source Documents 64
14.1.1 Record Keeping / Archiving 65
14.2 Data Management 65
14.2.1 Data Management System 65
14.2.2 Data Security, Access and Back-up 65
14.2.3 Analysis and Archiving 65
14.2.4 Electronic and Central Data Validation 66
14.3 Monitoring 66
14.4 Audits and Inspections 66
14.5 Confidentiality, Data Protection 66
14.6 Data and safety monitoring board 66
15 PUBLICATION AND DISSEMINATION POLICY 67
16 FUNDING AND SUPPORT 68
16.1 Funding 68
16.2 Other Support 68
17 INSURANCE 69
18 APPENDICES 70
18.1 Fontaine classification 70
18.2 Rutherford classification for chronic limb ischemia 70
18.3 Rutherford classification for acute limb ischemia 71
19 REFERENCES 72

STUDY SYNOPSIS

**Sponsor-Investigator**	Prof. Dr. med Nils Kucher<br>University Zurich<br>University Hospital Zurich<br>Clinic of Angiology<br>Rämistrasse 100<br>CH 8091 Zürich<br>Switzerland<br>Phone: +41 44 255 4082<br>Fax: +41 44 255 45 10<br>E-Mail: nils.kucher@usz.ch
**Study Title**	Major adverse limb events in patients with femoro-popliteal and below-the-knee peripheral arterial disease treated with either sirolimus-coated or uncoated balloon. The ´SirPAD´ randomized controlled trial
**Short Title / Study ID**	SirPAD
**Investigation Plan Version and Date**	2.0 (01.11.2021)
**Trial registration**	Clinical Trials.gov: NCT04238546<br>SNCTP000003692 | BASEC2020-00080
**Study category and Rationale**	Clinical study with IMD Category A1
**Background and Rationale**	Peripheral artery disease (PAD) is a progressive atherosclerotic disease with symptoms ranging from intermittent claudication (IC) to critical limb ischemia (CLI). The majority of symptomatic PAD patients present with atherosclerotic lesions located in the femoro-popliteal arteries and endovascular therapy is the primary choice if the stenosis/occlusions involve <25 cm of the vessel. A minority of symptomatic PAD patients would present with infra-popliteal (distal or below-the-knee) lesions: in these patients, the endovascular treatment is challenging.<br>Drug-coated balloons (DCB) and drug-eluting stents (DES) were developed to prevent neo-intimal proliferation and restenosis after percutaneous transluminal angioplasty (PTA), an objective which had been achieved by the local application of either cytostatic (e.g. paclitaxel - a cytoskeletal disruptor) or immunosuppressive (e.g. sirolimus/everolimus - both mTOR inhibitors) substances on the vessel wall.<br>Over the past decade, a few randomized controlled trials (RCT) compared the efficacy and safety of drug-coated (mainly paclitaxel-coated) devices vs. that of uncoated ones, and demonstrated a significant reduction in restenosis rates, late lumen loss, and incidence of target lesion re-vascularization. However, the size of these trials was often too small to draw firm conclusions concerning major clinical outcomes. Moreover, substantial heterogeneity of the study populations and too restrictive eligibility criteria limited their external validity, leading to a difficult interpretation of the results of later meta-analyses. Indeed, these trials adopted as the primary outcome surrogate (and rather subjective) outcomes, such as vessel patency and target limb re-vascularization, which may be difficult to objectively adjudicate in the setting of an open-label trial, rather than ´hard´ objective clinical endpoints, such as major

	amputation or urgent revascularization due to critical limb ischemia.<br>Moreover, despite the short-term effects appeared promising based on imaging outcome, he results of a recent meta-analysis of 28 trials showed an increased two-year mortality in the group of patients treated with paclitaxel-coated balloons. Based on these results, and after analysis of follow-up data from the trials that led to the approval of these products, a Food and Drug Administration (FDA) panel concluded that, despite the short-term benefits with paclitaxel-based devices, safety concerns may exist for mid-term mortality risk.<br>Alternative drug candidates to paclitaxel-coated balloon catheters are the so-called limus-based analogs, which own cytostatic properties and are characterized by a wider therapeutic window. Recently, a novel balloon catheter has been CE-certified: it encapsulates sirolimus in phospholipid drug nanocarriers to improve adhesion properties of sirolimus and to provide better bioavailability. Similarly to paclitaxel-coated and uncoated devices, sirolimus-coated devices are currently approved for routine use in PAD and reimbursed in Switzerland.<br>The aim of the present trial is to compare the efficacy, as defined by a composite of clinically relevant non-subjective ´hard´ outcomes (major amputation and target lesion re-vascularization for critical limb ischemia), of sirolimus-coated vs. uncoated balloon angioplasty for peripheral artery disease in patients scheduled for infra-inguinal re-vascularization and selected based on a very limited number of inclusion criteria (all comers) aiming at maximization of external validity.
**Objectives**	**Primary Objective**<br>To evaluate whether the use of sirolimus-coated balloon catheters is non-inferior to uncoated balloon catheters in infra-inguinal angioplasty to prevent one-year major adverse limb events (MALE), including unplanned major amputation of the target limb and target lesion re-vascularization for critical limb ischemia, in a representative population of patients with PAD (\`all-comers\`).<br>If the criterion for non-inferiority is confirmed, the study will test whether sirolimus-coated catheters are superior to uncoated catheters for important secondary outcomes and for the primary outcome itself according to pre-specified criteria for hierarchical analysis.<br>**Secondary Objective**<br>To assess the safety of uncoated balloon catheters and test whether their efficacy is maintained across important subgroups of patients (heterogeneity of treatment effect analysis).
**Outcomes:**	**Primary Outcome**<br>The primary efficacy outcome is a composite of two major adverse limb events (MALE)<br>(i) unplanned major amputation of the target limb, and<br>(ii) endovascular or surgical target lesion re-vascularization for critical limb ischemia<br>and will be assessed within one year of enrolment for the primary analysis.<br><br>**Secondary Outcomes**

	• A composite of unplanned (major or minor) index limb amputations or any target lesion re-vascularization within 365 days of enrolment (tested as part of the hierarchical analysis if the criterion for non-inferiority is fulfilled).<br>• Clinical improvement by >1 Rutherford category through 180 Days;<br>• Any target lesion re-vascularization performed within 365 days after enrolment;<br>• Unplanned target lesion re-vascularization for non-critical limb ischemia performed within 365 days;<br>• Unplanned target lesion re-vascularization for critical limb ischemia within 365 days after enrolment;<br>• Unplanned target limb re-vascularization within 365 days after enrolment;<br>• Unplanned minor amputation at target limb performed within 365 days after enrolment;<br>• Unplanned major amputation at target limb performed within 365 days after enrolment;<br>• Any unplanned amputation.<br>**Safety Outcomes**<br>• Death from all causes within 30 days, 180 days, 365 days, 2 years, and 5 years.<br>• Serious adverse events (SAEs) during initial hospitalization, within 180 days, and within 365 days.<br>• Serious adverse device related events (SADE) during initial hospitalization.<br>• A composite of all-cause death and MALE within 30 days.
**Study design:**	Multicentric, randomized controlled (1:1), open-label, non-inferiority trial with hierarchical analysis for superiority.
**Inclusion / Exclusion criteria:**	**Inclusion Criteria**<br>• Age ≥ 18 years.<br>• Patients requiring endovascular angioplasty for PAD located below the inguinal ligament (predefined clinical and angiographic criteria are listed below).<br>• Written informed consent obtained from participant or legal guardian prior to randomization; in patients requiring emergency interventional treatment who are temporarily not capable of providing informed consent, consent will be subsequently obtained after the procedure if strict conditions apply. These include the assessment of the presumed will and patient decree, and requires the allocation of an independent physician.<br>**Exclusion Criteria**<br>• Pregnancy, breastfeeding, or planned pregnancy within the trial period or women of childbearing potential not using an adequate method of contraception.<br>• Patients with known intolerance or allergy to sirolimus.<br>• Participation in this or other clinical trials during the previous 3 months.
**Measurements and procedures**	**1. Clinical screening phase and baseline characteristics**<br>- Stratification (elective or rescue intervention)<br>- Fontaine and Rutherford classification

	- Age, sex, weight, height, blood pressure, occlusion pressures<br>- Risk factors (diabetes mellitus, arterial hypertension, dyslipidemia, immunosuppressive therapy, smoking)<br>- Medical history (autoimmune disease, dementia, kidney transplantation, cancer, osteomyelitis, intermediate or intensive care treatment during current hospital stay at baseline, ischemic heart disease, cerebrovascular disease, renal insufficiency, previous amputation)<br>- Results from routine blood tests (hemoglobin, platelet count, creatinine, eGRF, triglyceride, total cholesterol, LDL)<br>- Planned amputation prior to randomization (major or minor)<br>- Previous surgical and interventional procedures<br><br>**2a. Angiographic screening phase and definition of target lesion**<br><br>For the purpose of this study, the target lesion is defined as the main lesion considered responsible for the patient's signs and symptoms, fulfilling the following angiographic criteria:<br>1) stenosis (lumen compromise ≥50%) in at least a single plane of the femoro-popliteal arterial segment including the femoral, deep femoral and or popliteal artery or a femoropopliteal bypass, or<br>2) stenosis (lumen compromise ≥50%) of the below-the-knee arterial segment including the tibioperoneal trunk and/or the anterior tibial, peroneal, or posterior tibial artery, or a below the knee bypass.<br><br>The treating physician will determine the localization of the target lesion after diagnostic angiography and before enrolment of the patient in the study.<br><br>**2b. Randomization and allocation to treatment arm**<br>Randomization will be performed to define the treatment strategy (drug-coated versus uncoated). Concomitant in-/outflow disease (arterial obstruction with lumen compromise >50%) may be treated upon discretion of the interventionist. The study does not mandate treatment of in- and outflow lesions but the physician may treat those lesions per discretion. The target lesion has to be treated according to group assignment (randomization), whereas in- and outflow lesions are treated with uncoated devices.<br><br>**The following parameters will be recorded after the intervention**<br><u>Type of target lesion</u><br>- Stenosis (50-99% lumen compromise) versus occlusion<br>- If lesion lays within peripheral bypass, type of bypass (prosthetic, vein, composite)<br>- Total target lesion length<br>- Minimum diameter of target lesion<br>- De-novo lesion<br>- Re-stenosis (PTA or in-stent)<br>- In-stent occlusion<br><br><u>Interventional report</u><br>Interventional details on location and type of treatment (balloon angioplasty, provisional stent) in target lesion and target limb

	including details on location and type of treatment of concomitant inflow and outflow disease<br><br><u>Crural outflow at target limb at the end of the procedure</u><br>The quality of outflow vessels (tibioperoneal trunk, anterior tibial artery, posterior tibial artery and peroneal artery) will be categorized according with the degree of occlusion, as follows: occluded (100% lumen compromise), stenotic (>50% lumen stenosis) or patent (<50% lumen stenosis) according to the final angiogram.<br><br>**3. Discharge**<br>- Evaluation of occurrence of primary efficacy, primary safety and secondary outcome during hospitalization<br>- Evaluation of complications during or after intervention (early occlusion in or out of target lesion, hematoma, pseudoaneurysm, AV-fistula, bleeding, allergic reactions, acute coronary syndrome, stroke, compartment syndrome, renal failure)<br>- Planned antithrombotic management at discharge<br>- Planned cholesterol medication at discharge<br>- Adverse events<br><br>**4. Follow-up visits (Day 180 ± 30 and Day 365 ± 21)**<br>There will be no additional scheduled physical office visit outside the routine follow-up schedule. However, most patients are followed-up 1-6 months and 12 months after angioplasty. Patients, relatives, and the treating (referring) physicians will be instructed to refer to the hub hospital in case of signs or symptoms of critical limb ischemia. Physicians of patients that were referred to our hospital for treatment only (no visits in outpatient clinic) will be prospectively contacted and interviewed to obtain the mandatory outcome information (MALE, TLR, death) and relevant doctor's letter. In case of missing data, doctors or relatives of patients might be contacted directly by telephone.<br><br>**The following parameters will be recorded:**<br>- Occurrence of primary efficacy, primary safety and secondary outcome since discharge/last visit<br>- Occurrence of bleeding complication since discharge/last visit<br>- Occurrence of other complications since discharge/last visit (new untreated >50% stenosis in target lesion, new untreated occlusion in target lesion, stroke, myocardial infarction, newly diagnosed cancer)<br>- Current antithrombotic therapy<br>- Current cholesterol medication<br>- Adverse events<br><br>**5. Follow-up (Year 2 and Year 5)**<br>- Mortality information from a vital registration database, as well as online charts will be reviewed for the occurrence of the primary safety outcome and, if possible, the cause of death (ICD-10).
**Sirolimus-coated group (treatment group)**	Upon angiographic determination of the target lesion, primary plain old balloon angioplasty (POBA, uncoated) may be performed for vessel preparation (pre-dilatation) using a balloon diameter corresponding to the reference vessel diameter.

	Patients will receive target lesion treatment with the Magic Touch PTA sirolimus coated balloon (Concept Medical B.V., Hoevelaken, The Netherlands). All balloons will be inflated for a duration of 120 seconds at nominal pressure. Nominal pressure is defined as the inflation pressure required to reach the device-specific diameter.<br><br>Provisional stent placement will be performed at the discretion of the interventionist.<br><br><u>For plain old balloon angioplasty:</u><br>Any available CE-certified uncoated balloon catheter with application in peripheral artery disease approved for in-patient use in Switzerland.<br><br><u>For provisional stent placement</u>:<br>Any available CE-certified uncoated vascular stent with application in peripheral arterial disease approved for inpatient use in Switzerland.
**Uncoated-group / control group**	Upon angiographic determination of the target lesion, plain old balloon angioplasty (POBA, uncoated) will be performed using a balloon diameter corresponding to the reference vessel diameter. All balloons will be inflated for a duration of 120 seconds at nominal pressure. Nominal pressure is defined as the inflation pressure required to reach the device-specific diameter.<br><br>Provisional stent placement will be performed at the discretion of the interventionist.<br><br><u>For plain old balloon angioplasty:</u><br>Any available CE-certified uncoated balloon catheter with application in peripheral artery disease approved for in-patient use in Switzerland.<br><br><u>For provisional stent placement</u>:<br>Any available CE-certified uncoated vascular stent with application in peripheral arterial disease approved for inpatient use in Switzerland.
**Number of Participants with Rationale:**	Assuming a 10% event rate (MALE) within 12 months of enrollment in both the control and intervention group, and a non-inferiority margin of 5% expressed as absolute risk difference, a total of 1132 patients (566 patients per treatment group) allow to show non-inferiority of the intervention group with a power of 80% and a type I error rate of $\alpha=2.5\%$ one-sided. Assuming a drop-out rate of approximately 5%, a total of 1200 patients will be randomized in the study.<br>The sample size calculation is based on previous studies reporting MALE rates in PAD patients after re-vascularization procedures and personal experience / direct observation at the study center.
**Study Duration:**	The Clinic of Angiology of the University Hospital of Zurich alone currently performs approximately 800 peripheral interventions per year. The Clinic of Angiology of the Fribourg Hospital performs approximately 250 peripheral interventions per year. Therefore, assuming that more than 80% of the eligible patients will be enrolled in the study, the patient recruitment is expected to last no longer than 30 months for a total duration of the study of 54 months, accounting for the enrolment period followed by a 12-

	month follow-up for clinical outcomes and 5-year follow-up for mortality.
**Study Schedule:**	First-Patient-In: Q4 2020<br>Last-Patient-Out: Q2 2028
**Investigators**	PD Dr. med. Stefano Barco, PhD<br>Co-Principal Investigator (Co-PI)<br>University Hospital Zürich<br>Clinic of Angiology<br>Rämistrasse 100<br>8091 Zürich (Switzerland)<br>E-Mail: Stefano.Barco@usz.ch<br><br>Dr. med. Tim Sebastian<br>University Hospital Zürich<br>Clinic of Angiology<br>Rämistrasse 100<br>8091 Zürich (Switzerland)<br>E-Mail: Tim.Sebastian@usz.ch<br><br>Dr. med. Alexandru Grigorean<br>University Hospital Zürich<br>Clinic of Angiology<br>Rämistrasse 100<br>8091 Zürich (Switzerland)<br>E-Mail: alexandru.grigorean@usz.ch<br><br>Dr. med. Mario Münger<br>University Hospital Zürich<br>Clinic of Angiology<br>Rämistrasse 100<br>8091 Zürich (Switzerland)<br>E-Mail: mario.muenger@usz.ch<br><br>PD Dr. med. Erik Holy<br>University Hospital Zürich<br>Clinic of Angiology<br>Rämistrasse 100<br>8091 Zürich (Switzerland)<br>E-Mail: erik.holy@usz.ch<br><br>Dr. med. Davide Voci<br>University Hospital Zürich<br>Clinic of Angiology<br>Rämistrasse 100<br>8091 Zürich (Switzerland)<br>E-Mail: davide.voci@usz.ch<br><br>PD Dr. med. Rolf Engelberger<br>Department of Angiology, HFR Freiburg<br>Kantonsspital, Chemin des Pensionnats<br>1708, Freiburg (Switzerland)<br>E-Mail: Rolf.Engelberger@h-fr.ch<br><br>Dr. med. Daniel Périard<br>Department of Angiology, HFR Freiburg<br>Kantonsspital, Chemin des Pensionnats<br>1708, Freiburg (Switzerland)

	E-Mail: Daniel.Periard@h-fr.ch
**Data safety monitoring officer**	Prof. Dr. med. Marc Righini<br>Division of Angiology and Hemostasis, Faculty of Medicine<br>Geneva University Hospitals<br>Rue Gabrielle-Perret-Gentil 4<br>1205 Geneva, Switzerland<br>E-Mail: Marc.Righini@hcuge.ch
**Biostatistician**	Prof. Dr. Ulrike Held<br>Department of Biostatistics<br>Epidemiology, Biostatistics and Prevention Institute<br>University of Zurich<br>Hirschengraben 84<br>8001 Zurich<br>Switzerland<br>Phone +41 44 634 45 39<br>E-Mail: Ulrike.Held@uzh.ch
**Centers**	University Hospital Zurich<br>Clinic of Angiology<br>Rämistrasse 100<br>8091 Zürich (Switzerland)<br><br>Department of Angiology, HFR Freiburg<br>Kantonsspital, Chemin des Pensionnats<br>1708, Freiburg (Switzerland)
**Statistical Considerations**	**Primary analysis**<br>In the primary analysis of this trial, the absolute risk difference for MALE at 12 months follow-up between treatment groups will be estimated, together with its 2-sided 95% confidence interval. The trial statistician will perform the analyses after termination of the trial.<br><br>If the null hypothesis concerning the primary objective is rejected (and the primary objective, non-inferiority, is thus established), further confirmatory statistical tests on secondary endpoints will be performed using a prespecified hierarchical order based on the clinical importance of the considered outcomes:<br>1) superiority for the composite of unplanned (major or minor) index-limb amputations or any target lesion re-vascularization within 365 days after enrolment (α=2.5% one-sided);<br>2) superiority for MALE within 365 days after enrolment (α=2.5% one-sided).<br><br>**Secondary Analyses**<br>Secondary analyses include time-to-event analyses of MALE, estimation of hazard rates in both treatment groups and estimation of a hazard ratio with 95% confidence interval. Cox proportional hazards model will be applied.<br>Subgroup analyses to study the heterogeneity of treatment effects will be conducted, i.e. in patients categorized by sex, and elective vs. critical rescue re-vascularization.<br>**Interim analysis**

	An interim analysis for efficacy is planned after 50% of the patients in both treatment groups have completed 12 months follow-up. If the overall MALE event rate at interim is ≤10%, the trial will continue without any adaptations. If the overall MALE event rate is >10%, event rates in both treatment groups will be estimated in unblinded fashion, and the risk difference with confidence interval will be reported. To preserve the overall type I error for one interim analysis and the final analysis, the significance level α of 0.05 (2-sided) is spent using the Lan-DeMets spending function. At interim analysis, a 99.95% confidence interval is calculated, at final analysis, a 95.05% confidence interval is calculated. Based on the results at interim, Bayesian predictive probabilities for successful termination of the trial will be calculated.
**Statement**	This study will be conducted in compliance with the investigation plan, the current version of the Declaration of Helsinki, the ISO 14155 as well as all national legal and regulatory requirements.

ABBREVIATIONS

AE	Adverse Event
BMS	Bare Metal Stents
CA	Competent Authority (e.g. Swissmedic)
CE	Certification Europe
CLI	Critical Limb Ischemia
ClinO	Clinical Trials Ordinance
CRF	Case Report Form
eCRF	Electronic Case Report Form
CTCAE	Common terminology criteria for adverse events
DCB	Drug-coated Balloon(s)
DES	Drug-eluting Stent(s)
FDA	Food and Drug Administration
GRAS	Generally Recognized As Safe
H0	Null hypothesis
H1	Alternative hypothesis
IC	Intermittent claudication
IMD	Investigational Medical Device
ISF	Investigator Site File
ITT	Intention to Treat
LHR	Law on human research
MALE	Major Adverse Limb Events
mTOR	Mammalian Target of Rapamycin
PAD	Peripheral Arterial Disease
PI	Principal Investigator
PMA	Premarket Approval
POBA	Plain-old balloon angioplasty
PTA	Percutaneous transluminal angioplasty
RCT	Randomized-controlled Trial(s)
SAE	Serious Adverse Event
SDV	Source Data Verification
SNCTP	Swiss National Clinical Trial Portal
SOP	Standard Operating Procedure
TLR	Target lesion re-vascularization
TMF	Trial Master File

SUMMARY OF THE REVISION HISTORY IN CASE OF AMENDMENTS

Version Nr, Version Date	Chapter	Description of change	Reason for the change
Version 2.0 of 06.10.2021	1,<br>10.1	Assessment of vital status instead of vital signs	Prolongation of the trial FUP
Version 2.0 of 06.10.2021	1,<br>6.3,<br>10.1, 10.2.3.1,<br>10.2.4, 10.3.2	Addition of V7 (5-year FU)	Prolongation of the trial FUP
Version 2.0 of 06.10.2021	1,<br>6.2, 6.3,<br>10.1, 10.2.1,<br>10.2.2, 10.3.4	Adaptation of time frame V4	Precisely defined time frame
Version 2.0 of 06.10.2021	2.2,<br>10.2.1	New Investigators listed	New Study center, Corrections
Version 2.0 of 06.10.2021	3.1,<br>3.3	Category change from A to A1	MDR requirements
Version 2.0 of 06.10.2021	3.2,<br>3.8	Adaptation of study termination reporting	Corrections
Version 2.0 of 06.10.2021	3.9	Reporting of non-substantial amendments	Corrections
Version 2.0 of 06.10.2021	7.1	Multicenter	New Study center
Version 2.0 of 06.10.2021	7.2	Adjustment of study duration and study schedule	New Study center and effective dates
Version 2.0 of 06.10.2021	9.8,<br>14.3	Deletion of device accountability	Not needed
Version 2.0 of 06.10.2021	11.1, 11.2,<br>11.3, 11.4	Safety reporting adapted	MDR requirements
Version 2.0 of 06.10.2021	15	Addition of clinicaltrials.gov	Findings will be reported in clinicaltrials.gov

1 STUDY SCHEDULE

Table 1: Flow chart

**Procedures**	**Clinical screening**	**Angiography screening, randomization, and treatment (baseline)**		**Clinical follow-up and assessment of the vital status**		
**Visit**	**V1**	**V2**	**V3**	**V4**	**V5**	**V6-V7**
**Time**	**Day -90 to 0**	**Day 1**	**Discharge from hospital**	**Day 180 ± 30**	**Day 365 ± 21[2]**	**Year 2-Year 5[2]**
Patient Information and Informed Consent for screening and participation	X					
Ultrasonography or evaluation of prior echographic findings	(X)	(X)				
Staging (Fontaine, Rutherford)	X			(X)[1]	(X)[1]	
Collection of personal information (demographics, risk factors, medical history, concomitant medication)	X					
Decision about planned amputation	X					
Vital signs	X	X	X			
Vital status				(X)[1]	(X)[1]	(X)[1]
Laboratory tests (routine)	X	(X)				
Evaluation of information concerning prior endovascular/surgical procedures	X					
Verification of the eligibility criteria	X	X				
Definition of target lesion		X				
Randomization and allocation		X				
Intervention		X				
Primary and secondary efficacy outcomes assessment			X	X[1]	X[1]	
Primary safety outcome assessment (death)		X	X	X[1]	X[1]	X
Assessment of (S)AEs and secondary safety outcomes		X	X	X	X	
Assessment of antithrombotic and lipid lowering medication			X	X	X	
**Type of contact**	In-hospital	In-hospital	In-hospital	In-hospital[1] or teleph.; (national vital registry)	In-hospital[1] or teleph.; (national vital registry)	National vital registry; (teleph.)

[1]Follow-up visits will be performed during routine visits at our outpatient clinics (hub and satellites). Patients who are not routinely follow-up in our outpatient clinics will be interviewed by telephone, or information will be gathered by contacting relatives. In addition, we may collect follow-up reports from external vascular specialists and the general practitioners. [2]Since vital registration databases may not be updated on a daily basis, information concerning the vital status will be collected at Day 365 +/- 21 days, Year 2, or Year 5; the vital status reported in the eCRF will refer to that on Year 1, 2, and 5 will be recorded.

2 STUDY ADMINISTRATIVE STRUCTURE

2.1 Sponsor-Investigator

Prof. Dr. med Nils Kucher
University Zurich
University Hospital Zurich
Clinic of Angiology
Rämistrasse 100
CH 8091 Zürich
Switzerland
Phone: +41 44 255 4082
Fax: +41 44 255 45 10
E-Mail: nils.kucher@usz.ch

2.2 Investigators

PD Dr. (I) Stefano Barco, PhD
Co-Principal Investigator (Co-PI)
University Hospital Zürich
Clinic of Angiology
Rämistrasse 100
8091 Zürich (Switzerland)
E-Mail: Stefano.Barco@usz.ch

Dr. med. Tim Sebastian
University Hospital Zürich
Clinic of Angiology
Rämistrasse 100
8091 Zürich (Switzerland)
E-Mail: Tim.Sebastian@usz.ch

Dr. med. Alexandru Grigorean
University Hospital Zürich
Clinic of Angiology
Rämistrasse 100
8091 Zürich (Switzerland)
E-Mail: alexandru.grigorean@usz.ch

Dr. med. Mario Münger
University Hospital Zürich
Clinic of Angiology
Rämistrasse 100
8091 Zürich (Switzerland)
E-Mail: mario.muenger@usz.ch

PD Dr. med. Erik Holy
University Hospital Zürich
Clinic of Angiology
Rämistrasse 100
8091 Zürich (Switzerland)
E-Mail: erik.holy@usz.ch

Dr. med. Davide Voci
University Hospital Zürich
Clinic of Angiology
Rämistrasse 100
8091 Zürich (Switzerland)
E-Mail: davide.voci@usz.ch

Dr. med. Daniel Périard
Department of Angiology, HFR Freiburg
Kantonsspital, Chemin des Pensionnats
1708, Freiburg (Switzerland)
E-Mail: Daniel.Periard@h-fr.ch

PD Dr. med. Rolf Engelberger
Department of Angiology, HFR Freiburg
Kantonsspital, Chemin des Pensionnats
1708, Freiburg (Switzerland)
E-Mail: Rolf.Engelberger@h-fr.ch

2.3 Statistician ("Biostatistician")

Prof. Dr. rer. nat. Ulrike Held
Dept. of Biostatistics - Epidemiology, Biostatistics and Prevention Institute
University of Zurich
Hirschengraben 84
8001 Zurich
Switzerland
Phone +41 44 634 45 39
E-Mail: ulrike.held@uzh.ch

2.4 Monitoring Institution

Clinical Trials Center - Monitoring
Universitätsspital Zürich
Rämistrasse 100 / MOU2
8091 Zürich

2.5 Data Safety Monitoring Officer

Prof. Dr. med. Marc Righini
Division of Angiology and Hemostasis, Faculty of Medicine
Geneva University Hospitals
Rue Gabrielle-Perret-Gentil 4
1205 Geneva, Switzerland
E-Mail: Marc.Righini@hcuge.ch

2.6 Study Coordinator

Dr. sc. nat. Rebecca Spescha
University Hospital Zürich
Clinic of Angiology
Rämistrasse 100
8091 Zürich (Switzerland)
E-Mail: Rebecca.spescha@usz.ch

3 ETHICAL AND REGULATORY ASPECTS

Before the start of the present study, the investigation plan, the proposed participant information and consent forms, as well as other study-specific documents, will be submitted to a properly constituted Competent Ethics Committee in agreement with local legal requirements, for formal approval. Any further amendment to the investigation plan will be submitted to the Competent Ethics Committee and taken into place upon approval.

The decision of the Competent Ethics Committee concerning the conduct of the study will be made in writing to the Sponsor-Investigator before commencement of the study, which can begin only after the approval has been received from all the required authorities. Any additional requirements imposed by the authorities will be implemented.

This study will be conducted in compliance with the protocol and all other applicable regulatory requirements including the archiving of essential documents.

3.1 Study Registration

Upon approval of the Ethics Committee, the study protocol will be registered in the international Web-based resource ClinicalTrials.gov (available at www.clinicaltrials.gov; ID NCT04238546), which provides patients, their family members, health care professionals, researchers, and the public with easy access to information on publicly and privately supported clinical studies on a wide range of diseases and conditions Categorization of Study

This clinical trial refers to a medical device belonging to the medical device category A1. The interventional medical device (MagicTouch PTA sirolimus coated balloon, Concept Medical B.V., Hoevelaken, The Netherlands) and all medical devices used as the comparator (uncoated balloon catheters) are CE-marked products already in use in clinical routine, also at the study center (Clinic of Angiology of the University Hospital of Zurich), for a spectrum of clinical conditions including that object of the study.

3.2 Competent Ethics Committee

Approval from the appropriate constituted Competent Ethics Committee is sought before the start of the clinical trial. The reporting duties and allowed time frame will be respected. No substantial changes are made to the investigation plan without provisional Sponsor and competent Ethical Committee approval, except where necessary to eliminate apparent immediate hazards to study participants.

Premature study termination or interruption will be reported within 15 days. The regular study termination will be reported to the Competent Ethics Committee within 15 days of study end; the final study report will be submitted within one year of study end or three months of premature study termination.

3.3 Competent authority (CA)

No approval from Swissmedic is deemed necessary for clinical trials investigating medical devices belonging to the category A1 (previously category A).

3.4 Ethical Conduct of the Study

The study will be carried out in accordance with principles enunciated in the European Directive on medical devices 93/42/EEC and the ISO Norm 14155 and ISO 14971, the Swiss Law and Swiss regulatory authority's requirements. Competent Ethics Committee will receive annual safety and interim reports and be informed about study stop/end in agreement with local requirements.

3.4.1 Compliance with the EN ISO 14155 standard

For clinical trials involving a particularly low risk of adverse events, certain deviations are possible, particularly for post-market trials. The protection of the participants and data quality and security will not be affected by such deviations. All deviations will be disclosed in the clinical investigation plan (CIP) of the clinical trial. A separate, dedicated section in the CIP is recommended for deviations (e.g. "Compliance with the ISO 14155 standard"). The deviation will be described and the absence of effects of the deviation on the protection of the participants and data quality and security must be justified.

3.5 Declaration of Interest

This investigator-initiated study has received an unrestricted grant from Concept Medical B.V. (Hoevelaken, the Netherlands). Concept Medical B.V. did not contribute to the preparation of the present study protocol.

3.6 Patient Information and Informed Consent

The patient information leaflet describing the purpose and design of the study and the informed consent form are submitted together with the study protocol for review and approval from the Competent Ethics Committee. The documents are available in German, French, Italian, and English language. For any other language, a certified translator will be assisting on a case by case basis. The certified translator will confirm in writing the informed consent discussion. Further assistance by certified translators beyond the baseline visit are not intended. There are four scenarios in regards of the process of obtaining informed consent (Figure 1).

Scenario 1 – Patients scheduled for elective procedures and able to consent

The written consent will be collected using the Informed Consent Form 1 before the participant would undergo any study procedure. Before enrolment, the study investigators will explain to each participant the nature of the study, its purposes, the procedures involved, the expected duration, the potential risks and benefits, and any discomfort or deviation from routine management it may entail. Each participant will be informed that the participation in the study is voluntary and that he/she may withdraw from the study at any time: the withdrawal of consent will not affect his/her subsequent medical treatment. The participant will be informed that i) his/her medical records may be examined by authorized individuals other than their treating physicians; ii) he/she will be contacted telephonically for assessing the vital and health status; iii) close relatives might be contacted over phone for assessment of his/her personal vital or health status; iv) national vital registry data will be requested to verify his/her personal vital status in case he/she would miss an in-hospital visit and cannot be reached telephonically. The study participants will be provided with a patient information leaflet and an informed consent form describing this study and reporting enough information for participants in order to make an informed decision about their participation. The participant will read and have enough time to consider the statement before signing and dating the informed consent form, and he/she will be given a copy of the signed document. The consent form will be signed and dated by the investigator, or his/her designee, and retained as part of the study records.

Scenario 2 – Patients with legal guardians scheduled for elective procedures

The written consent of a vulnerable patient with legal guardians will be obtained before that participant undergoes any study procedure. The legal guardians will be fully informed using the Informed Consent Form 2 for Legal Representatives. The legal representative will act in the best patient`s interest and consult close relatives to make an appraisal of the presumed will. Before enrolment, the study investigators will explain to the legal representative the nature of the study, its purposes, the procedures involved, the expected duration, the potential risks and benefits, and any discomfort it may entail. The legal representative will be informed before screening that the participation of his ward in the study is voluntary and that he/she can withdraw his ward from the study at any time: the withdrawal of consent will not affect the ward's subsequent medical treatment. The legal representative will be informed that i) the ward's medical records may be examined by authorized individuals other than their treating physician; ii) the ward might be contacted over the phone for assessment of vital and health status; iii) close relatives of the ward

including the legal representative might be contacted over phone for assessment of his/her personal vital or health status; iv) national vital registry data will be assessed for the ward's personal vital status in case he/she would miss an in-hospital visit and cannot be reached telephonically. The legal guardian will be provided with a patient information leaflet and an informed consent form describing this study and reporting enough information in order to make an informed decision about their ward's participation and clinical follow-up. The legal guard will read and consider the statement before signing and dating the informed consent form and should be given a copy of the signed document. The consent form will be signed and dated by the investigator, or his designee, and retained as part of the study records.

Scenario 3 - Patients with legal guardians requiring rescue revascularization

Without holding a formal consent of a participant with legal guardians, he/she can be enrolled under reserve in case of an emergency, if all of the following conditions apply:

- Legal representative is not available, and rescue revascularization cannot further be delayed;
- Assessment of the presumed will by consulting the patient degree if available, or contacting a designated confidant or a relative.
- Allocation of an independent physician not associated with the study, which will preserve the interests of the unconscious individual and guard his/her medical care.
- The independent physician needs to be consulted prior to any study-specific intervention. He/she must be educated of the study and its procedures and risks. Agreement must be provided by signing the Patient Informed Consent Form 3 for Independent Physicians. The independent physician will have immediate access to the study protocol. If, in exceptional cases, the independent physician was not consulted prior to enrolment, this needs to be explained and documented

The formal consent, using the approved Consent Form 4, must be subsequently provided as soon as the legal representative is available. The legal guardians will be fully informed using the Informed Consent Form 4 for Legal Representatives. The legal representative must act in the best interest of the patient and, especially if not related, consult close relatives to make an appraisal of the presumed will. The study investigators will explain to the legal representative that the patient has been enrolled under reserve as a participant in this study. He/she will explain the nature of the study, its purposes, the procedures involved, the expected duration, the potential risks and benefits and any discomfort it may entail. The legal representative will be informed that the participation of his ward in the study is voluntary and that he/she can be immediately withdrawn his ward from any further study procedures: the withdrawal of consent will not affect the ward's subsequent medical treatment. The legal representative will be informed that i) the ward's revascularization procedure has been already performed and that the decision whether to use Sirolimus-coated or uncoated balloon catheters was based on randomization (1:1); ii) the ward's medical records may be examined by authorized individuals other than their treating physician; ii) the ward might be contacted over the phone for assessment of vital and health status; iii) close relatives of the ward including the legal representative might be contacted over phone for assessment of his/her personal vital or health status; iv) national vital registry data will be assessed for the ward's personal vital status in case he/she would miss an in-hospital visit and cannot be reached telephonically. The legal guardian will be provided with a patient information leaflet and an informed consent form in order to make an informed decision about their ward's participation and clinical follow-up. The legal guard will read and consider the statement before signing and dating the informed consent form and should be given a copy of the signed document. The consent form will also be signed and dated by the investigator, or his designee, and retained as part of the study records. The lack of a formal written consent represents a major violation of the protocol causing the exclusion of the patient from the study and stopping of any subsequent study procedure.

Scenario 4 – Patients temporarily not able to consent requiring rescue revascularization

Without holding a formal consent of an unconscious participant temporarily not discerning, he/she can be enrolled under reserve in case of an emergency, if all of the following conditions apply:

- Assessment of the presumed will by consulting the patient decree if available, or contacting a designated confidant or a relative.

- Allocation of an independent physician not associated with the study, which will preserve the interests of the unconscious individual and guard his/her medical care.
- The independent physician needs to be consulted prior to any study-specific intervention. He/she must be educated of the study and its procedures and risks. Agreement must be provided by signing the Patient Informed Consent Form 3 for Independent Physicians. The independent physician will have immediate access to the study protocol. If, in exceptional cases, the independent physician was not consulted prior to enrolment, this needs to be explained and documented.

The formal consent, using the approved Consent Form 1, must be subsequently provided as soon as the patient is conscious and accountable. This is done analogously to the procedure described in Scenario 1. If the patients regains power of judgement and does not wish to participate in the trial, he immediately drops out of the study. In this case, already collected data can be utilized only with permission of the patient. If the patient remains unaccountable or dies, the formal consent must be subsequently provided by a legal representative analogously to the procedure described in Scenario 3. The lack of a formal written consent represents a major violation of the protocol causing the exclusion of the patient from the study and stopping of any subsequent study procedure.

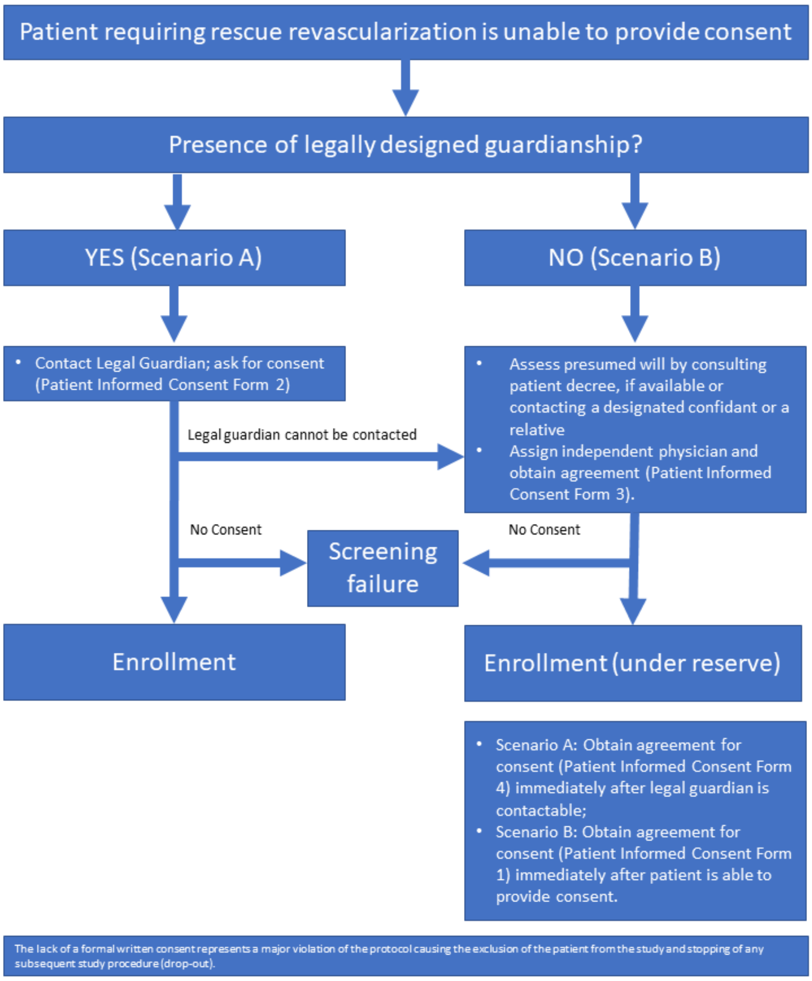

Figure 1: Flow chart for process of obtaining informed consent in patients requiring rescue revascularization who are unable to provide consent.

3.7 Participant Privacy and Confidentiality

The investigators affirm and uphold the principle of the participant's right to privacy and that they will comply with applicable privacy laws. Anonymity of the participants will be guaranteed when presenting the data at scientific meetings or publishing them in scientific journals.

Individual subject medical information obtained as a result of this study is considered confidential and disclosure to third parties is prohibited. Subject confidentiality will be further ensured by utilizing subject identification code numbers to correspond to treatment data in the computer files.

Such a medical information may be given to the participant's personal physician or to other appropriate medical personnel responsible for the participant's welfare, if the patient has given his/her written consent to do so.

For data verification purposes, authorized representatives of the Sponsor-Investigator, a competent authority for inspection, or an ethics committee may require direct access to parts of the medical records relevant to the study, including participants' medical history.

3.8 Early Termination of the Study

The Sponsor-Investigator may decide to terminate the study prematurely if any of the following circumstances occur:

- Insufficient participant recruitment, e.g. recruitment of less than 50% of the planned number of enrolled patients after one year.
- Substantial changes in clinical practice or publication of the results of new trials that make the continuation of this trial unwise.
- Safety concerns for MALE at the interim analysis, which will be conducted after the completion of 12-month follow-up of the first 600 patients, corresponding to approximately 53% of the total planned sample size (predefined rules for interim analysis).
- Safety concerns for death rate or SAE between treatment groups (continuous safety evaluation will be after multiples of 150 patients being enrolled).

In case of early termination, a closure letter will be prepared within 3 months (Art. 36 KlinV-Mep). In case of early termination due to safety reasons, the Clinical Event Committee will be informed within 24 hours (Art. 37 KlinV-Mep).

3.9 Investigation Plan Amendments

Should any change be required to the signed final protocol, a protocol amendment will be required. The amendment must be signed at least by the investigator-sponsor, and, if necessary, by the statistician.

<u>Substantial amendment</u>

If an amendment is substantial and likely to have an impact on the safety of the clinical trial patients or to change the interpretation of the scientific documents in support of the conduct of the clinical trial, or if it is otherwise considered relevant for the conduction of the study, the sponsor or an authorized representative will notify the ethics committee.

Should the opinion of the ethics committee will be favorable and the competent authorities have raised no grounds for non-acceptance of the above-mentioned substantial amendment, the sponsor will proceed to conduct the clinical trial following the amended protocol.

If the opinion of the Competent Ethics Committee is unfavorable, the sponsor will not implement the amendment in the protocol. The sponsor will either adapt the amendment based on the comments received or withdraw it.

Significant changes to be authorized by the ethics committee are the following:

- changes affecting the participants' safety and health, or their rights and obligations;
- changes to the protocol, and in particular changes based on new scientific knowledge which concern the trial design, the method of investigation, the endpoints or the form of statistical analysis;
- a change of sponsor, coordinating investigator or investigator responsible at a trial site.

Non-substantial amendments

Protocol amendments only for logistical or administrative changes may be implemented immediately; the Competent Ethic Committee will be informed by the sponsor accordingly.

All non-substantial amendments are communicated to the CEC together with the Annual Safety Report (ASR) (Art. 15 ClinO-MD).

Urgent safety measures

If any new event related to the conduct of the trial or the development of the IMD occurs that is likely to affect the safety of the patient, the sponsor-investigator will take appropriate urgent safety measures to protect the patients against any immediate hazard. The sponsor or an authorized representative, respectively, will inform the competent authorities of this new event and the measures taken immediately and will ensure that the Competent Ethics Committee is notified at the same time.

4 INTRODUCTION

4.1 Background and Rationale

Peripheral artery disease (PAD) is a progressive atherosclerotic disease with symptoms ranging from intermittent claudication (IC) to critical limb ischemia (CLI). Common cardiovascular risk factors play a major role in the development of PAD: these include, among others, age, smoking, obesity, dyslipidemia, and diabetes mellitus. The prevalence of PAD in the general population is rapidly growing and PAD continues to be a serious public health problem, especially in low- and middle-income societies.[1]

PAD is associated with impaired quality of life, increased risk of hospitalization, need for limb amputation, and a high rate of other severe complications and death.[2] In addition to optimal medical therapy, endovascular revascularization treatment represents the primary therapeutic approach to restore blood flow in symptomatic PAD patients.
The majority of symptomatic PAD patients present with atherosclerotic lesions located in the femoro-popliteal arteries. In these patients, endovascular therapy is the primary choice in case the stenosis/occlusions involve less than 25 cm of the vessel. If the lesions are longer than 25 cm, endovascular therapy still represents a good therapeutic option at experienced centers, as it is characterized by minimal rate of complications and head-to-head trials proving a superiority of surgical therapy (i.e. autologous vein bypass) are not available to this day.[2]
A minority of symptomatic PAD patients would present with infra-popliteal (distal) lesions. The endovascular treatment of below-the-knee PAD is challenging due to complex lesion patterns, which include multi-vessel atherosclerosis and are characterized by a lesion morphology with long calcified stenosis or occlusion. Moreover, the small vessel diameter would limit the success rate of any re-vascularization strategy. Endovascular re-vascularization of below-the-knee arteries is therefore primarily reserved to patients with CLI in whom amputation must be prevented.

Conventional percutaneous transluminal angioplasty (PTA) with or without bare-metal stent (BMS) placement belongs to the first generation of endovascular techniques for PAD. Its major drawback is represented by the high restenosis rate, which can be primarily attributed to neo-intimal hyperplasia and elastic recoil. The majority of below the knee arteries re-occlude after angioplasty within 3 months.
Drug-coated balloons (DCB) and drug-eluting stents (DES) have been developed to prevent neo-intimal proliferation and restenosis after PTA, an objective which had been achieved by the local application of either cytostatic (e.g. paclitaxel - a cytoskeletal disruptor) or immunosuppressive (e.g. sirolimus/everolimus - both mTOR inhibitors) substances on the vessel wall. However, in contrast to coronary interventions, peripheral (infra-inguinal) interventions often involve long segments or crossings of joint lines, which are suboptimal for excessive stent use due excessive total stent length that would be required. This has resulted in the primary use of balloon catheters with provisional spot stent placement for flow-limiting dissections and acute recoil.

Currently there are four approved DCB's indicated in the United States for the treatment of peripheral artery disease: Lutonix (PMA No.: P130024), In.Pact (PMA No.: P140010), Pulsar 18 (PMA No.: P160025) and Stellarex (PMA No.: P160049). Several DCB technologies are commercially approved in Europe (CE marked) and based on paclitaxel due to the favorable level of tissue retention and kinetics, allowing for rapid absorption and long tissue residence.
Over the past decade, a few randomized controlled trials (RCT) compared the efficacy and safety of drug-coated (mainly paclitaxel-coated) devices vs. that of uncoated ones, and demonstrated a significant reduction in restenosis rates, late lumen loss, and incidence of target lesion re-vascularization.[3-9] However, the sample size of these trials was often too small to draw firm conclusions concerning major clinical outcomes. Moreover, substantial heterogeneity of the study populations and, at the same time, too restrictive eligibility criteria limited their external validity and led to difficult interpretation of the results of meta-analyses. These trials adopted as the primary outcome surrogate and subjective outcomes, such as vessel patency and target limb re-vascularization, which may be difficult to objectively adjudicate in the setting of an open-label trial, rather than ´hard´ objective clinical endpoints, such as major amputation or urgent revascularization due to critical limb ischemia. Finally, they compared new drug-coated technology vs. older-generation uncoated devices, therefore biasing the results towards less efficacy of the standard of care compared to an outdated comparator.

Although the majority of studies proved the efficacy of paclitaxel-coated balloon angioplasty, a meta-analysis of 14 trials, assessing the benefit and risk from paclitaxel-coated balloon angioplasty for the treatment of femoro-popliteal artery disease, had shown that the efficacy of paclitaxel-coated balloons differed substantially across studies and depended on treatment strategy and lesion complexity in patients with peripheral artery disease.[10] The results of a more recent meta-analysis of 28 trials showed an elevated two-year mortality among patients treated with paclitaxel-coated balloons.[11] Based on these results, and after analysis of follow-up data from the trials that led to the approval of these products, a Food and Drug Administration (FDA) panel concluded that, despite the short-term benefits with paclitaxel-based devices, safety concerns may exist for mid-term mortality risk. Therefore, studies of paclitaxel-coated devices are supposed to collect safety data with a focus on long-term mortality and efficacy.

Alternative drug candidates to paclitaxel-coated balloon catheters are the so-called limus-based analogs, which own cytostatic properties and are characterized by a wider therapeutic window. Recently, a novel balloon catheter has been CE-certified: it encapsulates sirolimus in phospholipid drug nanocarriers to improve adhesion properties of sirolimus and to provide better bioavailability.[12] The clinical study, the XTOSI study, that led to the approval of this product showed a 100% freedom from device- and procedure related mortality, and a 91% freedom from clinically-driven target lesion re-vascularization at 6 months (n=11) for patients with infra-popliteal treatment.
Some key differences exist between sirolimus-coated and paclitaxel-coated balloons. Sirolimus is a cytostatic drug with immunosuppression action. Paclitaxel is cytotoxic. Sirolimus restrains the degradation of cyclin-dependent kinase inhibitor (CKI) that plays crucial role for VSMC cycle regulation (the initial phase of the cell cycle), whereas paclitaxel impacts predominantly during the mitosis arresting cells at a stage at which they are supposed to divide, possibly triggering pro-apoptotic mechanisms. Of note, sirolimus stents have proven effective and safe in patients with coronary disease and represent a first-choice option in these patients.
Paclitaxel-coated, sirolimus-coated, and uncoated devices are currently approved for use in PAD and reimbursed in Switzerland. At the University Hospital Zurich, the choice of using a drug-coated or uncoated device is left to the treating physician. A survey of internal data indicate that the use proportion of these devices is similar.

The aim of the present trial is to compare the efficacy, as defined by a composite of clinically relevant non-subjective ´hard´ outcomes (major amputation and target lesion re-vascularization for critical limb ischemia), of sirolimus-coated vs. uncoated balloon angioplasty for peripheral artery disease in patients scheduled for infra-inguinal re-vascularization and selected based on a very limited number of inclusion criteria (all comers) aiming at maximization of external validity.

4.2 Investigational Medical Device and Indication

Name	MagicTouch PTA sirolimus drug-coated balloon catheter
Manufacturer	Concept Medical
CE-Mark	Yes
Guidewire compatibility	0.014’’, 0.018’’, 0.035’’
Balloon diameter (mm)	1.5 to 12 mm
Balloon length (mm)	20 to 200 mm
Shaft length (cm)	90 to 150 cm

Table 2: Identity of medical device

<u>Indication for use</u>

The Magic Touch PTA Sirolimus Coated Balloon Catheter is approved for percutaneous transluminal angioplasty of infrainguinal and infrapoplital lesions.

Regulatory history

- Magic Touch PTA was approved by Indian FDA in year 2018.
- Magic Touch PTA was approved by Singapore Health Authority under special access program.
- Magic Touch PTA was granted Breakthrough Designation by USFDA for below-the-knee use (August 2019)
- Magic Touch PTA granted CE certificate (October 16, 2019)

Drug coating

The coating of the Magic Touch PTA utilizes Nanolute® technology to deliver polymer-free sirolimus encapsulated in a Generally Recognized As Safe (GRAS) phospholipid excipient. The amphiphilic properties of the phospholipid allow the sirolimus to remain encapsulated and protected from degradation for a time sufficient to ensure treatment of the target lesion. The drug is delivered to the vessel wall upon contact of the expanded balloon with the arterial lumen.

Encapsulated sirolimus is sprayed onto the balloon catheter using a low volume, low pressure coating process, coating the circumferential surface of the balloon length. This single layer coating contains sirolimus and the excipient (Lipoid E 80) in a 1:1 ratio.

The use of Nanolute® technology provides for very low in-transit drug loss resulting in reduced shedding of particulate matter that can contribute to downstream embolization. This technology also allows for better drug diffusion and drug retention throughout the artery, as well as enhanced bioavailability due to deeper penetration of the drug into the artery tissue. Evidence of this was observed in an in-vivo study that was conducted to evaluate the vessel wall distribution of sirolimus in rabbits at 1 hour, 24 hours, 3 days and 7 days using DTF-labeled sirolimus coated balloons. DTAF (5-(4,6-dichlorotriazinyl) aminofluorescein)) is a reactive dye with an absorption/emission maximum of ~492 to 516 nm that has been widely used in the staining of various biological samples for fluorescent microscopy. The samples were harvested at the aforementioned time points and histologic evaluation was conducted by confocal microscopy. After 1 hour, the DTF-sirolimus was mostly confined to the treated artery's luminal surface, involving approximately 60-70% of the circumferential area. Virtually no DTF signal was seen below the level of the internal elastic lamina.

From 24 hours to 7 days, the DTF sirolimus could be found on 30-40% of the luminal surface at or below the level of the internal elastic lamina. Some diffusion of drug deeper into the medial layer was also evident by 7 days.[12] The figure below depicts the temporal penetration of the DFT-labelled sirolimus nanoparticles after balloon inflation, as assessed by confocal microscopy. The panels on the left show a diagrammatic representation and the mid and right panels the actual cross-sectional images.[12]

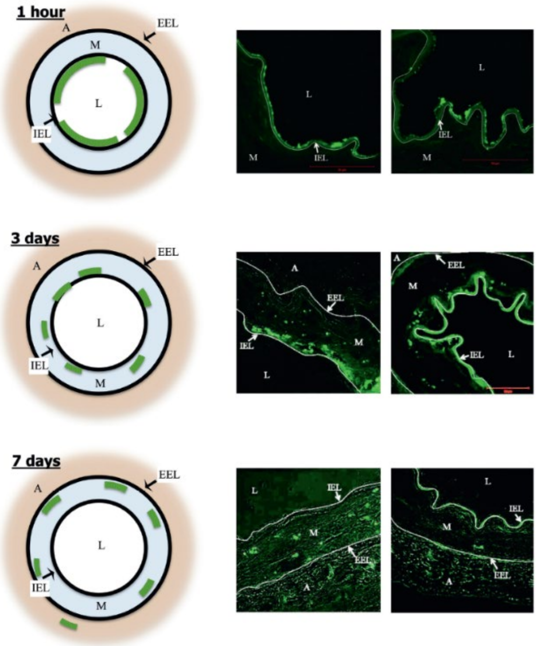

Figure 2: Temporal penetration of labeled sirolimus nanoparticles

Sirolimus

The active pharmaceutical ingredient (API) in the Magic Touch PTA formulation is sirolimus and is manufactured by Biocon Ltd. Sirolimus (rapamycin) was originally developed in 1975 as a macrolide antibiotic produced by the bacterium Streptomyces hygroscopicus, it was well known for its antifungal, immunosuppressant and antitumor properties and was approved for use in the Cordis CYPHER Sirolimus Eluting Coronary Stent System (PMA No.: P020026). As with DES, it is anticipated that the anti-proliferative and anti-inflammatory properties of sirolimus will also reduce the tendency for restenosis following balloon angioplasty. The sirolimus dose provided by the Magic Touch System is 1.27µg/mm^2.

Excipient

Lipoid E80 manufactured by Lipoid GmbH is utilized as excipient in the Magic Touch PTA. Lipoid E80 is also known as Egg Lecithin ~ 80% of with Phosphatidyl choline contents. As phospholipids are main components of any cellular membrane, they have excellent biocompatibility and are renowned for their amphiphilic structure, providing them with wetting characteristics, and the ability to enhance the hydrophilicity of hydrophobic drugs. The use of phospholipids as excipient drug carriers have been Generally Recognized As Safe (GRAS) by the FDA and various regulatory bodies. Lipoid E 80 has blend of phospholipids, which has been shown to support wound healing when used as a part of the drug delivery systems.

Manufacturing process

The production and manufacturing of the Magic Touch PTA is performed by Concept Medical in the following facility:

Envision Scientific Private Limited
Plot No. C1B 108 Ichchhapore GIDC Industrial Estate,
Bhatpore, Sachin – Hazira Road,
Surat – 394 510 INDIA

The ETO sterilization is performed by Envision Scientific Private Limited at the following facility:

Envision Scientific Private Limited

Plot No. C1B 108 Ichchhapore GIDC Industrial Estate,
Bhatpore, Sachin – Hazira Road,
Surat – 394 510 INDIA

Each batch of finished product undergoes full testing before release and distribution. The test method used follows the International Conference Harmonization Guidelines (ICH).

Brief Steps of Manufacturing are as follows:

Submicron sized drug carrier encapsulation

Sirolimus and Lipoid E80 are encapsulated by ultrasonic homogenization process which further prepares formulation for coating.

Spray Coating and Drying

Spray coating of formulation is done using an inert gas assisted process in circumferential pattern. The coated balloon catheter is analyzed by light microscopy to evaluate surface morphology, characteristics, smoothness, and coating defects. An image of each coated balloon is archived according to unique serial number. The drug content is also measured and evaluated as per in-house specification before lot release.

Primary Packaging

Magic Touch PTA is sealed in Tyvek 1073 pouch using validated sealing method. Device after sterilization remains sterile after sterilization process.

Sterilization

The Magic Touch PTA is sterilized using ethylene oxide sterilization and has been validated per ISO 11135:2014: Sterilization of health care products – Ethylene Oxide: Requirements for development, validation, and routine control of a sterilization process for medical devices. The testing for ethylene-oxide residuals was completed and found acceptable per ISO 10993-7:2008. Results obtained for the sterilization studies demonstrated a minimum sterility assurance level (SAL) of 10-6. Bacterial endotoxin was verified to be within the specification limit for the Magic Touch PTA. The sterilization validation will be provided to FDA in the IDE submission.

Aluminum Pouch Sealing: Tyvek sealed pouches are, further sealed in Aluminum pouch along with label.

Final Package

Aluminum pouch is further packed in cardboard box with appropriate labelling information. Inside box, instruction for Use and compliance chart is inserted.

4.3 Preclinical Evidence

The Magic Touch Sirolimus Coated Balloon Catheter is a safe and effective treatment in coronary application. Magic Touch PTA (formerly known as Xtreme Touch and Xtreme Touch Neo) balloons were used in the swine peripheral artery 28 days post treatment. Histologic evaluation of the 1x therapeutic and 3x safety margin dosing by Magic Touch PTA balloons showed a drug treatment effect recognized by focal superficial accumulated proteoglycan-rich matrix with extension into the deeper media accompanied by minimal underlying medial smooth muscle cell loss. The drug treatment effect was generally more apparent with 3x safety margin dosing for peripheral vessels.

Analysis of downstream myocardial tissue or skeletal or muscle / coronary band sections showed only rare evidence of fibrin rich embolies not related of Magic Touch PTA balloon and showed safety at 28 days (Figure below).

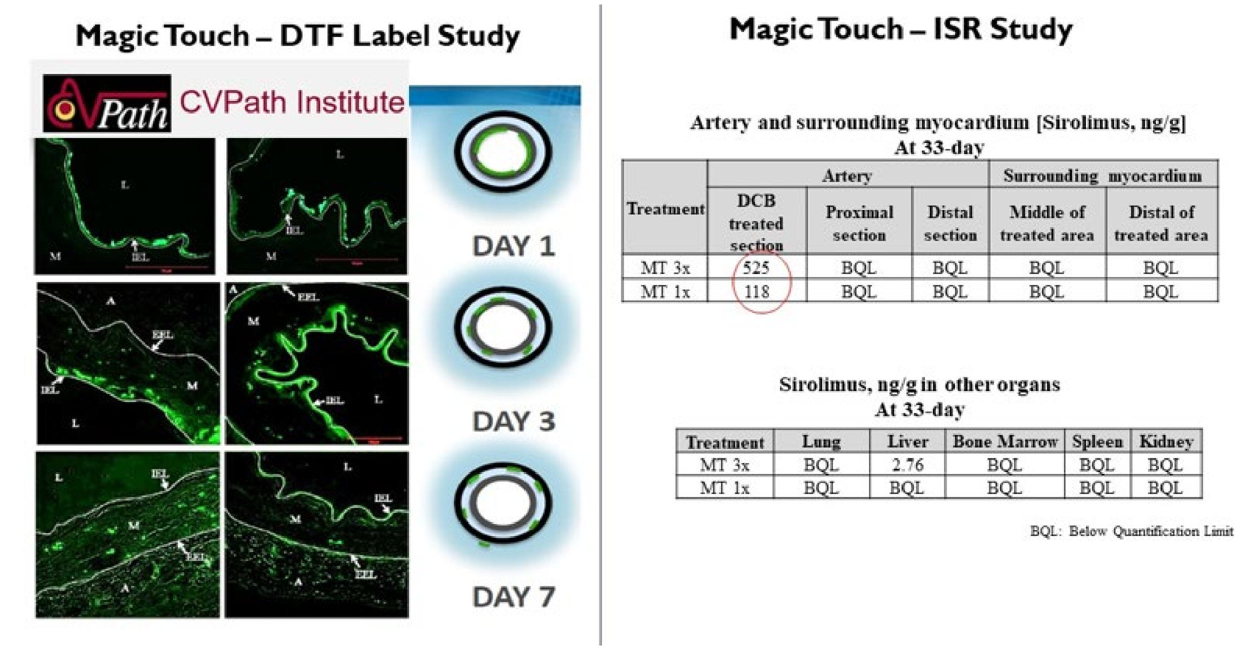

Treatment	Artery			Surrounding myocardium	
	DCB treated section	Proximal section	Distal section	Middle of treated area	Distal of treated area
MT 3x	525	BQL	BQL	BQL	BQL
MT 1x	118	BQL	BQL	BQL	BQL

Sirolimus, ng/g in other organs
At 33-day

Treatment	Lung	Liver	Bone Marrow	Spleen	Kidney
MT 3x	BQL	2.76	BQL	BQL	BQL
MT 1x	BQL	BQL	BQL	BQL	BQL

BQL: Below Quantification Limit

Figure 3: Magic Touch in-vivo study

In another animal study evaluating blood and tissue levels in rabbits after treatment with Magic Touch found that blood concentration decreased rapidly after a single 60 second deployment, while tissue concentration was still detectable after two weeks (Figure below).

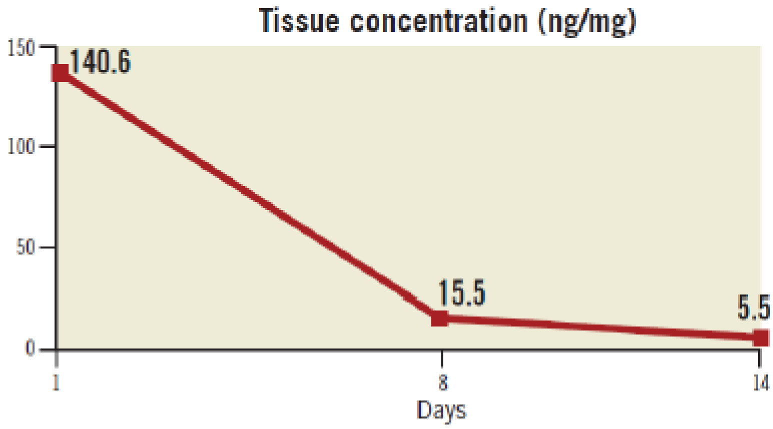

Figure 4: Tissue Concentration of sirolimus after single 60-second inflation

4.4 Clinical Evidence to Date

Concept Medical has conducted a single-center (Sengkang General Hospital, Singapore), first-in-human, prospective interventional study (the XTOSI study) to investigate the efficacy and safety of a sirolimus-coated balloon in patients with peripheral arterial disease (PAD) undergoing endovascular revascularization. The study was approved by the Local Ethics Committee (CIRB Ref: 2018/2419) and was carried out in accordance with the Helsinki Declaration. The results of the interim analysis (N=38) have been presented at the 2019 TCT Congress in San Francisco and have been submitted to the FDA before approval of the device.

The primary outcomes were:

1. Freedom from clinically-drive target lesion re-vascularization (TLR) at 12 months;

2. Freedom from Major Adverse Events (MAE) at 30 days, a composite of freedom from device- and procedure-related mortality, and major target limb amputation through to 30 days;
3. Freedom from clinically driven TLR within 6 months post-index procedure;

Inclusion criteria for the study were:

1. Age ≥ 21 years or minimum age;
2. Willing to sign a Patient Informed Consent;
3. Lesion(s) in the infra-inguinal arteries suitable for endovascular treatment treated with or scheduled to be treated with the Magic Touch PTA (previously known as Xtreme Touch – Neo) sirolimus PTA balloon catheter.

Exclusion criteria for the study were:

1. Life expectancy ≤ 1 year;
2. Currently participating in another investigation drug or device study that has not reached first primary endpoint;
3. Pregnant or planning to become pregnant during the course of the study;
4. Failure to successfully cross the target lesion with a guide wire (successfully crossing means tip of the guide wire distal to the target lesion in the absence of flow limiting dissections or perforations.

Thirty-eight patients with 48 lesions were enrolled in the study. Of the 38 patients, 89% and 87% of patients had diabetes mellitus and hypertension, respectively.

The mean lesion length for anterior tibial artery and posterior tibial artery to be treated with Magic Touch PTA was 264.2 mm and 250.0 mm, respectively. Freedom from MALE at 30 days was 95% [n=36/38], where two patients underwent major limb (below knee) amputation due to severe sepsis of the foot post-angioplasty. Freedom from device- and procedure-related mortality was 100%. Freedom from clinically-drive TRL within 6 months post-index procedure was available for 11 patients and was reported to be 91% [n=10/11]. Based on the results of the study to date, Magic Touch PTA has a good safety profile at 30 days.

Interim analysis for a subgroup of 12 subjects showed efficacy for below the knee indication, in which primary patency by ultrasound at 6 months was 82% and freedom from TLR was 91%. The loss of primary patency and TLR both involved below the knee arteries.

4.5 Explanation for Choice of Comparator

The comparator will be any available CE-certified uncoated balloon catheter with application in peripheral artery disease approved for patient use in Switzerland. In contrast to the investigation medical device, there are several uncoated balloon catheters for peripheral interventions available that are used in daily clinical practice interchangeably, in the absence of a standard of care.

For the purposes of this trial, we have decided to allow the use of all uncoated products to:

- minimize the extent of screening failures;
- avoid head-to-head comparisons between the interventional product and a specific medical device, since in this field there is no standard of care which could be used as the comparator.

4.6 Risk / Benefits

The investigators have no ethical concerns in regard to the efficacy and safety of the medical devices that will be used in the trial. Currently, both drug-coated and uncoated strategies are approved treatment option for patients with peripheral artery disease. All devices that will be used are CE certified and approved for use in peripheral artery disease.

4.7 Justification of Choice of Study Population

In order to achieve an all-comer study, and in contrast to prior trials in PAD patient, we have limited the inclusion and exclusion criteria to a minimum. The study population will consist of adult patients with peripheral artery disease, who are scheduled for infra-inguinal angioplasty based on easy and reproducible clinical and angiographic criteria. Only patients allergic to sirolimus and pregnant women will be excluded a priori. All patients fulfilling the inclusion criteria without having any of the aforementioned exclusion criteria will be screened. Patients will be recruited from our outpatient clinics (hubs or satellites) or when referred by emergency room physicians, by physicians of hospitalized patients from other departments, or by external vascular specialists. Participants temporarily or permanently incapable of judgment, as well as and participants under tutelage, will be included, since they represent an important part of the PAD population in daily clinical practice and are often characterized by more advanced stages of disease, possibly benefitting more from a balloon catheter intervention. Requirements and procedures for

5 STUDY OBJECTIVES

5.1 Primary Objective

The purpose of this study is to evaluate whether the use of sirolimus-coated balloon catheters is non-inferior to uncoated balloon catheters in infra-inguinal angioplasty to prevent one-year major adverse limb events, including unplanned major amputation of the target limb and target lesion re-vascularization to treat critical limb ischemia, in a representative population (`all-comers`) of patients with PAD.

If this objective is reached, namely if the criteria for non-inferiority are confirmed, the study will test whether sirolimus-coated catheters are superior to uncoated catheters for important secondary outcomes and for the primary outcome itself according to a pre-specified hierarchical analysis of outcomes based on prior literature data and clinical relevance.

5.2 Secondary Objective

Since concerns had been raised by regulatory agencies in regards of the elevated mid-term mortality observed in patients exposed to paclitaxel-coated devices (versus that in patients treated with uncoated devices), we will assess the safety of the intervention by measuring two-year mortality, the primary safety endpoint.

Subgroup analyses to study the heterogeneity of treatment effects will be conducted, i.e. in patients categorized by sex, and elective vs. critical rescue re-vascularization.

6 STUDY OUTCOMES

6.1 Primary Outcome

The primary efficacy outcome is a composite of two major adverse limb events (MALE):

(iii) unplanned major amputation of the target limb, and
(iv) endovascular or surgical target lesion re-vascularization for critical limb ischemia

occurring within one year of enrolment.

Definitions

- An unplanned major amputation is defined as any amputation above the ankle on the target limb, which was not planned or not expectable at the time of screening or randomization. Patients with scheduled amputation undergoing re-vascularization to improve wound healing are referred to as planned amputation and will not count for the primary outcome.
- Critical limb ischemia is defined according to a Fontaine stage (classes III-IV; Appendix 18.1).

6.2 Secondary Outcomes

The secondary outcomes are:

- A composite of unplanned (major or minor) index-limb amputations or any target lesion re-vascularization within 365 days after enrolment (tested in the hierarchical analysis if criteria for non-inferiority are fulfilled);
- Clinical improvement by $\geq$1 Rutherford category through 180 days and 365 days;
- Any target lesion re-vascularization performed within 365 days after enrolment;
- Target lesion re-vascularization for non-critical limb ischemia performed within 365 days;
- Target lesion re-vascularization for critical limb ischemia within 365 days after enrolment;
- Target limb re-vascularization within 365 days after enrolment;
- Unplanned minor amputation at target limb performed within 365 days after enrolment;
- Unplanned major amputation at target limb performed within 365 days after enrolment;
- Any unplanned amputation;

Definitions

- ➢ Unplanned major amputation at is defined analogously to the definition used for the primary efficacy endpoint (see above).
- ➢ Unplanned minor amputation is defined as amputation below or at the level of the ankle, which was not planned or not expected at the time of randomization.
- ➢ Non-critical limb ischemia is defined as patients with Fontaine stage I-II.
- ➢ Critical limb ischemia is defined as patients with Fontaine stage III-IV.
- ➢ Freedom from target lesion re-vascularization is defined as the percentage of patients without the occurrence of re-intervention (surgical or interventional) at the target lesion irrespective of any re-intervention out of the target lesion.
- ➢ Freedom from target limb re-vascularization is defined as the percentage of patients without the occurrence of re-intervention (surgical or interventional) at the target limb irrespective of any re-intervention out of the target limb.

6.3 Safety Outcomes

- Death from all causes within 30 days, 180 days, one year, two years, and five years.
- Serious adverse events (SAEs) during initial hospitalization, within 180 days, and within 365 days.
- Serious adverse device related events (SADE) during initial hospitalization.
- A composite of all-cause death and MALE within 30 days.

The occurrence of safety outcomes, including vital status of patients, will be verified at each scheduled visit or time-point, including hospital discharge, interim visit (Day 180±30), and one-year visit (Day 365±21), or if it is being reported by patients at any time during follow-up. The vital status of patients will be verified until study termination (Year 5).

The primary sources for verifying the patients´ vital status will be in-person contact with the relatives and treating physicians, internal and external medical reports (including discharge letters including the vital status and causes of death), and administrative vital registration data ("Zivilstand").

7 STUDY DESIGN AND COURSE OF STUDY

7.1 General Study Design and Justification of the Design

The SirPAD trial is an academic, investigator-initiated, multi-center, randomized, non-inferiority, open-label clinical trial investigating whether the use of sirolimus-coated balloon catheters in patients with peripheral artery disease of the femoro-popliteal or below-the-knee segment is not inferior to that of uncoated balloon catheters for major clinical outcomes (unplanned major amputation, target limb re-vascularization) and may provide advantages concerning important secondary outcomes, which will be evaluated using a pre-specified hierarchical order as part of the primary analysis.

Population

The SirPAD trial pursues the concept of an all-comer study, therefore aiming to maximize external validity by reducing to a minimum the number of exclusion criteria and achieving high enrolment rate. The heterogeneity of eligibility criteria observed between prior studies and, at the same time, their strictness are some of the reasons why current evidence may fall short in providing adequate estimates of the efficacy and safety of drug-coated balloon catheters (primarily, paclitaxel-coated), as compared with the uncoated ones.
The inclusion of a highly selected population of PAD patients would limit the external validity of the study and lead to a low rate of events, therefore supporting false claims of non-inferiority. In the present study, we attempted to minimize the eligibility criteria and enroll a high risk population, which includes not only patients with chronic symptoms, but also those with acute limb ischemia or chronic critical limb ischemia requiring immediate revascularization.

Non-inferiority design and choice of the intervention

A non-inferiority trial design, comparing a new treatment with a standard, is frequently used because of the need to replace standard treatments by other treatments having comparable efficacy but i.e. presenting with other clinical or practical advantages. We decided to embrace a non-inferiority design for the following reasons:

- drug-coated and uncoated balloon catheters are considered therapeutic interchangeable in the PAD community of physicians and caregivers: however, this has never been proven, particularly with respect to novel devices;
- we cannot anticipate a meaningful difference for the occurrence of a ‘hard’ primary outcome (MALE) within one year from intervention since no information from pilot trials or registries is available. However, we hypothesize that the results of trials comparing paclitaxel-coated catheters with uncoated catheters can partly apply to sirolimus-coated devices. Therefore, further confirmatory statistical tests for superiority will be performed using a hierarchical order of secondary outcomes based on their clinical importance.

Non-inferiority margin

The non-inferiority margin is justified if it is selected in order to preserve a major portion of the efficacy of standard of care versus placebo or no treatment. In the present study, we expect that a heterogeneous population of patients with different individual risk of MALE will be included, characterized by a rate of complications, as defined according with our primary outcome, of approximately 20-25% if left untreated or receiving placebo.
This value was estimated based on the individual risk of complications of patients with acute limb ischemia requiring acute revascularization (~17% of expected SirPAD study population), with chronic limb ischemia with wounds (~41%), or with claudication (~42%).
The one-year rates of the primary efficacy outcome (MALE) in patients receiving the standard of care (endovascular or surgical) versus untreated were estimated to be 10% and 26%, respectively. (Table 3)
Reliable estimates of target limb re-vascularization rates for chronic critical limb ischemia in our population is are not available in the literature to date. Expected rates can be only extrapolated from previous studies with similar outcomes, but often mixed populations (Table 3-6): therefore, we partly based the present estimates on own clinical experience. The rates of clinically driven target limb re-vascularization were used as a sensitivity margin, which is unlikely to be exceeded in the SirPAD study.

The non-inferiority margin adopted in the present study (absolute risk difference between sirolimus-coated and uncoated balloon catheters of 5%, e.g. 15% vs. 10%) will therefore preserve approximately 60% of the efficacy of standard of care versus placebo (10% vs 26% → 15% vs. 26%).

Table 3: Expected primary efficacy rates for study population

	**Acute limb ischemia**	**Chronic critical limb ischemia**	**Claudication**	**Estimation**
Estimated percentages of PAD stage for study population based on center experience				
	17%	41%	42%	-
Expected major amputation rate at 12 months				
Untreated	95%	22%[13]	1%	26%
Treated	7%	6%	1%	4%
Expected target limb revascularization for critical limb ischemia at 12 months				
Untreated	-	-	-	
Treated*	10%	10%	1%	6%
*No data available estimating target limb re-vascularization rates for critical limb ischemia in patients with acute limb ischemia, critical limb ischemia or claudication. The rates of clinically driven target limb re-vascularization were used as a sensitivity margin, which is unlikely to be exceeded in the SirPAD population. Abbreviations: PAD, peripheral artery disease;				

Table 4: Event rates on clinical driven target limb re-vascularization (TLR) for different populations

**Outcome**	**Main population**	**Intervention**	**Comparator**	**Study**
Clinically driven TLR	Claudication (N=479)	DES: 9.5% (12 mo)	BMS: 17.5% (12 mo)	RCT[3]
Clinically driven TLR	Claudication (N=476)	DCB: 13.3% (12 mo)	POBA: 18.2% (12 mo)	RCT[4]
Clinically driven TLR	Claudication (N=101)	DCB: 29% (12 mo)	POBA: 37% (12 mo)	RCT[5]
Clinically driven TLR	Claudication (N=331)	DCB: 2.4% (12 mo)	POBA: 20.6% (12 mo)	RCT[7]
Clinically driven TLR	Claudication (N=196)	BMS: 8.4% (12 mo)	-	Single arm[14]
Clinically driven TLR	Claudication (N=57)	DES: 3.5% (12 mo)	-	Single arm[8]
Clinically driven TLR	Critical limb ischemia (N=358)	DCB: 9.2% (12 mo)	POBA: 13.3% (12 mo)	RCT[6]
Clinically driven TLR	Critical limb ischemia (N=263)	Endo: 15.2% (3 mo)	-	Retrospective[15]

Clinically driven TLR	Acute limb ischemia (N=590)	Endo: 32.9% (59 mo)	-	Retrospective[16]
Abbreviations: TLR, target lesion re-vascularization; DES, drug-eluting stent; BMS, bare metal stent; RCT, randomized-controlled trial; DCB, drug-coated balloon; POBA, plain-old balloon angioplasty; Endo, endovascular therapy				

Table 5: Event rates on major amputation for different populations

**Outcome**	**Main population**	**Intervention**	**Comparator**	**Study**
Major amputation	Acute limb ischemia (N=147)	Mixed (69% Endo) 13% (12 mo)	-	Retrospective[17]
Major amputation	Acute limb ischemia (N=544)	Endo: 6.5% (12 mo)	Surgery: 7.5% (12 mo)	RCT[18]
Major amputation	Acute limb ischemia (N=590)	Endo: 17.7% (12 mo)	-	Retrospective[16]
Major amputation	Claudication (N=57)	DES: 0% (12 mo)	-	Single arm[8]
Major amputation	Claudication (N=196)	BMS: 0% (12 mo)	-	Single arm[14]
Major amputation	Claudication (N=479)	DES: 0% (12 mo)	BMS: 0% (12 mo)	RCT[3]
Major amputation	Claudication (N=476)	DCB: 0.3% (12 mo)	POBA: 0% (12 mo)	RCT[4]
Major amputation	Claudication (N=101)	DCB: 2% (12 mo)	POBA: 0% (12 mo)	RCT[5]
Major amputation	Claudication (N=331)	DCB: 0% (12 mo)	POBA: 0% (12 mo)	RCT[7]
Major amputation	Critical limb ischemia (N=263)	Endo: 24.7% (29 mo)	-	Retrospective[15]
Major amputation	Critical limb ischemia (N=358)	DCB: 8.8% (12 mo)	POBA: 3.6% (12 mo)	RCT[6]
Major amputation	Mixed: Inpatient hospitalization within 1 year after re-vascularization (N=384415)	Mixed: 3.5% (12 mo)	-	National database[19]
Major amputation	Stabile PAD (N=7470)	Rivaroxaban+ASS: <1.0% (21 mo)	ASS alone: 1.0% (21 mo)	RCT[20]
Abbreviations: Endo, endovascular therapy; DES, drug-eluting stent; BMS, bare metal stent; RCT, randomized-controlled trial; DCB, drug-coated balloon; POBA, plain-old balloon angioplasty; ASS, acetylsalycic acid				

Table 6: Event rates on other outcomes for different populations

**Outcome**	**Main population**	**Intervention**	**Comparator**	**Study**
Any re-vascularization	Mixed: Inpatient hospitalization within 1 year after re-vascularization (N=384415)	Mixed: 6.0% (12 mo)	-	National database [19]

MALE	Mixed: Inpatient hospitalization within 1 year after re-vascularization (N=384415)	Mixed: 10.3% (12 mo)	-	National database[19]
MALE	Claudication (N=3925)	Endo: 3.2% (1 mo)	Surgery: 4.0% (1 mo)	National database[21]
MALE	Critical limb ischemia (N=13294)	Endo: 12.2% (1 mo)	Surgery: 9.2% (1 mo)	National database[22]
MALE	Stabile PAD (N=7470)	Rivaroxaban+ASS: 1.0% (21 mo)	ASS alone: 2.0% (21 mo)	RCT[20]
MALE	Symptomatic PAD (N=3642)	Subgroup of all patients (PCSK9 or Placebo) with PAD: 1.5% (12 mo)	-	RCT[23]
Abbreviations: MALE, major adverse limb events (different definitions may apply); Endo, endvascular therapy; ASS, acetylsalycic acid; RCT, randomized-controlled trial; PAD, peripheral artery disease				

Intervention and Comparator

Sirolimus-coated balloon catheters are approved for treatment of PAD in Europe and in the US: due to current regulations, their efficacy and safety has not been established in the setting of a RCT. As there might be an increased long-term risk of death following application of paclitaxel-coated balloons, sirolimus-coated (and not paclitaxel-coated) balloon catheters will serve as the interventional device.

Uncoated balloon catheters have been used as a comparator in prior trials enrolling PAD patients. Below-the-knee or infra-popliteal PAD has limited treatment options. Currently, most patients present with critical limb ischemia (gangrene, ulceration of extremity or rest pain), but the diffuse nature of the disease and small caliber vessels pose unique treatment challenge. Balloon angioplasty is associated with high restenosis rates and stents have limited short term patency due to small vessel size and long segment stenosis: therefore, the use of drug-coated balloon use may prove non-inferior, and possibly more effective for important secondary outcomes, in patients with infra-popliteal PAD.

Primary efficacy outcome

The components of the primary efficacy outcome were chosen:

(1) to minimize the risk of any subjective assessment of the outcomes,
(2) since it is likely that they are highly sensitive to the intervention,
(3) in light of their ease of assessment, which does not require a core-lab for adjudication and is less subject to loss to follow-up.

One-sided type I error

In non-inferiority trials, the investigators already accept a reasonable margin of reduced efficacy (non-inferiority margin). It remains crucial that stringent criteria are followed to avoid additional sources of bias that could lead into potentially false claims of non-inferiority. A one-sided α of 0.025, which is consistent with a 2-sided 95% CI, is considered preferable.

Blinded adjudication

No major influence of blinding on the primary outcome (MALE) is anticipated in light of the ease of assessment of MALE and lack of subjective interpretation, which may be prone to bias.

Centers

The study will be conducted at two, large-volume, academic hospitals with experience in the treatment of PAD patients. The involvement of other centers was considered unfeasible from a ´all-comers´ perspective, as several competing trials would have been running in parallel, therefore leading to a pre-selection of potential candidates for participation.

7.2 Study Duration and Study Schedule

The duration of the entire clinical trial for each patient will be 365 days followed by an additional 365 days of follow-up at the end of which only survival will be assessed. The clinical trial will close when all patients will complete the planned study period. Study begin is defined as first patient first in (FPI) and study end as last patient last visit (LPO).

The Clinic of Angiology of the University Hospital of Zurich alone currently performs approximately 800 peripheral interventions per year. The Clinic of Angiology of the Fribourg Hospital performs approximately 250 peripheral interventions per year. Therefore, assuming that more than 80% of the eligible patients will be enrolled in the study, the patient recruitment is expected to last no longer than 30 months for a total duration of the study of 54 months, accounting for the enrolment period followed by a 12-month follow-up for clinical outcomes and 5-year follow-up for mortality.

First-Patient-In: Q4 2020
Last-Patient-Out: Q2 2028

7.3 Methods of Minimizing Bias

7.3.1 Randomization

After the angiographic screening and definition of the target lesion, but prior to any therapeutic intervention, each eligible patient will be assigned to one of the two open treatment arms of this trial by an online randomization tool. Randomization will be stratified according to the stage of disease:

- Elective re-vascularization (Fontaine stage I-II) vs. critical rescue re-vascularization (Fontaine stage III-IV)

7.3.2 Other Methods of Minimizing Bias

Due to the definition of explicit primary endpoints (major amputation, re-vascularization for critical limb ischemia, all-cause mortality), routine follow-up visits, prospective telephone and patient chart follow-ups, and the use of individual civil registration mortality data, we will be able to generate accurate data points for the majority of patients. Allocation concealment will guarantee that no selection bias will affect the results.

In case a patient is lost to follow-up and no information can be obtained for the primary outcome (in-hospital visit, telephonic contact, access of the medical charts), the clinical outcome committee decides whether this case has to be considered as an event or not, based on the information available for the patient. It is expected that the information whether a patient is still alive or dead can be obtained with certainty by contacting the administrative offices, if necessary.

A sensitivity analysis will be conducted in which missing data for the primary outcome will be imputed according to the worst case principle (outcome reached). The same analysis will be conducted in case of large differences between the intention-to-treat and the per-protocol populations.

7.4 Unblinding Procedures (Code break)

This clinical trial will be conducted open-label. Therefore, both the patients and the investigators will be aware of the group allocation, and unblinding will not be performed.

8 STUDY POPULATION

Adult patients with “peripheral artery disease requiring infra-inguinal endovascular angioplasty” are eligible for clinical trial participation if they meet all of the inclusion and none of the exclusion criteria listed in the eligibility below.

8.1 Eligibility Criteria

8.1.1 Inclusion Criteria

Participants fulfilling all of the following inclusion criteria are eligible for the study:

- Age $\geq$ 18 years;
- Required endovascular angioplasty for peripheral artery disease (target lesion) located below the inguinal ligament. A target lesion is defined as a the main lesion considered responsible for the patient´s signs and symptoms, fulfilling the following angiographic criteria:
  - i. stenosis (lumen compromise ≥50%) in at least a single plane of the femoro-popliteal arterial segment including the femoral, deep femoral and or popliteal artery or a femoropopliteal bypass, or
  - ii. stenosis (lumen compromise ≥50%) of the below-the-knee arterial segment including the tibioperoneal trunk and/or the anterior tibial, peroneal, or posterior tibial artery, or a below the knee bypass.
- Written informed consent obtained from patient or legal guardian in case of tutelage prior to randomization; in patients requiring emergency interventional treatment who are temporarily not capable of providing informed consent, consent will be subsequently obtained after the procedure if strict conditions apply. These include the assessment of the presumed will and patient decree and requires the allocation of an independent physician (for details: see 4.6).

8.1.2 Exclusion Criteria

The presence of any one of the following exclusion criteria will lead to exclusion of the participant:

- Pregnancy, breastfeeding, or planned pregnancy within the trial period or women of childbearing potential not using an adequate method of contraception.
- Patients with known intolerance or allergy to sirolimus.
- Participation in this or other clinical trials during the previous 3 months.

8.2 Recruitment and Screening

The screening phase will be composed of two phases: (i) clinical screening and (ii) angiographic screening. Both phases will take place at the study center (*Clinic for Angiology, University Hospital of Zurich)* and involve patients with peripheral artery disease referred for evaluation of re-vascularization or direct referral for re-vascularization by study center physicians or other treating physicians, as per routine procedures. No additional information (handouts, brochures) will be given to potential participants or physicians from other centers.

Potentially eligible patients will receive the patient information and informed consent forms from the principal or sub-investigators before screening.

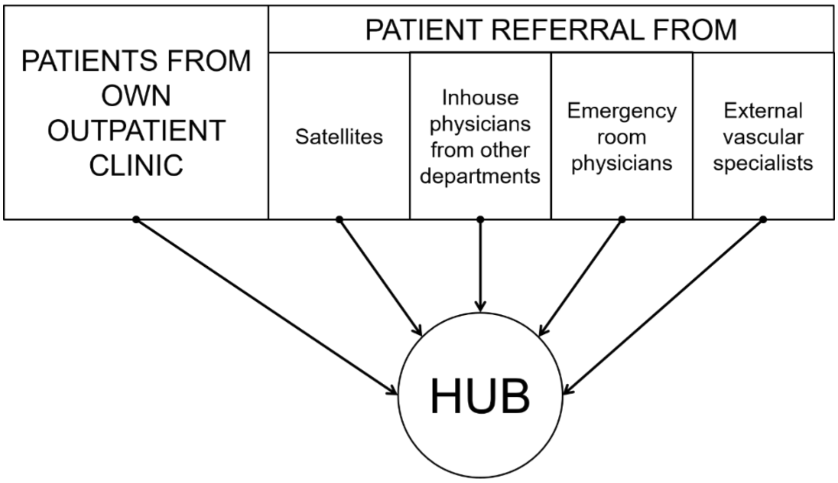

Figure 5: Patient referral

8.2.1 Clinical screening

Clinical screening will be performed at the study center after the patient has given consent for screening and participation in the study. During the consent discussion, the investigator will use native non-technical language to fully inform the patient (or legal representative) of all aspects of the study. The patient will also be informed explicitly about his/her right to withdraw from the study at any time and without giving reason, sanction, loss of benefits or penalty. The background and purpose of the study as well as the potential benefits and risks of the procedure(s) will be explained to the subject. The patient will have enough time and opportunity to inquire about details of the study, and to decide whether or not to participate. All questions of the patient will be answered sufficiently. If the patient is visually impaired, extra time will be made available in order to allow a family member (or a local study investigator together with a witness in accordance with Art. 8 of the ClinO.) to read to him/her the written informed consent. If the patient is not proficient in any of the languages used for written patient informed consent, one of the family member (or a professional translator together with a witness) will translate them and further discuss with the investigators the implications of participation in this trial.

Once all questions have been answered, and the patient has decided to participate, the informed consent will be obtained in writing including signature (patients and investigator), place and date. The informed consent will be scanned and imported into the clinical information system (KISIM). In addition, the participants will be marked electronically as *study participant* in the KISIM. The original document will be stored (and locked) at the study coordinators office. We will not advertise for study enrolment in any form, and no monetary compensation will be provided for participants.

The clinical screening will consist of evaluation of inclusion and exclusion criteria including a duplex ultrasound exam or evaluation of a recently performed one. The clinical screening will not involve any procedures that are not part of the routine evaluation of these patients.

The investigator will document “screening failure”, if the patient is eligible but does not consent to participate. Reasons for not participation (patient not eligible) and screening failures will be collected according to a predefined form, as summarized in the CONSORT flowchart below.

8.2.2 Angiographic screening

Angiographic screening will be performed and documented by an investigator (not delegable) on the day of the intervention. If angiography reveals no infra-inguinal target lesion, the investigators will document “screening failure” in the screening failure log (see 8.3). If angiography confirms infra-inguinal target lesion, the target lesion will be defined, and the patient will undergo randomization.

In patients receiving lysis catheters, angiographic screening will be performed on the day of the second-look angiography prior to any other re-vascularization procedures. In patients receiving pharmaco-

mechanical or manual aspiration thrombectomy, angiographic screening will be performed before first balloon angioplasty.

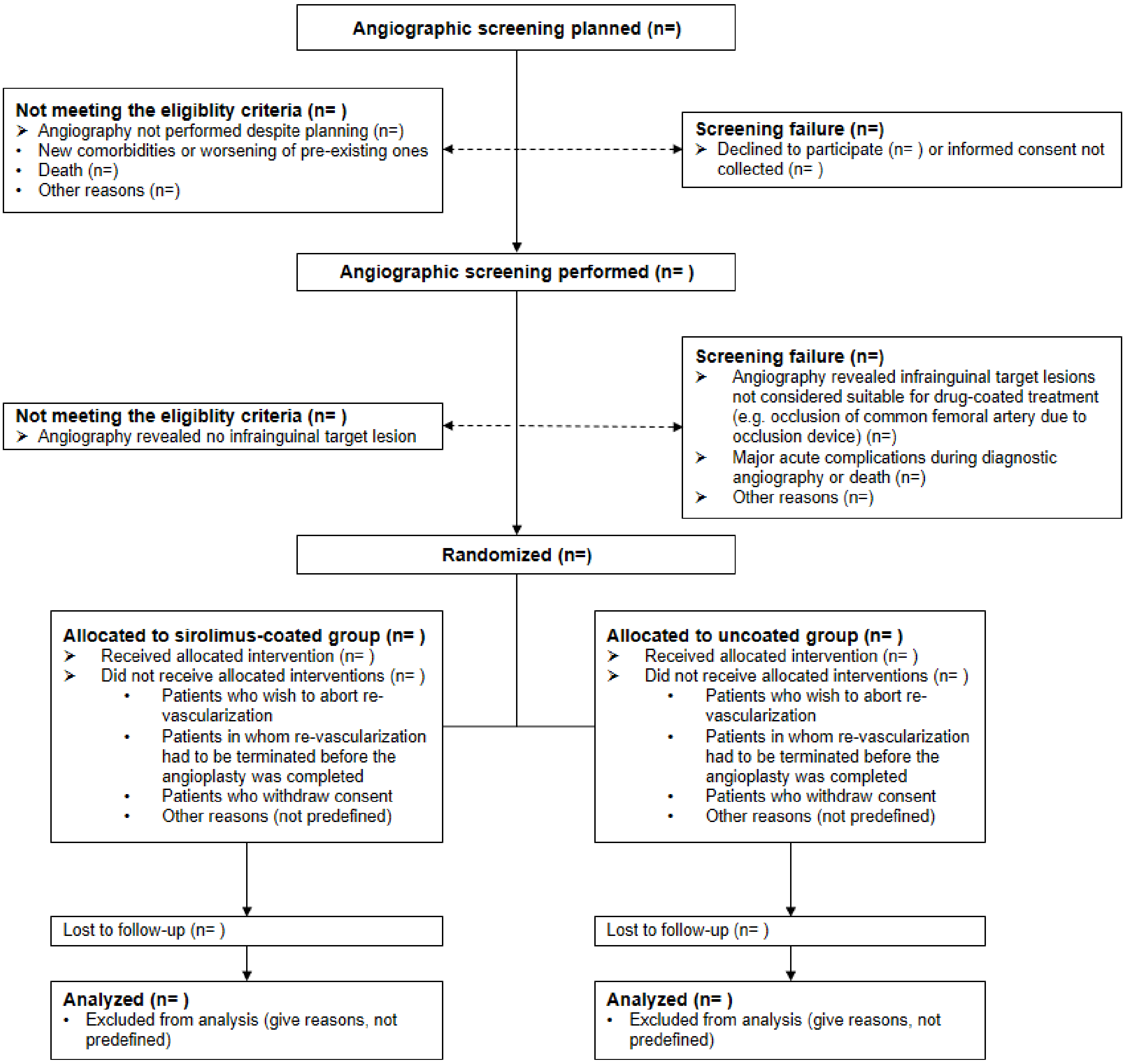

Figure 6: Progress of patients through the trial

8.2.3 Assignment to Study Groups

Randomization will be performed in the catheter lab immediately after the angiographic assessment and identification of the target lesion. Randomization will be performed by an instructed assistant, study coordinator, or investigator using an online random tool (REDCap, Vanderbilt University, v9 1.0). Allocation will be concealed and treatment will be done immediately thereafter during the same angiographic session.

8.3 Criteria for Withdrawal/ Discontinuation of Participants

The patient is entitled to terminate the clinical trial at any time without giving any reasons and without experiencing any disadvantages. Patients, who discontinue the clinical trial participation before randomization, will be logged as screening failures.

This includes patients

- who decline to participate, or patients in whom the informed consent was not collected;
- in whom angiography was not performed (comorbidities, death, other reasons);
- in whom angiography reveals infra-inguinal target lesion that is not considered suitable for drug-coated treatment;
- in whom angiography had to be terminated due to major complications or death:
  - allergic reactions;
  - safety concerns to continue procedure (i.e. patient not compliant with the treating physician`s instruction);
  - major acute complications or deterioration of a known medical condition;
  - death;

Patients, who discontinue the clinical trial participation prematurely after randomization and allocation, will not be replaced (drop-outs).

This includes patients

- who wish to abort re-vascularization;
- who wish to terminate participation in the clinical trial and withdraw consent;
- in whom re-vascularization therapy had to be terminated before the angioplasty was initiated or terminated due to safety reasons:
  - allergic reaction
  - no compliance (safety concerns to continue procedure)
  - major acute complications or deterioration of a known medical condition.

Since the treatment phase of the study ends with the termination of re-vascularization procedure, there are no scenarios in which patients need to be withdraw from participation for safety reason during the follow-up period. However, enrolment of the study may be prematurely stopped before reaching the planned number of participants if the results of the interim analysis are significant for the primary efficacy or primary safety outcome.

9 STUDY INTERVENTION

9.1 Identity of Investigational Medical Device

All interventions will be performed according to standard treatment of care. All study devices used have been approved for the current indication and are already in routine clinical use.

List of material, treatments or diagnostic agents used during intervention (standard of care):

	**Experimental Intervention**	**Control intervention**
Angiographic 18-Gauge needle, length 70 mm for 0.038 inch<br>*optional: 21-Gauge needle, length 70 mm, 0.018 inch*	+	+
0.035 inch wire<br>*optional: 0.018, 0.014 inch*	+	+
Standard 4-French sheath (vascular access)	+	+
Contrast enhancement: Ultravist-300 (iopromide) administered intra-arterial via sheath / catheter	+	+
CE-certified uncoated balloon catheters with application in peripheral arterial disease approved for inpatient use in Switzerland.[1]	+	+
Optional: CE-certified uncoated vascular stent with application in peripheral arterial disease approved for inpatient use in Switzerland.[2]	+	+
Intervention: MagicTouch PTA sirolimus drug-coated balloon catheter	+	-

[1] Including, but not limited to:

- Mustang 0.035'' Balloon Dilatation Catheter (Boston scientific)
  3-12 mm diameter, 20-200 mm length
- Sterling 0.018'' Balloon Dilatation Catheter (Boston scientific)
  2-3 mm diameter, 20-220 mm length
- Coyote 0.014''Balloon Dilatation Catheter (Boston scientific)
  1-4 mm diameter, 10-200 mm length

[2] Including, but not limited to:

- Life Self expanding Peripheral Stent (Bard)
  5-8 mm diameter, 20-200 mm length
- EverFlex Self-expanding Peripheral Stent (Medtronic)
  6-8 mm diameter, 20-200 mm length
- Xpert Self-expanding Peripheral Stent (Medtronic)
  4-6 mm diameter, 40-80 mm length

Table 7: List of material, treatments or diagnostic agents

9.1.1 Experimental Intervention

The experimental device is a commercially available (CE-certified) product used routinely at the study center and in several European countries. The device is a sirolimus-coated balloon catheter that delivers the immunosuppressant sirolimus via nano drug carriers (phospholipids) into the target vessel wall. It will be used in arteries below the inguinal ligament and after vessel preparation with uncoated balloon catheters (see 9.2.1). The catheter itself will not remain in-situ.

The Magic Touch PTA device is a Sirolimus coated balloon catheter indicated for treatment of stenotic lesions of infra-inguinal or infra-popliteal arteries. The drug dose per mm^2 balloon surface is 1.27 µg.

The Magic Touch Sirolimus coated balloon catheter is considered a drug/device combination product and consists of two components: (1) a PTA balloon catheter coated with (2) polymer free formulation containing the Sirolimus drug as an active ingredient in an encapsulated phospholipid excipient.

The balloon is designed to reach specific diameters at specific pressures. In order to correctly position the balloon under fluoroscopy, two radiopaque markers are located on the shaft under the balloon itself, defining its cylindrical area. The catheter includes a smooth, soft and atraumatic tip to facilitate advancement of the catheter through the stenosis, in addition to a hydrophilic coating present on the distal shaft.

Provisional stent placement

Provisional stent placement with commercially available (CE-certified) bare metal stents may be used in case of severe arterial dissection and/or persisting (flow-limiting) lumen compromise following balloon angiography necessitating a stent for mechanical scaffold. Bare metal stents that will be used include, but are not limited to EverFlex Self-expanding Peripheral Stent (Medtronic) 6-8 mm diameter, 20-200 mm length.

9.1.2 Control Intervention

All commercially available (CE-certified) uncoated balloon-catheters with application in peripheral arterial disease approved for inpatient use in Switzerland will function as control devices. All devices will derive from routine hospital storage. The control device(s) will be used in arteries below the inguinal ligament. The catheter itself will not remain in-situ.

Balloon catheters use include, but are not limited to

- Mustang 0.035'' Balloon Dilatation Catheter (Boston scientific)
  3-12 mm diameter, 20-200 mm length
- Sterling 0.018'' Balloon Dilatation Catheter (Boston scientific)
  2-3 mm diameter, 20-220 mm length
- Coyote 0.014''Balloon Dilatation Catheter (Boston scientific)
  1-4 mm diameter, 10-200 mm length

Provisional stent placement with commercially available (CE-certified) bare metal stents may be used in case of severe arterial dissection and/or persisting (flow-limiting) lumen compromise following balloon angiography necessitating a stent for mechanical scaffold.

Bare metal stents that will be used include, but are not limited to

- Life Self expanding Peripheral Stent (Bard)
  5-8 mm diameter, 20-200 mm length
- EverFlex Self-expanding Peripheral Stent (Medtronic)
  6-8 mm diameter, 20-200 mm length
- Xpert Self-expanding Peripheral Stent (Medtronic)
  4-6 mm diameter, 40-80 mm length

9.1.3 Packaging, Labelling and Supply (Re-Supply)

The interventional and control devices are used in clinical routine and belong to our own storage. Therefore, no specific packaging, labelling or supply will be necessary. The sponsor-investigator will ensure sufficient re-supply of devices and identify those used in patients enrolled in the study for safety, administrative, and economic (reimbursement of the devices) reasons: all material used (balloon catheters, stents) in this study, will be documented in the eCRF using the commercial name, diameter (mm), length (mm), and batch number.

9.1.4 Storage Conditions

Since the study (and control) devices are used in clinical routine and derive from our own storage, there are no special handling requirements. Study (and control) devices will be stored according to the requirements of the manufacturer and hospital in a separate room with limited access (investigators, interventionalists, medical technicians). No specific handling requirements apply.

9.2 Administration of Experimental and Control Interventions

9.2.1 Experimental Intervention

Upon angiographic determination of the target lesion, primary plain old balloon angioplasty (POBA, uncoated) will be performed for vessel preparation (pre-dilatation) using a balloon diameter corresponding to the reference vessel diameter. In a second step, patients will receive target lesion treatment with the MagicTouch PTA sirolimus coated balloon (Concept Medical B.V., Hoevelaken, The Netherlands). All balloons will be inflated for a duration of 120 seconds at nominal pressure. Nominal pressure is defined as the inflation pressure required to reach the device-specific diameter.

Provisional stent placement will be performed at the discretion of the interventionist.

<u>For primary POBA:</u>
Any available CE-certified uncoated balloon catheter with application in peripheral artery disease approved for in-patient use in Switzerland.

<u>For provisional stent placement</u>:
Any available CE-certified uncoated vascular stent with application in peripheral arterial disease approved for inpatient use in Switzerland.

9.2.2 Control Intervention

Upon angiographic determination of the target lesion, plain old balloon angioplasty (POBA, uncoated) will be performed using a balloon diameter corresponding to the reference vessel diameter. All balloons will be inflated for a duration of 120 seconds at nominal pressure. Nominal pressure is defined as the inflation pressure required to reach the device-specific diameter.

Provisional stent placement will be performed at the discretion of the interventionist.

<u>For POBA:</u>
Any available CE-certified uncoated balloon catheter with application in peripheral artery disease approved for in-patient use in Switzerland.

<u>For provisional stent placement</u>:
Any available CE-certified uncoated vascular stent with application in peripheral arterial disease approved for inpatient use in Switzerland.

9.3 Modifications of Device Application

Modifying allocated intervention for a participant requesting a certain treatment regimen is not planned. Patients who request a certain strategy (uncoated or sirolimus-coated) for specific reasons will be classified as a screening failure if not randomized in the study.

9.4 Compliance with Study Intervention

Since the treatment period ends with the termination of the re-vascularization procedure, and the investigational product does not remain is situ, there is no need to track participant compliance. The compliance of the interventionist (investigator) to the use of the respective study devices will be monitored. The use of a drug-coated balloon catheter in a patient allocated to the uncoated group and vice versa, or the use of any drug-eluting stents will be documented as a major protocol violation.

9.5 Data Collection and Follow-up for Withdrawn Participants

This study will utilize an electronic database and eCRF. In order to accurately collect all information, subject worksheets will be provided for study specific data (specific operative data points, etc.) not found directly available from in the medical records or in the online medical charts: these and will be considered as paper source document. All data requested on the eCRF are considered required. Data points not collected and/or recorded will be considered deviations unless otherwise specified. The Principal Investigator must ensure the accuracy and completeness of the recorded data and then provide his/her electronic signature on the appropriate eCRFs. The Investigator's electronic signature for specific eCRFs will be documented in compliance with local regulations. Changes to data previously submitted to the sponsor will require a new electronic signature by the Investigator to acknowledge/approve the changes. All data generated until time of withdrawal will be used. Follow-up of withdrawn participants will still include vital registration data at 365 and 630 days.

9.6 Trial Specific Preventive Measures

None deviating from the standard of care of patients with PAD.

9.7 Concomitant Interventions

Provisional stent placement is used in case of severe arterial dissection and/or persisting (flow-limiting) lumen compromise following balloon angiography necessitating a stent for mechanical scaffold. Concomitant in- and outflow disease may be treated upon discretion of the interventionist using uncoated devices. All concomitant treatment have to be recorded in the CRF.

9.8 Medical Device Accountability

The investigational product will be stored in secure storage area (locked room next to the catheter laboratory) with limited access of only the investigators, and nurses working at the catheter laboratory. All information for the use, storage, and handling of the investigational device will be taken into full account according to the Instruction for Use.

9.9 Return or Destruction of Medical Device

Return or destruction medical device does not apply, since medical devices will be used from our own inventory.

10 STUDY ASSESSMENTS

10.1 Study Flow Chart(s)/Table of Study Procedures and Assessments

**Procedures**	**Clinical screening**	**Angiography screening, randomization, and treatment (baseline)**		**Clinical follow-up and assessment of the vital status**		
**Visit**	**V1**	**V2**	**V3**	**V4**	**V5**	**V6-V7**
**Time**	**Day -90 to 0**	**Day 1**	**Discharge from hospital**	**Day 180±30**	**Day 365 (±21)[2]**	**Year 2-Year 5[2]**
Patient Information and Informed Consent for screening and participation	X					
Ultrasonography or evaluation of prior echographic findings	(X)	(X)				
Staging (Fontaine, Rutherford)	X			(X)[1]	(X)[1]	
Collection of personal information (demographics, risk factors, medical history)	X					
Decision about planned amputation	X					
Vital signs	X	X	X			
Vital status				(X)[1]	(X)[1]	(X)[1]
Laboratory tests (routine)	X	(X)				
Evaluation of information concerning prior endovascular/surgical procedures	X					
Verification of the eligibility criteria	X	X				
Definition of target lesion		X				
Randomization and allocation		X				
Intervention		X				
Primary and secondary efficacy outcomes assessment			X	X[1]	X[1]	
Primary safety outcome assessment (death)		X	X	X[1]	X[1]	X
Assessment of (S)AEs and secondary safety outcomes		X	X	X	X	
Assessment of antithrombotic and lipid lowering medication			X	X	X	
**Type of contact**	In-hospital	In-hospital	In-hospital	In-hospital[1] or teleph.; (national vital registry)	In-hospital[1] or teleph.; (national vital registry)	National vital registry; (teleph.)
[1]Follow-up visits will be performed during routine visits at our outpatient clinics (hub and satellites). Patients who are not routinely follow-up in our outpatient clinics will be interviewed by telephone, or information will be gathered by contacting relatives. In addition, we may collect follow-up reports from external vascular specialists and the general practitioners. [2]Since vital registration databases may not be updated on a daily basis, information concerning the vital status will be collected at Day 365 +/- 21, Year 2, or Year 5; the vital status reported in the eCRF will refer to that on Year 1, 2, and 5 will be recorded.						

Table 8: Flow chart

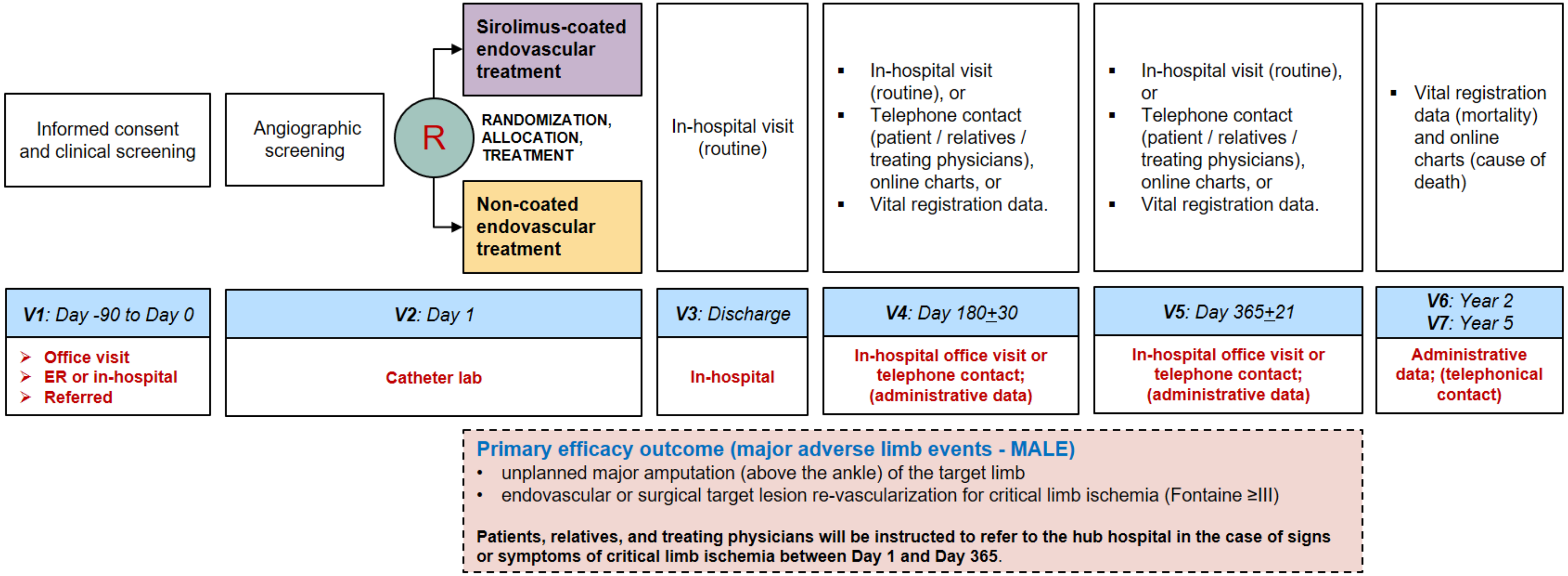

Figure 7: Study flow and summary of the study procedures

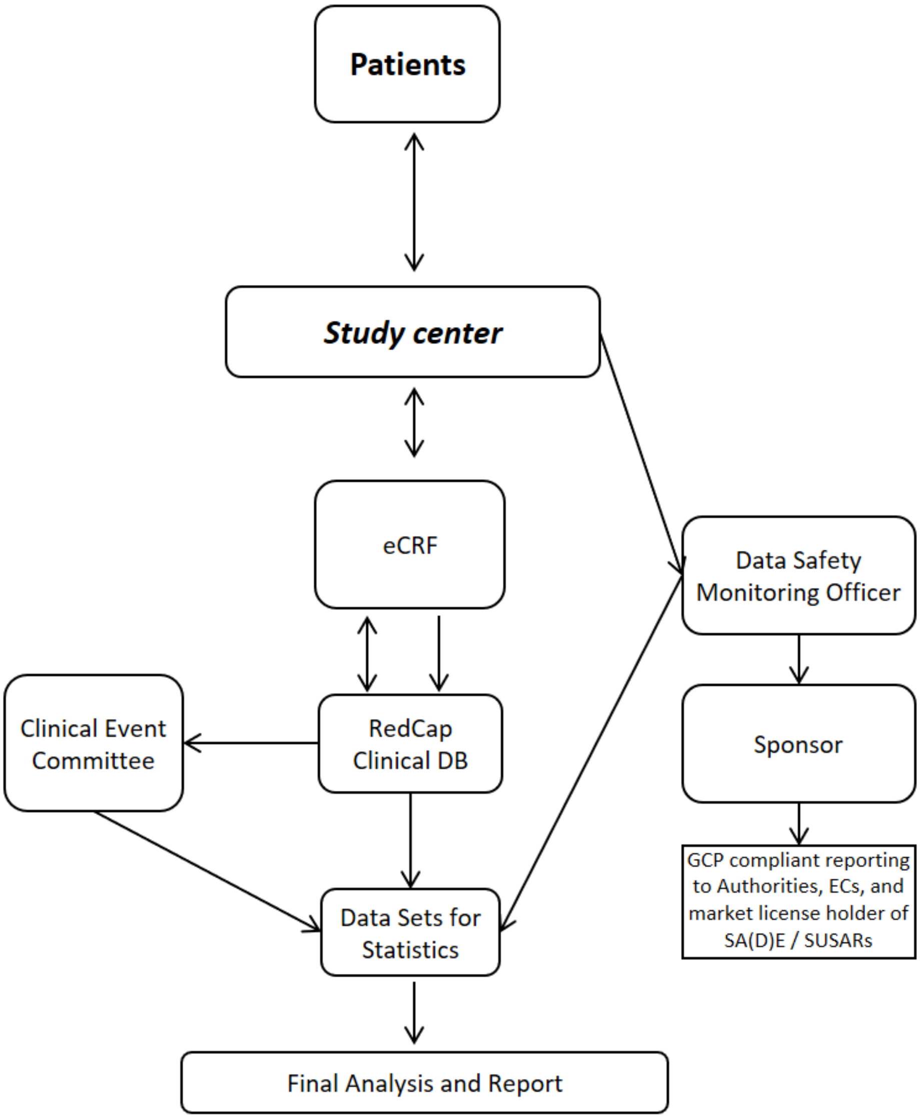

Figure 8: Data collection and data flow.

10.2 Assessments of Outcomes

10.2.1 Assessment of Primary Outcome

For the primary analysis, the primary efficacy outcome will be assessed by the physicians within one year from randomization. Formal assessment of the primary outcome will be in place at two additional time points, including hospital discharge and first follow-up visit (Day 180).

All patients will be instructed to return to the hospital as soon as new symptoms occur, at which time objective tests and clinical examination will be performed.

The primary efficacy outcome will be initially verified on the day of discharge from hospital. After discharge, if the patient is followed at the study center (Clinic of Angiology, University of Zürich), a

routine follow-up visit will be performed between Day 180 after randomization. In case a patient is not followed at the study center (Clinic of Angiology, University of Zürich), the treating physicians, the patient, and the relatives will be instructed to contact the study investigators if any of the primary outcomes are suspected.

If a patient will not undergo or will not be able to attend routine in-hospital visit, or if no information will be spontaneously obtained from the treating physicians at Day 180, the investigators will
(1) verify whether the patient has been recently admitted in another department of the study center,
(2) contact the patient or the relatives (phone contact),
(3) verify the survival status.

The phone interviews will be performed by specially trained staff of the University of Zürich and will be based on a standardized list of questions integrated in the eCRF.

This strategy will allow minimal burden for the patients, who will not undergo additional visits that are not part of routine clinical and ultrasonographic follow-up. In particular, given the ´hard´ nature of the primary efficacy outcome, the ease of assessment and the fact that these patients are usually referred to the center where the intervention was performed, the risk of reporting bias would be minimal.

An internal Clinical Events Committee made up of two clinicians will categorize the components of the primary outcome. At the onset of the trial, the committee will establish explicit rules outlining the minimum amount of data required in order to classify a clinical event. The Clinical Events Committee will meet regularly to review and adjudicate all clinical events from all study centers.

10.2.2 Assessment of the Components of the Secondary Outcomes

The same procedures planned for the assessment of the primary efficacy outcome will also apply for the secondary outcomes. In particular, the following outcomes will be assessed:

- Unplanned minor amputation at target limb at Day 180 and at Day 365;
- Unplanned major amputation at target limb at Day 180 and at Day 365;
- Any target lesion re-vascularization at Day 180 and at Day 365;
- Unplanned target lesion re-vascularization for non-critical limb ischemia at Day 180 and at Day 365;
- Target lesion re-vascularization for critical limb ischemia at Day 180 and at Day 365;
- Unplanned target limb re-vascularization at Day 180 and at Day 365.

10.2.3 Assessment of Safety Outcomes

10.2.3.1 Adverse Events

Survival status and the cause of death will be assessed during follow-up if a patient misses a scheduled visit or cannot be contacted telephonically, as well as at the end of follow-up (Year 1) and at the time of long-term follow-up (Year 2 and Year 5).
In the case a patient is not followed at the study center (Clinic of Angiology, University of Zürich), the treating physicians, the patient, and the relatives will be instructed to contact the study investigators if a severe adverse event is suspected. All patients will be instructed to return to the hospital as soon as new symptoms occur, at which time information about the time of onset, intensity, and duration of the event will be collected, and objective tests and clinical examination may be performed.

The Data Safety Monitoring committee is responsible for the oversight and safety monitoring of the study. Regular evaluations of safety data will be after inclusion of multiples of 150 patients. The committee advises the Sponsor regarding the continuing safety of the trial subjects.

10.2.3.2 Laboratory Parameters

No laboratory parameters deviating from routine will be measured during the screening phase and follow-up.

10.2.3.3 Vital Signs

Vital signs measurements are part of the clinical routine. Vital signs will be assessed during the screening phase and in concomitance of in-hospital visits according with standard operating procedures in order to guarantee reproducibility. After 5 min of resting in the supine position, blood pressure (systolic and diastolic) will be measured. To obtain occlusion pressures of the anterior and posterior tibial artery, cuffs will be applied to the calf and inflated 30-50 mmHg above the systolic blood pressure. While deflating, the pulse (and corresponding occlusion pressures) at the level of the ankle will be measured using a hand-held bidirectional Doppler probe.

10.2.4 Assessments in Participants Who Prematurely Stop the Study

The dropout rate in SirPAD is estimated to be 5% and the sample size was adjusted to account for 5% dropouts. Patients who prematurely stop the study will not be replaced. Adverse events until the time of drop out will be kept on file but no further assessments are planned. All the participants who prematurely stop the study will be followed as per standard practice in the case an adverse event occurs. If the study is stopped between Day 180 and Day 365, the vital status of all patients will be assessed after 1, 2, and 5 years.

10.3 Procedures at Each Visit

10.3.1 Visit 1 or Clinical Screening Visit

Checking inclusion and exclusion criteria and scheduling an angiography exam:

- Collection of patient’s written informed consent for screening and participation in the study;
- Clinical assessment, including ultrasonography or evaluation of prior imaging (optional), staging, collection of demographic data, risk factors and medical history including concomitant diseases and medication;
- Clinical assessment whether decision about planned amputation has been made;
- Physical examination, including vital signs (systolic and diastolic blood pressure) and staging of disease (Fontaine, Rutherford);
- Evaluation of previous laboratory data and routine laboratory screening, including hemoglobin and peripheral cell count, renal function, coagulation parameters;
- Collection of information concerning prior interventional report;
- Verification of the eligibility criteria.

10.3.2 Visit 2 or Angiography Screening and Baseline Visit

- Clinical assessment including ultrasonography or evaluation of prior imaging (optional);
- Vital signs: systolic and diastolic blood pressure;
- Evaluation of previous laboratory data and routine laboratory screening, including hemoglobin and peripheral cell count, renal function, coagulation parameters (option);
- Verification of the eligibility criteria;
- Angiography screening including definition of target lesion;
- Randomization and treatment allocation;
- Intervention;
- Assessment of safety outcomes.

10.3.3 Visit 3 or Discharge

- Vital signs: systolic and diastolic blood pressure;
- Assessment of primary and secondary efficacy outcomes;

- Assessment of efficacy and safety outcomes;
- Assessment of new concomitant medications (antithrombotic and lipid lowering therapy).

10.3.4 Visit 4 or Day 180 (± 30) Visit

In-hospital routine visit or phone contacts or national vital registry;

- Staging of disease (Fontaine, Rutherford);
- Assessment of efficacy and safety outcomes;
- Ultrasonography (if available and routinely performed) and physical examination including vital signs (systolic and diastolic blood pressure) if the patient is visited in-hospital.

Of note, if the routine visit was performed in a clinic not related to the study center, assessment will be performed based on accessible clinical reports. If no routine follow-up was performed and neither the patient nor relatives can be contacted, national vital registration data will be assessed for safety outcomes.

10.3.1 Visit 5 or Day 365 (± 21) Visit

In-hospital routine visit or phone contacts or national vital registry;

- Staging of disease (Fontaine, Rutherford);
- Assessment of efficacy and safety outcomes;
- Ultrasonography (if available and routinely performed) and physical examination including vital signs (systolic and diastolic blood pressure) if the patient is visited in-hospital.

Of note, if the routine visit was performed in a clinic not related to the study center, assessment will be performed based on accessible clinical reports. If no routine follow-up was performed and neither the patient nor relatives can be contacted, national vital registration data will be assessed for safety outcomes.

10.3.2 Year-2 and Year-5 Assessment (Vital Status)

Phone contact or national vital registry;

- Assessment of survival or cause of death.

If neither the patient nor relatives can be contacted, national registration data will be assessed for the outcome.

11 SAFETY

The Sponsor's SOPs provide more detail on safety reporting. During the entire duration of the study, all adverse events (AEs), serious adverse events (SAEs) and incidents are to be collected, fully investigated and documented in source documents and case report forms (CRF). Study duration encompasses the time from when the participant signs the informed consent until the last investigation plan-specific procedure has been completed, including a safety follow-up period.

11.1 Definition of (Serious) Adverse Events and Other Safety Related Events

Adverse Event (AE)
PAD patients often have multiple comorbidities requiring medical attention. Non-cardiovascular adverse events will not be systematically documented because there are no known adverse effects from local vascular application of sirolimus to organs and tissue. Any untoward cardiovascular occurrence, unintended cardiovascular disease or injury or any untoward clinical signs of cardiovascular disease whether or not related to the investigational medical device will be documented. This definition includes events related to the investigational device or the comparator. This definition includes events related to the procedures involved. For users or other persons, this definition is restricted to events related to investigational medical devices. Adverse events will be collected starting from randomization to discharge from hospital. All enrolled subjects will be evaluated for Adverse Events.

Serious Adverse Event (SAE) (Art. 2 Abs 58 MDR)
Any adverse event that led to any of the following:
(a) death,
(b) serious deterioration in the health of the subject that resulted in any of the following:
(i) life-threatening illness or injury,
(ii) permanent impairment of a body structure or a body function,
(iii) hospitalisation or prolongation of patient hospitalisation,
(iv) medical or surgical intervention to prevent life-threatening illness or injury or permanent impairment to a body structure or a body function,
(v) chronic disease,
(c) foetal distress, foetal death or a congenital physical or mental impairment or birth defect.
Note: planned hospitalization for pre-existing condition, or a procedure required by the CIP, without a serious deterioration of the health status of the subject, is not considered an SAE.

Device deficiency (Art. 2 Abs 59 MDR)
Inadequacy of a medical device related to its identity, quality, durability, reliability, safety or performance, of an investigational device, including malfunction, user errors and inadequate information supplied by the manufacturer.

Malfunction (ISO14155)
Failure of an investigational device to perform in accordance with its intended purpose when used in accordance with the instructions for use or the CIP.

Device deficiency with Serious Adverse Device Effect (SADE) potential (Art. 80 Abs 1 letter c MDR; ISO14155)
Any device deficiency that might have led to a serious adverse event if appropriate action had not been taken, intervention had not occurred, or circumstances had been less fortunate.

Adverse Device Effect (ADE) (ISO14155)
Adverse event possibly, probably or causally related to the use of an investigational device or procedures.

Serious Adverse Device Effect (SADE) (ISO14155)
Adverse device effect (ADE) that has resulted in any of the consequences characteristic of a serious adverse event.

Unanticipated Serious Adverse Device Effect (USADE) (ISO14155)
Serious adverse device effect (SADE) which by its nature, incidence, severity or outcome has not been identified in the current version of the risk analysis report.

Causal Relationship of SAE (MDCG 2020-10/1)
A causal relationship towards the medical device or the procedure of the investigation should be rated by the PI and the Sponsor as follows:
Not related: The relationship to the device or procedures can be excluded.
Possible: The relationship with the use of the investigational device is weak but cannot be ruled out completely. Alternative causes are also possible.
Probable: The relationship with the use of the investigational device seems relevant and/or the event cannot reasonably be explained by another cause.
Causal relationship: The serious event is associated with the investigational device or with procedures beyond reasonable doubt.

11.2 Categorization

The adverse events are categorized by the PI and the Sponsor using the following algorithm:

Does the AE meet the seriousness criteria?

- No, it is not serious
  - Is the relationship to the device or the procedure possible, probable or causal?
    - No: non-related AE
    - Yes: ADE
- Yes, it is serious: SAE
  - Is the relationship to the device or the procedure possible, probable or causal?
    - No: non-related SAE
    - Yes: SADE
- Is it anticipated (within expected type, severity and frequency of the complications)?
    - No: unanticipated SADE (USADE)
    - Yes: anticipated SADE (ASADE)

11.3 Recording of Serious Adverse Events and Other Safety Related Events

Clinical study participants will be routinely questioned about AEs at study visits. The well-being of the participants will be ascertained by neutral questioning, ("How are you?"). The investigator is responsible for reporting all SAEs occurring during the course of the study.

All observed or volunteered SAEs or serious adverse device effects, regardless of treatment group or suspected causal relationship to the investigational device or study related procedure will be recorded in the patient file and subsequently in the eCRF. For each SAE, the investigator will provide the onset, duration, intensity, treatment required, outcome and action taken with the investigational device or study related procedure.

SAEs or abnormal test findings felt to be associated with the investigational device or study procedures will be followed until the event (or its sequelae) or the abnormal test finding resolves or stabilizes at a level acceptable to the investigator.

The investigator and the sponsor have both (if the investigator is not the same person as the sponsor) to categorize the AEs independently.

11.4 Reporting of Serious Adverse Events and Other Safety Related Events

Reporting to the Sponsor:

All SAEs, device deficiencies and health hazards that require measures are reported to the Sponsor by the PI (or authorized designee) within 24 hours after becoming aware of the event. Device deficiencies are assessed regarding their potential to lead to an SAE. DD are assessed regarding their potential to lead to an SAE. The other study site (Fribourg) will report on SAE via RedCAP and the Sponsor will receive automatic email notification with the possibility of logging in and access the original study documents and clinical information, as provided by the investigators.

Reporting to the Competent Ethics Committee:
The Sponsor reports to the CEC promptly any serious adverse event which has a causal relation with the MD, comparator or procedure/test method or where a causal relation appears to be possible (Art. 33 ClinO-MD).
In order to ensure prompt notification, the Sponsor may initially submit an incomplete notification.
If safety and health hazards that require measures must be taken immediately during the conduct of the investigation, the Sponsor notifies the CEC within 2 days of these measures and the circumstances which made them necessary (Art. 34 ClinO-MD).

Periodic safety reporting (Art. 35 ClinO-MD):
An Annual Safety Report (ASR) is submitted by the Sponsor to the CEC, yearly. The ASR contains a list of all SADEs and DDs and a report on their degree of seriousness, causal relationship with the MD and procedure and on subjects' safety.

Other reporting is done according to provisions of MD vigilance as per Art. 87-90 MDR (Art. 33 abs 4.b ClinO-MD) and Art. 67 MedDO.

11.5 Follow up of Serious Adverse Events

Participants terminating the study (either regularly or prematurely) with

- reported ongoing SAE, or
- any ongoing SAEs of laboratory values or of vital signs being beyond the alert limit

will be followed with an unplanned follow-up investigation: this visit will take place up to 30 days after terminating the treatment period. Follow-up information on the outcome will be recorded on the respective AE page in the CRF/eCRF. All other information must be documented in the source documents. Source data has to be available upon request.

In case of participants lost to follow-up, efforts should be made and documented to contact the participant to encourage him/her to continue study participation as scheduled.

Pregnant patients will be withdrawn from the study and the pregnancy will be followed, particularly concerning the use of concomitant medications and potential detrimental effects related to the release of sirolimus. Pregnancy will be notified to the Sponsor-Investigator within a maximum of 24 hours.

Follow-up investigations may also be necessary according to the investigator's medical judgment even if the participant has no AE at the end of the study. However, information related to these investigations does not have to be documented in the eCRF but must be noted in the source documents.

12 STATISTICAL METHODS

12.1 Hypothesis

The null hypothesis of this study states that the event rate (of MALE) in the experimental group () is higher than the event rate in the control group (uncoated balloon catheter).

The alternative hypothesis states that the event rate in the experimental group is non-inferior, as compared

to the control group. The rates of MALE events are expected to be 10% within one year in both groups and the non-inferiority margin is set at +5%.

If the null hypothesis concerning the primary objective is rejected (and the primary objective, non-inferiority, is thus established), further confirmatory statistical tests for superiority concerning primary and secondary endpoints will be performed using a prespecified hierarchical order based on the clinical importance of the considered outcomes.

12.2 Determination of Sample Size

Assuming a 10% event rate (MALE) within 12 months in both the control and intervention group, and a non-inferiority margin of 5% expressed as absolute risk difference, a total of 1132 patients (566 patients per treatment group) allow to show non-inferiority of the intervention group with a power of 80% and a type I error rate of α=2.5% one-sided.

Assuming a drop-out rate of 5%, including randomization failures, a total of 1200 patients will be randomized in the study.

The sample size calculation is based on previous studies reporting MALE rates in PAD patients after re-vascularization procedures. These studies were heterogeneous in study design, patient selection, techniques (proportion of patients with endovascular approach) and findings.[19,21-24] *Hess et al.* reported a MALE incidence of 10% at 12 months among 381.415 re-vascularized patients that were included in the Premier Healthcare Database between April 2009 and September 2014.[19] In addition, there were two larger interventional studies reporting 30-day MALE incidence after surgical or endovascular therapy of symptomatic PAD patients. *Fashandi et al.* reported a MALE incidence of 3.2% in patients with claudication at 1 months following therapy.[21] *Mehaffey et al.* reported a MALE incidence of 12.2% in patients with critical limb ischemia (CLI).[22] The COMPASS trial estimated a MALE incidence of 2.0% among 6.341 patients with PAD at 21 months, of which 35% were asymptomatic. The MALE incidence was 3.6% among the subgroup of patients with previous re-vascularization procedures.[24] A subgroup analysis of the Fourier trial estimated a MALE incidence of 1.5% among 3.642 patients with PAD (31% asymptomatic) at 12 months.[23] In the XTOSI study, the 6-month amputation free survival was 90% in patients receiving sirolimus-coated balloon catheters.

12.3 Statistical Criteria of Termination of Trial

No statistical criteria for termination of the trial are specified.

12.4 Planned Analyses

The primary outcome, number of MALE events at 12 months follows a binomial distribution. The proportion (risk) for MALE in both treatment groups will be estimated with 95% confidence intervals and the risk difference between treatment groups will be calculated again with a confidence interval. To preserve the overall type I error for one interim analysis and the final analysis, the α of 0.05 (2-sided) is spent using the Lan-DeMets spending function. At interim analysis, a 99.95% confidence interval for the risk difference is calculated, at final analysis, a 95.05% confidence interval is calculated.

In a secondary analysis, time to MALE event will be addressed with the Kaplan-Meier method and a Cox proportional hazards model, resulting in an estimated hazard ratio with 95% confidence interval for between-group comparison. The Cox model may be extended to include baseline characteristics and stratification variables in order to increase the precision of the treatment effect estimate.

Secondary outcomes death and clinically driven TLR, will be addressed accordingly.

Logistic regression for the outcome MALE event will be used to estimate an adjusted treatment effect, given the stratification variables.

Descriptive statistics will include mean and standard deviation for continuous parameters, as well as number and percentage of total for categorical parameters. Descriptive statistics will be displayed separately for each treatment group.

A detailed statistical analysis plan for interim and final analysis will be written after finalization of investigation plan, following the corresponding guideline.[25]

12.4.1 Datasets to be Analyzed, Analysis Populations

In this non-inferiority trial, the primary efficacy outcome will be compared in the intention-to-treat, as-treated, and per-protocol populations; the results of these analyses will be examined for consistency.

The *intention-to-treat population* will include all subjects randomized according to randomized treatment assignment, therefore ignoring protocol deviations, withdrawal, and anything that happens after randomization.

The *as-treated population* will include only those subjects treated with either sirolimus-coated or uncoated catheter, and the comparison will be based on the actual device used. We anticipate that the as-treated population will not deviate from the intention-to-treat population given the short time (minutes) between randomization/allocation and treatment.

The *per-protocol population* will be composed of all patients with appropriated exposure to treatment, complete follow-up, and absence of major protocol violations, as hereby defined:

- Assigned treatment given;
- Correct predilatation;
- No major violations of the inclusion criteria, including the absence of peripheral artery disease below the inguinal ligament;
- Available follow-up data for the primary outcome.

The following subgroups are pre-specified for analysis of heterogeneity of effects: Age $\geq$ 75 vs. <75, men vs. women, patients with elective re-vascularization (Fontaine I-II) vs critical re-vascularization (Fontaine >II), patients with 1 vs. more than 1 level intervention, de novo lesion vs. re-stenosed, total occlusion vs. partial occlusion, distal vs. proximal lesions. If there is evidence for a differential treatment effect within subgroups (i.e. p-value of interaction test < 0.05), the treatment effect will be reported within subgroups as odds ratios or hazard ratios.

12.4.2 Primary Analysis

In the primary analysis of this trial, the absolute risk difference for MALE at 12 months follow-up between treatment groups will be estimated, together with its 2-sided 95% confidence interval. The trial statistician will perform the analyses after termination of the trial. If the overall event rate estimated at interim analysis is > 10%, an unblinded estimation of the risk difference and confidence interval will be calculated. The confidence level α will then be spent such that the overall type I error is preserved for an interim analysis and a final analysis, using a Lan-DeMets spending function. At interim, the confidence level will therefore be 99.95% and for final analysis, it will be 95.05%.

If the null hypothesis concerning the primary objective is rejected (and the primary objective, non-inferiority, is thus established), further confirmatory statistical tests on primary and secondary endpoints will be performed using a prespecified hierarchical order based on the clinical importance of the considered outcomes:

1. superiority for the composite of unplanned (major or minor) index-limb amputations or any target lesion re-vascularization within 365 days after enrolment (α=2.5% one-sided);
2. superiority for MALE within 365 days after enrolment (α=2.5% one-sided).

No additional reduction or splitting of the single α levels is necessary for this reason since the pre-defined ordering avoids any choice in the assessment (Guideline on multiplicity issues in clinical trials, European Medicines Agency; EMA/CHMP/44762/2017).

12.4.3 Secondary Analyses

Secondary analyses include time-to-event analyses of MALE, estimation of hazard rates in both treatment groups and estimation of a hazard ratio with 95% confidence interval. Cox proportional hazards model will be applied. Pre-specified subgroup analyses will be performed only if the corresponding interaction test showed evidence for a differential treatment effect between subgroups ($p<0.05$). The null hypothesis of interaction tests is that there is no differential treatment effect between subgroups. The alternative hypothesis states that there is a differential treatment effect between subgroups. If subgroup analyses are performed, the resulting odds ratios or hazard ratios will be reported including 95% confidence intervals. The trial statistician will perform the analyses after termination of the trial.

12.4.4 Interim efficacy and safety Analyses

A single interim analysis with information rate of 50%, i.e. 300 patients per arm, 600 in total is planned. If at interim the overall event rate exceeds a pre-specified threshold of 10% the event rate will be estimated in both treatment groups (unblinded).
Following *D'Agostino et al.*[26] "for efficacy reasons one can argue that there is no real ethical issue with seeing the [non-inferiority] trial to completion from an efficacy perspective". However, "interim analyses are also important in a non-inferiority trial for safety reasons, either to ensure the experimental treatment is not doing more harm than good (R1), or that it is superior with regard to specific adverse events (R2)".

Monitoring for efficacy:
If the overall event rate at interim analysis is higher than the threshold of 10%, the event rate in both treatment groups will be estimated and the between group risk difference (RD) and its confidence interval will be estimated. To preserve the overall type I error for one interim analysis and the final analysis, the α of 0.05 (2-sided) is spent using the Lan-DeMets spending function. At interim analysis, a 99.95% confidence interval is calculated, at final analysis, a 95.05% confidence interval is calculated. It is unlikely that at interim analysis, non-inferiority can be declared unless the experimental treatment is superior to active control.

Monitoring for safety:
Safety concerns in this study are two-fold: (R1) the efficacy endpoint (MALE) may differ substantially between treatment groups and (R2) mortality may differ between treatment groups. On safety grounds, termination of the trial may be recommended after interim analysis. However, such assessments may potentially have implications for falsely concluding that there is an adverse effect.

No statistical reasons for stopping the trial at interim analysis are declared.

Notation:
δ_{Int} : between group risk difference p_{exp} - p_{con} at 12 months at interim analysis for MALE
d_{Int} : between group risk difference π_{exp} - π_{con} at 12 months at interim analysis for mortality
exp = experimental group, coated devices
con = active control group, uncoated devices

Scenario	Reasoning	Interpretation	Action
Interim analysis after 600 patients followed up 12 months (50% of total sample size)			
$p_{total} \leq 0.10$	Verify assumptions about nuisance parameters.	Obtain estimate of overall MALE event rate at interim.<br>Assumptions about nuisance parameters seem to be realistic.	Continue trial without any changes.
$p_{total} > 0.10$	Safety	Differential MALE event rates between treatment groups could be causing higher event rate than anticipated.	Unblinded estimation of the MALE event rates in each treatment group.
(1) $\delta_{Int} = 0$	Efficacy	$p_{exp} = p_{con}$	Calculate Bayesian predictive probabilities for successful termination

			of trial. Continue the trial unless otherwise stated by DMB. Based on interim results, the DMB may suggest a sample size increase.
(2) $\delta_{Int} > 0$	Efficacy: R1	MALE event rate in experimental group is considerably higher than in control group.	Quantification of "how much higher". DMB discusses stopping for safety reasons.
(3) $\delta_{Int} < 0$	Efficacy: R1	MALE event rate in experimental group is considerably lower than in control group.	Quantification of "how much lower". DMB discusses stopping for safety reasons.

Continuous safety evaluation, after multiples of 150 patients at 12 months follow-up			
$d_{Int} \neq 0$	Safety: R2	Unblinded estimation of mortality rates. Assess if mortality rates are different between treatment groups.	How much higher mortality rate in one group compared to the other after 12 months follow-up?

Table 9: Statistical scenarios

These guidelines are non-binding for the DMB.

12.4.5 Deviation(s) from the Original Statistical Plan

Any deviation from the original statistical plan will be addressed in an amendment of the study protocol, which will undergo formal reevaluation similarly to other protocol amendments.

12.5 Handling of Missing Data and Drop-Outs

Since the study will be implemented with an all-comer design, some degree of missing data and dropouts are expected. In order to limit the amount of missing data points, we have implemented a sequential system to optimize data collection: i) Information will be collected at the time of the planned visit; ii) telephone contacts of patients / relatives / treating physicians; iii) education of patients to refer to the study center if any new symptoms occur; iv) assessment of online medical charts and 'Zivilstand'. Patients who discontinue the clinical trial participation prematurely after randomization will not be replaced. Patients who drop out before randomization will be replaced.
For the analysis of outcomes with missing data, the missingness generating mechanism will be evaluated and multiple imputation techniques will be applied.

13 ELIGIBILITY OF THE PROJECT SITE

As a tertiary clinic for angiology, the project site is a highly specialized consultative health care provider for both in- and outpatients in the Zurich area. The clinic for angiology has a 24-hour emergency service including catheterization laboratory standby, and performs approximately 800 peripheral intervention per year.

14 DATA QUALITY ASSURANCE AND CONTROL

The Sponsor-Investigator is implementing and maintaining quality assurance and quality control systems with written SOPs and Working Instructions to ensure that trials are conducted and data are generated, documented (record), and reported in compliance with the protocol, ISO 14155, and applicable regulatory requirement(s). Monitoring will be conducted during the course of the study for quality assurance purposes.

14.1 Data Handling and Record Keeping / Archiving

The study will strictly follow the investigation plan. If any changes become necessary, they must be laid down in an amendment to the investigation plan. All amendments of the investigation plan must be signed by the Sponsor-Investigator and submitted to Competent Ethic Committee.

14.1.1 Case Report Forms

The investigators will use electronic case report forms (eCRF), one for each enrolled study participant, to be filled in with all relevant data pertaining to the participant during the study. The investigator will document the participation of each study participant on the Enrolment Log.

All participants who were considered eligible but not enrolled into the study will be listed into the Screening Failure Log, which will be prepared in accordance with the CONSORT guidelines (see 8.3).

For data and query management, monitoring, reporting and coding an internet-based secure data base REDCap developed in agreement to the Good Clinical Practice (GCP) guidelines provided by the Clinical Trials Center (CTC) Zurich will be used for this study. It is the responsibility of the investigator to assure that all data in the course of the study will be entered completely and correctly in the respective database. Corrections in the eCRF may only be done by the investigator or by other authorized persons. In case of corrections, the original data entries will be archived in the system and can be made visible. For all data entries and corrections date, time of day and person who is performing the entries will be generated automatically. eCRFs must be kept current to reflect participant status at each phase during the course of study. Participants must not to be identified in the eCRF by name. Appropriate coded identification (e.g. Participant Number) must be used. It must be assured that any authorized person, who may perform data entries and changes in the eCRF, can be identified. A list with signatures and initials of all authorized persons will be filed in the study site file and the trial master file, respectively.

Documented medical histories and narrative statements relative to the participant's progress during the study will be maintained. These records will also include the following: originals or copies of laboratory and other medical test results which must be kept on file with the individual participant's eCRF.

The investigators assure to perform a complete and accurate documentation of the participant data in the eCRF.

Essential documents, as well as any patient files and source data must be retained for at least 10 years after the regular end or a premature termination of the respective study (KlinV Art. 45).

14.1.2 Specification of Source Documents

The following documents are considered source data, including but not limited to:

- Electronic KISIM chart information
- Documents from other health care facilities
- Study worksheets

Source data will be available at the site to document the existence of the study participants and substantiate the integrity of study data collected. Source data must include the original documents relating to the study, as well as the medical treatment and medical history of the participant.

The following information (at least but not limited to) should be included in the source documents:

- Demographic data (age, sex)
- Inclusion and Exclusion Criteria
- Participation in study and signed and dated Informed Consent Forms
- Visit dates
- Medical history and physical examination details
- Key efficacy and safety data (as specified in the investigation plan)
- AEs and concomitant medication
- Results of relevant examinations
- Laboratory printouts
- Usage of study device (details)
- Reason for premature discontinuation
- Randomization number

No data will be directly entered in the CRF.

14.1.1 Record Keeping / Archiving

All study data will be archived for a minimum of 10 years after study termination or premature termination of the clinical trial (Art. 45 Aufbewahrungspflicht). All source data will be stored at the study coordinators office.

14.2 Data Management

14.2.1 Data Management System

For data entry, data management, data storage and security an internet-based secure data base REDCap (Vanderbilt University, v9 1.0) developed in agreement to the Good Clinical Practice (GCP) guidelines provided by the Clinical Trials Center (CTC) Zurich will be used for this study. The developer frequently updates the software according to international standards. Coding and statistics will be performed with the statistical programming language R.

14.2.2 Data Security, Access and Back-up

The application will be operated on a dedicated server operated by the CTC Zurich. Data will be stored in an oracle data base, which will be installed on a local server at the University Hospital of Zurich. Same safety standards apply as for primary patient data protecting the data sets from unauthorized access of third parties, data loss (back-up) and network failover. Data entry via the application is web-based, and will be performed with the standard internet browser from a local personal (hospital) computer. Data will be transferred encrypted (SSL). Each user will receive a personalized REDCap account. Audit-trail information will be stored in the REDCap application for each data entry, data change, or data deletion including the time and name of the user. Data entered into the eCRF will undergo automatic plausibility checks where possible. An overview for the implemented plausibility checks will be available for each version of the eCRF.

14.2.3 Analysis and Archiving

REDCap features an interface for transmitting the data to common statistic programs (SAS, SPSS, R). After the termination of the study, the data and metadata will be extracted from the oracle database and stored on an optical data medium (DVD).

14.2.4 Electronic and Central Data Validation

Audit-trail information will be stored in the REDCap application for each data entry, data change, or data deletion including the time and name of the user. Data entered into the eCRF will undergo automatic plausibility checks where possible. An overview for the implemented plausibility checks will be available for each version of the eCRF. The REDCap application offers the option to provide a reading-only account for monitoring purposes.

14.3 Monitoring

Regular monitoring visits at the investigator’s site prior to the start and during the study will help to follow up the progress of the clinical study, to assure utmost accuracy of the data and to detect possible errors at an early time point. The Sponsor-Investigator organizes professional independent monitoring for the study. The investigator's site will collaborate with the Clinical Trials Center (CTC) of the University Hospital Zurich (Rämistrasse 100 / MOU2, 8091 Zürich) to ensure regular monitoring. According to the CTC's Monitoring SOP the extent and nature of monitoring activities based on the objective and design of the study will be defined in a separate study specific Monitoring Plan.

All original data including all patient files, progress notes and copies of laboratory and medical test results will be available for monitoring. Source data includes study eligibility, inclusion / exclusion criteria, baseline demographics, primary efficacy endpoint, primary safety endpoints and SAE/SADE.

14.4 Audits and Inspections

A quality assurance audit/inspection of this study may be conducted by the Competent Authority or Competent Ethic Committee, respectively. The quality assurance auditor/inspector will have access to all medical records, the investigator's study related files and correspondence, and the informed consent documentation that is relevant to this clinical study.

The investigator will allow the persons being responsible for the audit or the inspection to have access to the source data/documents and to answer any questions arising. All involved parties will keep the patient data strictly confidential.

14.5 Confidentiality, Data Protection

Direct access to online source documents will be permitted for purposes of monitoring, audits and inspections. The investigator-sponsor, all investigators, the members of the clinical event committee, the study coordinator, and the bio-statistician will have access to the investigation plan and data sets during and after the study. The bio-statistician will have access to the statistical code.

14.6 Data and safety monitoring board

The data and safety monitoring board (DSMB) will be appointed by the data and safety monitoring officer (DSMO). At least one member of the DSMB will be a statistician. The primary mandate of the DSMB is to protect patient safety. The DSMB will review the results of safety assessment conducted after the completion of 12-month follow-up of multiples of 150 patients. The DSMB can recommend continuation or termination of the study based on the evaluation of the results.

15 PUBLICATION AND DISSEMINATION POLICY

By signing the clinical trial protocol, the investigator agrees on the use of the results of this clinical trial for the national and international registration of the product for specific indications, publication and information for medical and industrial professionals. If necessary, the authorities will be notified of the investigator's name, address, qualifications and extent of involvement. The findings of this clinical trial including the interim analysis will be published in a scientific journal and will be reported in Clinicaltrials.gov. Publication of clinical trial results requires mutual agreement between the investigators and the sponsor. Any publication of the clinical trial data by the sponsor or investigators will be wholly consistent with the integrated report in accordance with the ethical principles of the Declaration of Helsinki.
All publications will follow the Uniform Requirements for Manuscripts Submitted to Biomedical Journals (www.icmje.org, October 2008).

16 FUNDING AND SUPPORT

16.1 Funding

This investigator-initiated study will funded by the

University Hospital Zurich
Clinic of Angiology
Rämistrasse 100
CH 8091 Zürich
Switzerland

16.2 Other Support

Concept Medical will provide partial funding to support the conduction of this trial.

Concept Medical
Mariner ST, STE 200
Tampa, FL 33609
United States of America

17 INSURANCE

Insurance is covered by “Versicherung für klinische Versuche und nicht-klinische Versuche“ by Zürich Versicherungs-Gesellschaft AG (Policy no.: 14.970.888). Any damage developed in relation to study participation is covered by this insurance. So as not to forfeit their insurance cover, the participants themselves must strictly follow the instructions of the study personnel. Participants must not be involved in any other medical treatment without permission/information of the principal investigator (emergency excluded). Medical emergency treatment must be reported immediately to the investigator. The investigator must also be informed instantly, in the event of health problems or other damages during or after the course of study intervention. The investigator will allow delegates of the insurance company to have access to the source data/documents as necessary to clarify a case of damage related to study participation. All involved parties will keep the patient data strictly confidential. A copy of the insurance certificate will be placed in the Investigator’s Site File.

18 APPENDICES

18.1 Fontaine classification

Stage	Symptoms
I	Asymptomatic
IIa	Non-disabling intermittent claudication
IIb	Disabling intermittent claudication
III	Ischemic rest pain
IV	Ulceration or gangrene

Table 10: Fontaine classification

18.2 Rutherford classification for chronic limb ischemia

Category	Symptoms
0	Asymptomatic
1	Mild claudication
2	Moderate claudication
3	Severe claudication
4	Ischemic rest pain
5	Minor tissue loss
6	Ulceration or gangrene

Table 11: Rutherford classification for chronic limb ischemia

18.3 Rutherford classification for acute limb ischemia

Category	Definition	Prognosis	Examination	
			Sensory Loss	Muscle Weakness
I	Viable	Not immediately threatened	None	None
IIa	Threatened Marginally	Salvageable with prompt treatment	Minimal (Toes)	None
IIb	Threatened Immediately	Salvageable with immediate treatment	More than toes, pain at rest	Mild to moderate
III	Irreversible	Major permanent tissue loss	Anesthetic	Paralysis

Table 12: Rutherford classification for acute limb ischemia

19 REFERENCES

1. Song P, Rudan D, Zhu Y, et al. Global, regional, and national prevalence and risk factors for peripheral artery disease in 2015: an updated systematic review and analysis. *Lancet Glob Health.* 2019;7(8):e1020-e1030.
2. European Stroke O, Tendera M, Aboyans V, et al. ESC Guidelines on the diagnosis and treatment of peripheral artery diseases: Document covering atherosclerotic disease of extracranial carotid and vertebral, mesenteric, renal, upper and lower extremity arteries: the Task Force on the Diagnosis and Treatment of Peripheral Artery Diseases of the European Society of Cardiology (ESC). *Eur Heart J.* 2011;32(22):2851-2906.
3. Dake MD, Ansel GM, Jaff MR, et al. Paclitaxel-eluting stents show superiority to balloon angioplasty and bare metal stents in femoropopliteal disease: twelve-month Zilver PTX randomized study results. *Circ Cardiovasc Interv.* 2011;4(5):495-504.
4. Rosenfield K, Jaff MR, White CJ, et al. Trial of a Paclitaxel-Coated Balloon for Femoropopliteal Artery Disease. *N Engl J Med.* 2015;373(2):145-153.
5. Scheinert D, Duda S, Zeller T, et al. The LEVANT I (Lutonix paclitaxel-coated balloon for the prevention of femoropopliteal restenosis) trial for femoropopliteal revascularization: first-in-human randomized trial of low-dose drug-coated balloon versus uncoated balloon angioplasty. *JACC Cardiovasc Interv.* 2014;7(1):10-19.
6. Zeller T, Baumgartner I, Scheinert D, et al. Drug-eluting balloon versus standard balloon angioplasty for infrapopliteal arterial revascularization in critical limb ischemia: 12-month results from the IN.PACT DEEP randomized trial. *J Am Coll Cardiol.* 2014;64(15):1568-1576.
7. Tepe G, Laird J, Schneider P, et al. Drug-coated balloon versus standard percutaneous transluminal angioplasty for the treatment of superficial femoral and popliteal peripheral artery disease: 12-month results from the IN.PACT SFA randomized trial. *Circulation.* 2015;131(5):495-502.
8. Muller-Hulsbeck S, Keirse K, Zeller T, Schroe H, Diaz-Cartelle J. Twelve-Month Results From the MAJESTIC Trial of the Eluvia Paclitaxel-Eluting Stent for Treatment of Obstructive Femoropopliteal Disease. *J Endovasc Ther.* 2016;23(5):701-707.
9. Rastan A, Tepe G, Krankenberg H, et al. Sirolimus-eluting stents vs. bare-metal stents for treatment of focal lesions in infrapopliteal arteries: a double-blind, multi-centre, randomized clinical trial. *Eur Heart J.* 2011;32(18):2274-2281.
10. Klumb C, Lehmann T, Aschenbach R, Eckardt N, Teichgräber U. Benefit and risk from paclitaxel-coated balloon angioplasty for the treatment of femoropopliteal artery disease: A systematic review and meta-analysis of randomised controlled trials. *EClinicalMedicine.* 2019.
11. Katsanos K, Spiliopoulos S, Kitrou P, Krokidis M, Karnabatidis D. Risk of Death Following Application of Paclitaxel-Coated Balloons and Stents in the Femoropopliteal Artery of the Leg: A Systematic Review and Meta-Analysis of Randomized Controlled Trials. *J Am Heart Assoc.* 2018;7(24):e011245.
12. Lemos PA, Farooq V, Takimura CK, et al. Emerging technologies: polymer-free phospholipid encapsulated sirolimus nanocarriers for the controlled release of drug from a stent-plus-balloon or a stand-alone balloon catheter. *EuroIntervention.* 2013;9(1):148-156.
13. Abu Dabrh AM, Steffen MW, Undavalli C, et al. The natural history of untreated severe or critical limb ischemia. *J Vasc Surg.* 2015;62(6):1642-1651 e1643.
14. Laird JR, Jain A, Zeller T, et al. Nitinol stent implantation in the superficial femoral artery and proximal popliteal artery: twelve-month results from the complete SE multicenter trial. *J Endovasc Ther.* 2014;21(2):202-212.
15. Klaphake S, de Leur K, Thijsse W, et al. Reinterventions after Endovascular Revascularization in Elderly Patients with Critical Limb Ischemia: An Observational Study. *Ann Vasc Surg.* 2018;53:171-176.
16. Grip O, Wanhainen A, Acosta S, Bjorck M. Long-term Outcome after Thrombolysis for Acute Lower Limb Ischaemia. *Eur J Vasc Endovasc Surg.* 2017;53(6):853-861.
17. Byrne RM, Taha AG, Avgerinos E, Marone LK, Makaroun MS, Chaer RA. Contemporary outcomes of endovascular interventions for acute limb ischemia. *J Vasc Surg.* 2014;59(4):988-995.

18. Ouriel K, Veith FJ, Sasahara AA. A comparison of recombinant urokinase with vascular surgery as initial treatment for acute arterial occlusion of the legs. Thrombolysis or Peripheral Arterial Surgery (TOPAS) Investigators. *N Engl J Med.* 1998;338(16):1105-1111.
19. Hess CN, Rogers RK, Wang TY, et al. Major Adverse Limb Events and 1-Year Outcomes After Peripheral Artery Revascularization. *J Am Coll Cardiol.* 2018;72(9):999-1011.
20. Anand SS, Bosch J, Eikelboom JW, et al. Rivaroxaban with or without aspirin in patients with stable peripheral or carotid artery disease: an international, randomised, double-blind, placebo-controlled trial. *Lancet.* 2018;391(10117):219-229.
21. Fashandi AZ, Mehaffey JH, Hawkins RB, Kron IL, Upchurch GR, Jr., Robinson WP. Major adverse limb events and major adverse cardiac events after contemporary lower extremity bypass and infrainguinal endovascular intervention in patients with claudication. *J Vasc Surg.* 2018;68(6):1817-1823.
22. Mehaffey JH, Hawkins RB, Fashandi A, et al. Lower extremity bypass for critical limb ischemia decreases major adverse limb events with equivalent cardiac risk compared with endovascular intervention. *J Vasc Surg.* 2017;66(4):1109-1116 e1101.
23. Bonaca MP, Nault P, Giugliano RP, et al. Low-Density Lipoprotein Cholesterol Lowering With Evolocumab and Outcomes in Patients With Peripheral Artery Disease: Insights From the FOURIER Trial (Further Cardiovascular Outcomes Research With PCSK9 Inhibition in Subjects With Elevated Risk). *Circulation.* 2018;137(4):338-350.
24. Anand SS, Caron F, Eikelboom JW, et al. Major Adverse Limb Events and Mortality in Patients With Peripheral Artery Disease: The COMPASS Trial. *J Am Coll Cardiol.* 2018;71(20):2306-2315.
25. Gamble C, Krishan A, Stocken D, et al. Guidelines for the Content of Statistical Analysis Plans in Clinical Trials. *JAMA.* 2017;318(23):2337-2343.
26. D'Agostino RB, Sr., Massaro JM, Sullivan LM. Non-inferiority trials: design concepts and issues - the encounters of academic consultants in statistics. *Stat Med.* 2003;22(2):169-186.
